# Total Synthesis and Structural Reassignment of Laingolide A

**DOI:** 10.3390/md19050247

**Published:** 2021-04-27

**Authors:** Fusong Wu, Tao Zhang, Jie Yu, Yian Guo, Tao Ye

**Affiliations:** 1 State Key Laboratory of Chemical Oncogenomics, Peking University Shenzhen Graduate School, Shenzhen 518055, China; wufusong@pku.edu.cn (F.W.); 1901212908@pku.edu.cn (T.Z.); yujie0701@pku.edu.cn (J.Y.); 2 School of Biotechnology and Health Sciences, Wuyi University, Jiangmen 529020, China

**Keywords:** total synthesis, structural reassignment, laingolide A

## Abstract

The asymmetric total synthesis of four diastereomers of laingolide A was achieved, which led to the unambiguous assignment of the stereochemistry of the natural product. The salient features of the convergent, fully stereocontrolled approach were a copper-catalysed stereospecific Kumada-type coupling, a Julia-Kocienski olefination and an RCM/alkene migration sequence to access the desired macrocyclic enamide.

## 1. Introduction

The modern structure determination of unknown natural products remains a challenging problem, especially when a small quantity of the natural compound is available, limiting the full possibility of the modern spectroscopic methods. For this reason, total synthesis has played a major role in the structure elucidation and revision of complex natural products for a long time. In the absence of a firm structural assignment, a combination of the stereochemical logic of the synthesis and spectroscopic comparison could be employed as tools to establish the correct structure of natural products [1,2,3,4]. Previous work in our group led to the reassignment of the configuration of a number of marine natural products [5,6,7,8,9,10,11]. These results encouraged us to embark upon the synthesis of other natural products with unknown configurations. We describe herein the determination of the complete relative and absolute stereochemistries of (+)-laingolide A and the total synthesis of this material. 

**Scheme 1:**
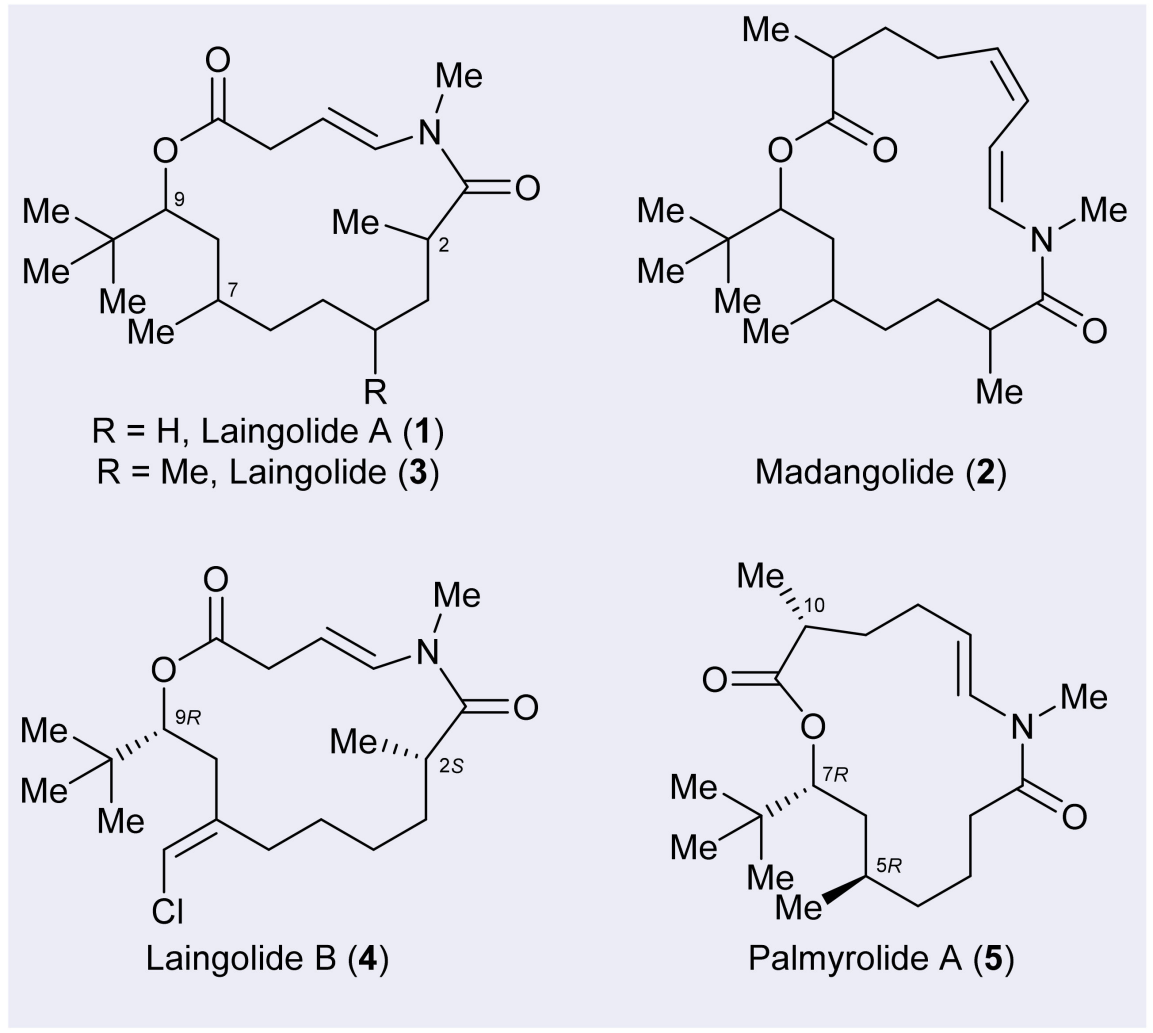
Structures of laingolide A (**1**), madangolide (**2**), laingolide (**3**), laingolide B (**4**) and palmyrolide A (**5**).

Laingolide A (**1**), along with madangolide (**2**), was isolated in 1999 from the marine cyanobacterium *Lyngbya bouillonii* collected in Papua New Guinea [12]. The very same blue-green algae also produced laingolide (**3**) (Scheme 1), which was disclosed as the first member of the novel macrolide family [13]. Additionally, another chlorinated analogue, laingolide B (**4**), was isolated in 2010 by Luesch and co-workers from the same species of bacteria collected in Apra Harbor, Guam [14]. The planar structures of the laingolides were established using a combination of detailed 1D and 2D NMR analysis. However, these macrolides underwent degradation over time, which hampered progress towards complete assignments of their absolute stereochemistry. In 2010, Gerwick and co-workers reported the isolation of palmyrolide A (**5**), a structurally closely related 15-member macrolide from a cyanobacterial assemblage comprised of *Leptolyngbya* and *Oscillatoria* species collected at Palmyra Atoll, south of Hawaii [6]. One of the absolute configurations present in palmyrolide A was correctly assigned upon its initial isolation [15], and the absolute configurations of the remaining stereocentres were later established via total syntheses [16,17,18,19,20,21,22,23,24]. The structurally intriguing laingolides have attracted considerable attention from the synthetic community [25,26,27]. In 2018, Dai and co-workers reported the first total synthesis of laingolide B and unambiguously assigned the absolute configuration as depicted in structure **4 [27]**. Laingolide A (**1**) was isolated from the bacteria collected in a different location and eleven years before the isolation of laingolide B (**4**). Laingolide A contains an extra undefined stereogenic centre located at C-(7), whose absolute structure remained elusive. Furthermore, laingolide A (**1**) bears a close resemblance to palmyrolide A (**5**); specifically, these two macrolides contain a *tert*-butyl carbinol and a methyl group beta to the *tert*-butyl substituted stereocenter. 

It would be interesting to find out whether the absolute configurations of laingolide A (**1**) were likely the same based on the possible similar biogenesis to palmyrolide A (**5**). Through the completion of the synthesis of four diastereomers, we shall be able to provide conclusive evidence for the absolute and relative stereochemistry of laingolide A (**1**) [28].

**Scheme 2:**
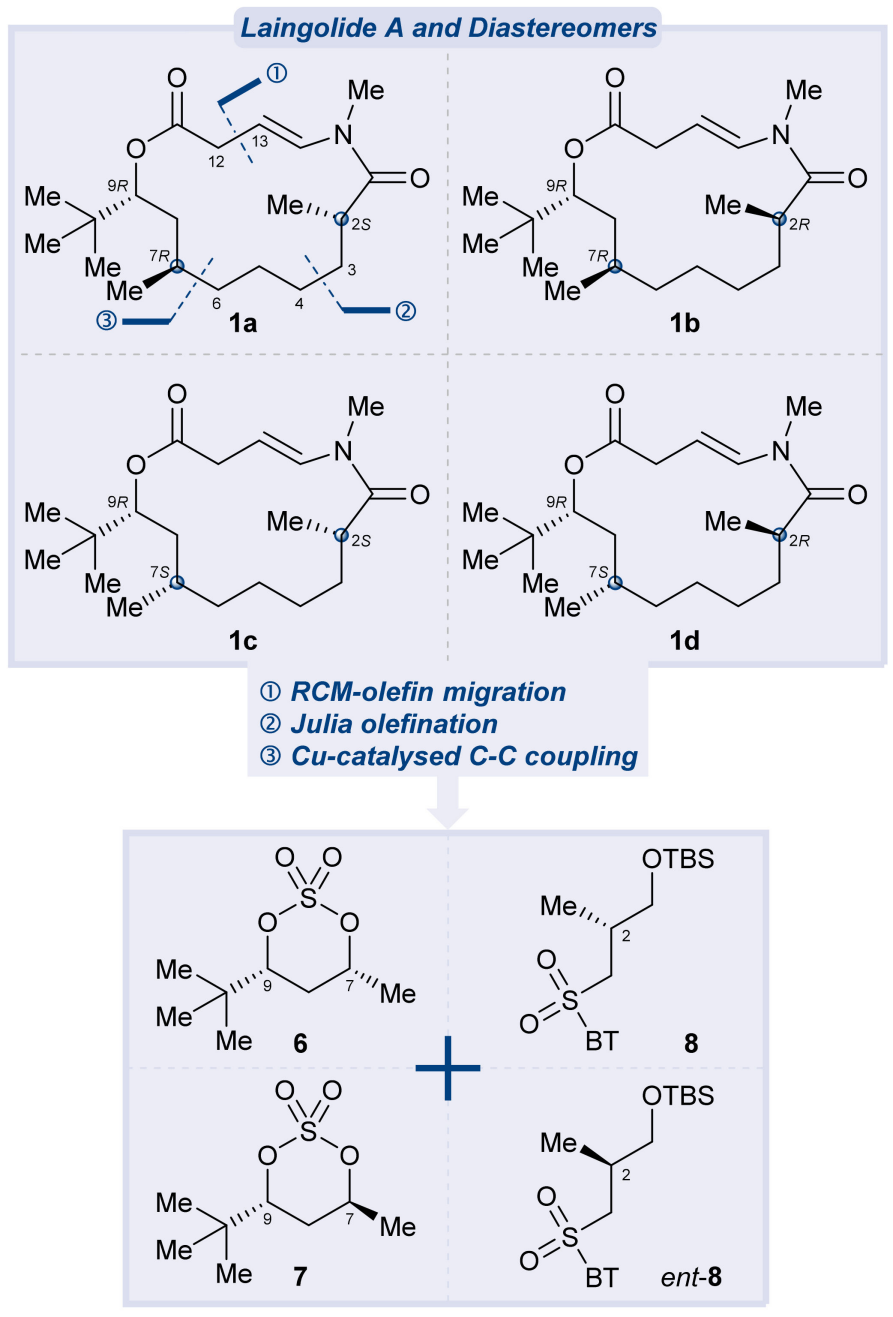
Retrosynthetic analysis of laingolide A and its diastereomers (**1a**–**d**).

Structurally, laingolide A features a 15-member macrocyclic core, which is composed of a sterically encumbered ester derived from a *tert*-butyl carbinol, a *trans*-*N*-methyl enamide subunit and two chiral methyl appendages. At the outset of this synthetic venture, our primary objective was to rapidly access four diastereomers (Scheme 2) and thus conclusively establish the absolute configuration of laingolide A. With this in mind, we opted for a modular and flexible approach as we pondered its retrosynthetic analysis (Supplementary Materials) (Scheme 2) [29,30,31,32,33,34,35,36]. We envisioned that the four diastereomers of laingolide A could be constructed from fragments **6**, **7**, **8** and *ent*-**8** via three key transformations, as illustrated in Scheme 2. This highly convergent strategy relied on a ring-closing metathesis (RCM) at C-12 and C-13 to deliver the macrocycle and olefin migration to forge the enamide moiety at the final stage [19,22,27]. Both copper-catalysed Kumada-type coupling [37] of cyclic sulfate esters and Julia-Kocienski olefination [38,39,40] were then employed to construct the RCM precursor.

## 2. Results

**Scheme 3:**
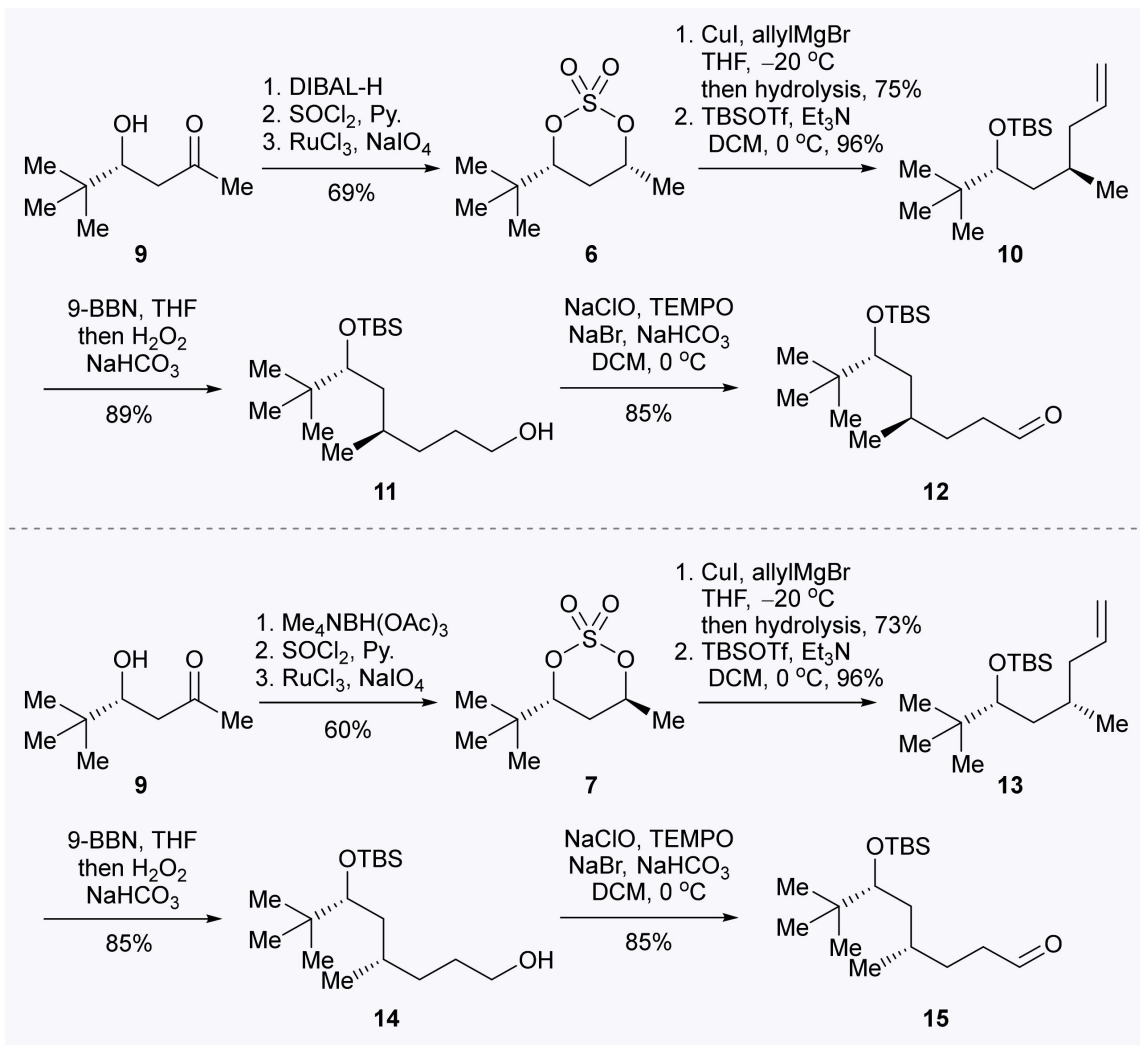
Synthesis of aldehydes **12** and **15.**

The synthesis towards the chiral aldehydes (Scheme 3) commenced with the known chiral 1,3-hydroxy ketone **9 [23]**, which was prepared via List’s proline-catalysed aldolisation between acetone and pivalaldehyde [41]. A three-step sequence [23] was employed to elaborate 1,3-hydroxy ketone **9** into cyclic sulfate ester **6** involving (1) the *syn* reduction of ketone **9** with DIBAL-H, (2) conversion of the *syn*-diol into the corresponding sulfite with thionyl chloride and (3) oxidation of the cyclic sulfite with NaIO_4_ in the presence of catalytic amounts of RuCl_3_. Nucleophilic ring-opening of the cyclic sulfate **6** using a mixed organometallic reagent derived from allylmagnesium chloride and stoichiometric quantities of copper(I) iodide has already been reported [42]. Recently, a catalytic version of this reaction, also termed C(sp^3^)-C(sp^3^) Kumada-type coupling of cyclic sulfate esters was reported [37]. We opted to incorporate this catalytic reaction into our synthesis. Thus, treatment of cyclic sulfate **6** and 10 mol% of cuprous iodide in THF with allyl magnesium bromide at −20 °C followed by hydrolysis of the corresponding intermediate gave rise to the corresponding alcohol with a 75% yield with a >95:5 diastereomeric ratio (dr), as determined using ^1^H NMR spectroscopy. This reaction occurred at the least-hindered site, with the complete inversion of the configuration at that centre. Protection of the resulting alcohol with TBSOTf (*tert*-butyldimethylsilyl trifluoromethanesulfonate) and triethylamine afforded TBS ether **10** with a 96% yield. Hydroboration of **10** with 9-borabicyclo[3.3.1] nonane (9-BBN) and oxidation of the resulting organoborane (NaHCO_3_, H_2_O_2_) furnished alcohol **11** with an 89% yield, which in turn was subjected to oxidation with TEMPO, NaOCl and NaBr [43] to provide aldehyde **12** with an 85% yield. In parallel, the hydroxyl-directed antireduction of hydroxy ketone **9** with the Evans–Carreira protocol [44] proceeded smoothly to furnish the desired 1,3-*anti* diol with a diastereomeric ratio of 5:1 (determined by ^1^H NMR of the crude reaction mixture). These diastereomers were separated using flash chromatography and the major one was used in subsequent reactions. The *anti*-substituted cyclic sulfate **7** was prepared with a 60% yield over three steps using the same procedure as described for **6**. The elaboration of the substituted cyclic sulfate **7** into aldehyde **15** was accomplished in a way similar to that described for the preparation of aldehyde **12**.

**Scheme 4:**
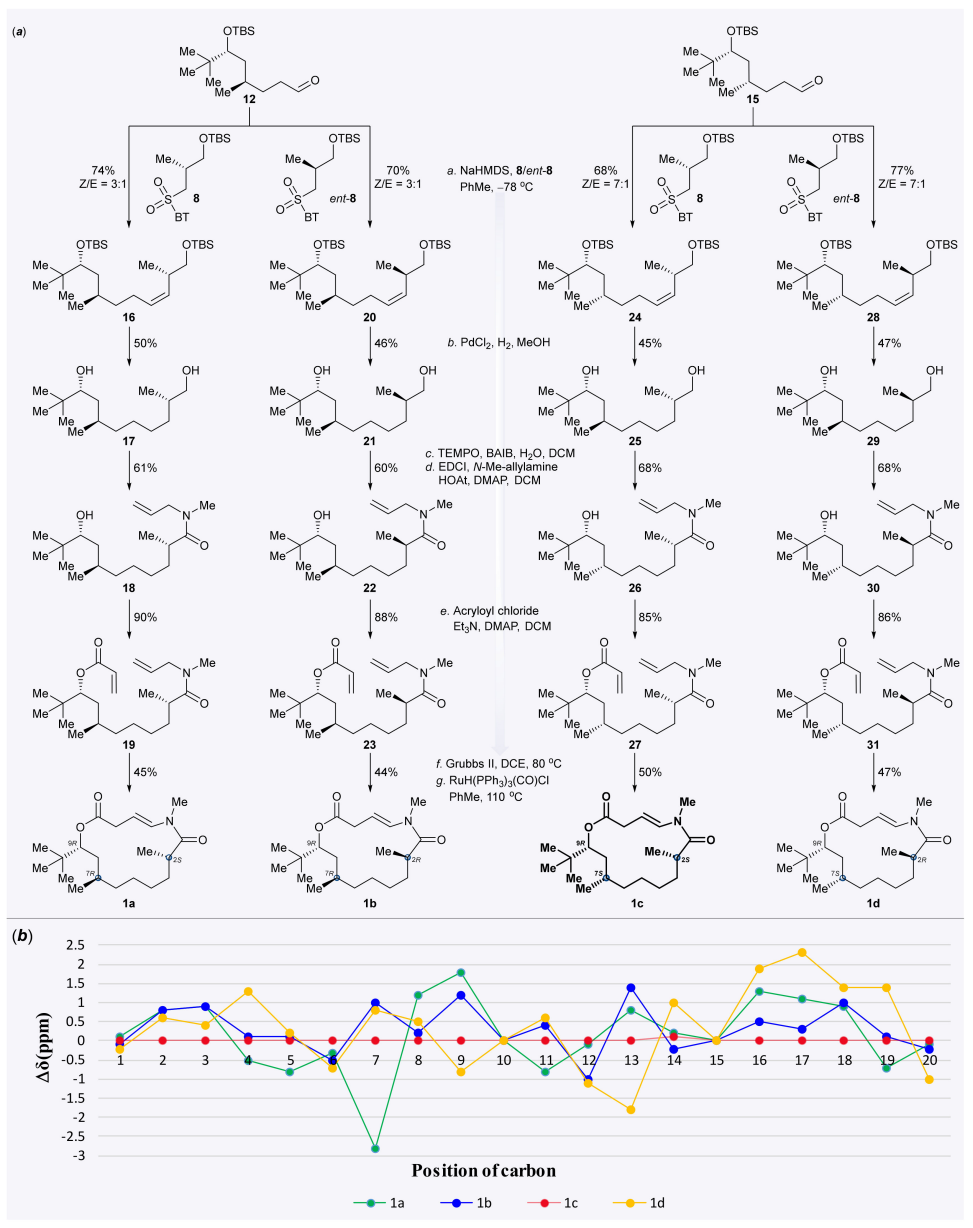
(**a**) Total synthesis of laingolide A and its diastereomers (**1a**–**d**); (**b**) comparison of the ^13^C NMR data of the synthetic compounds (**1a**–**d**) with the naturally occurring laingolide A.

With a reliable route to useful quantities of the required aldehydes **12** and **15** in hand, our efforts turned to the divergent total synthesis of laingolide A (Scheme 4). This required the combination of aldehydes **12** and **15** separately with BT-sulfones **8** and then with *ent*-**8 [45]** (Scheme 4a). Under the optimum conditions investigated, each aldehyde (**12** or **15**) underwent condensation with 1.2 molar equivalent of sulfone (**8** or *ent*-**8**) treated with 1.2 molar equivalent of NaHMDS in toluene at −78 °C to afford the corresponding alkene as a mixture of geometrical isomers (Z:E ≈ 3–7:1) in high yield. Next, each of the resultant internal alkenes (**16**, **20**, **24**, **28**) was separately subjected to palladium-chloride-mediated hydrogenation in ethanol with the concomitant removal of the TBS ethers that furnish the corresponding diol (**17**, **21**, **25**, **29**) [46]. The primary alcohol of the above diol was selectively oxidised with TEMPO in the presence of bis-acetoxyiodobenzene (BAIB) [47] and the resulting carboxylic acid was then coupled with the *N*-methylallylamine by utilising EDCI-HOAt and DMAP as a base to provide the corresponding amide (**18**, **22**, **26**, **30**). For the conversion to the required diene (**19**, **23**, **27**, **31**), each amide alcohol was separately acylated with acryloyl chloride in the presence of triethylamine and DMAP. The four diastereomeric dienes (**19**, **23**, **27**, **31**) were separately subjected to ring-closing metathesis using the second-generation Grubbs catalyst (G-II) to afford the corresponding unsaturated macrolactone as isomeric mixtures, which were subsequently treated with RuH(PPh_3_)_3_(CO)Cl in refluxing toluene [48] to furnish the desired enamides **1a**–**d** with good yields. The comparison of the spectral data of **1a**–**d** with the reported spectra of laingolide A was informative. Compound **1a**, featuring a C(7)-*R* methyl beta to the C(9)-*R*-*tert*-butyl-substituted stereocenter, the same as that of natural palmyrolide A (**5**), did not match the literature values reported for the laingolide A. This suggested that the biogenesis of the laingolide A and palmyrolide A might follow different pathways or that C7 is epimerized at some stage of the biosynthesis of laingolide A (or of palmyrolide A). It was clear from comparing the ^13^C NMR data (Scheme 4b) that diastereomer **1c** represented the correct structure of natural laingolide A. The absolute stereochemical assignment of laingolide A was thus assigned as 2S,7S,9R, as shown in **1c** (Scheme 4).

## 3. Materials and Methods

### 3.1. General Information

All reactions were conducted in flame-dried or oven-dried glassware under an atmosphere of dry nitrogen or argon. Oxygen and/or moisture-sensitive solids and liquids were transferred appropriately. The concentration of solutions in vacuo was accomplished using a rotary evaporator fitted with a water aspirator. Residual solvents were removed under a high vacuum (0.1–0.2 mm Hg). All reaction solvents were purified before use: tetrahydrofuran (THF) was distilled from Na/benzophenone. Toluene was distilled over molten sodium metal. Dichloromethane (DCM), 1,2-dichloroethane (DCE) and trimethylamine (Et_3_N) were distilled from CaH_2_. Methanol (MeOH) was distilled from Mg/I_2_. The reagents were purchased at the highest commercial quality and used without further purification, unless otherwise stated. Flash column chromatography was performed using the indicated solvents on E. Qingdao silica gel 60 (230–400 mesh ASTM). Reactions were monitored using thin-layer chromatography (TLC), which was carried out using pre-coated sheets (Qingdao silica gel 60-F250, 0.2 mm, Tsingtao, China). Compounds were visualised with UV light, iodine and ceric ammonium molybdate stainor phosphomolybdic acid in EtOH. The ^1^H NMR spectra were recorded on Bruker Avance 300 MHz, Avance 400 MHz or Avance 500 MHz spectrometers (Karlsruhe, Germany). Chemical shifts were reported in parts per million (ppm), relative to either a tetramethylsilane (TMS) internal standard or the signals due to the solvent (Supplementary Materials). The following abbreviations are used to describe the spin multiplicity: s = singlet, d = doublet, t = triplet, q = quartet, qn = quintet, m = multiplet, br = broad, dd = doublet of doublets, dt = doublet of triplets, dq = doublet of quartets, ddd = doublet of doublet of doublets; other combinations are derived from those listed above. Coupling constants (*J*) are reported in hertz (Hz) for corresponding solutions, and chemical shifts are reported as parts per million (ppm) relative to residual CHCl_3_ δH (7.26 ppm). ^13^C Nuclear magnetic resonance spectra were recorded using a 75 MHz, 100 MHz or 125 MHz spectrometer (Karlsruhe, Germany) for corresponding solutions, and chemical shifts are reported as parts per million (ppm) relative to residual CDCl_3_ δC (77.16 ppm) (Supplementary Materials). High-resolution mass spectra were measured on an ABI Q-star Elite (Beijing, China). Optical rotations were recorded on a Rudolph AutoPol-I polarimeter (Shanghai, China) at 589 nm with a 50 mm cell. Data are reported as follows: specific rotation (*c* (g/100 mL), solvent).

### 3.2. General Experimental Procedures

#### 3.2.1. Synthesis of (3R,5R)-2,2,5-trimethyloct-7-en-3-ol (S11)

To a solution of cyclic sulfate **6** (620 mg, 2.98 mmol, 1.0 eq.) and CuI (57 mg, 0.3 mmol, 0.1 eq.) in dry THF (1 mL) at −20 °C, allylmagnesium bromide (1.0 M in THF, 14.9 mL, 14.9 mmol, 5.0 eq.) was added under an argon atmosphere. The purple-colored reaction mixture was allowed to stir at −20 °C for 7 h before it was allowed to warm to room temperature and then become concentrated in vacuo. The solid residue was redissolved in Et_2_O (30 mL) and treated with 20% aqueous H_2_SO_4_ (10 mL) solution. The contents of the flask were then stirred vigorously for another 12 h before the phases were separated. The aqueous layer was extracted with Et_2_O (3 × 30 mL). The combined organic layers were dried over anhydrous sodium sulfate, filtered and concentrated under reduced pressure. Purification of the crude product was performed using flash chromatography on silica gel (hexanes/EtOAc = 20:1) to afford alcohol **S11** (380 mg, 75%) as a colorless oil. TLC: R*_f_* = 0.6 (hexanes/EtOAc = 10:1), iodine and PMA stain. $\alpha_{D}^{23}$ = +36.0 (*c* 1.00, CHCl_3_). ^1^H NMR (500 MHz, CDCl_3_) δ 5.86–5.67 (m, 1H), 5.14–4.91 (m, 2H), 3.29 (dd, *J* = 10.3, 1.6 Hz, 1H), 2.30–2.13 (m, 1H), 1.92–1.82 (m, 1H), 1.81–1.72 (m, 1H), 1.53 (s, 1H), 1.46–1.34 (m, 1H), 1.18 (ddd, *J* = 14.3, 10.3, 4.1 Hz, 1H), 0.93 (d, *J* = 6.7 Hz, 3H), 0.87 (s, 9H); ^13^C NMR (125 MHz, CDCl_3_) δ 137.2, 116.1, 77.7, 39.9, 38.6, 35.1, 29.9, 25.7, 21.0; HRMS (ESI) calculated for C_11_H_22_ONa^+^ [M + Na]^+^ 193.1563, found 193.1565.

#### 3.2.2. Synthesis of tert-butyldimethyl(((3R,5R)-2,2,5-trimethyloct-7-en-3-yl)oxy)silane (10)

To a solution of alcohol **S11** (2.8 g, 16.5 mmol, 1.0 eq.) in dry DCM (30 mL, 0.55 M), Et_3_N (33 mmol, 4.6 mL, 2.0 eq.) and TBSOTf (21.5 mmol, 4.9 mL, 1.3 eq.) were added at 0 °C. The reaction mixture was allowed to stir at 0 °C for 2 h before it was diluted with DCM (20 mL) and quenched with a saturated aqueous solution of NH_4_Cl (30 mL). The aqueous layer was extracted with DCM (3 × 50 mL). The combined organic layers were washed with brine (50 mL), dried over anhydrous sodium sulfate, filtered and concentrated under reduced pressure. Purification of the crude product was performed using flash chromatography on silica gel (hexanes) to furnish silyl ether **10** (4.5 g, 96%) as a colorless oil. TLC: R*_f_* = 0.95 (hexanes), iodine and PMA stain. $\alpha_{D}^{22}$ = +7.4 (*c* 0.02, CHCl_3_). ^1^H NMR (500 MHz, CDCl_3_) δ 5.83–5.73 (m, 1H), 5.04–4.99 (m, 2H), 3.34 (dd, *J* = 7.5, 2.8 Hz, 1H), 2.22–2.16 (m, 1H), 1.80–1.72 (m, 1H), 1.72–1.64 (m, 1H), 1.45 (ddd, *J* = 14.3, 9.3, 2.8 Hz, 1H), 1.21 (ddd, *J* = 14.3, 7.5, 4.2 Hz, 1H), 0.90 (d, *J* = 6.5 Hz, 3H), 0.90 (s, 9H), 0.86 (s, 9H), 0.07 (s, 3H), 0.06 (s, 3H); ^13^C NMR (125 MHz, CDCl_3_) δ 137.3, 116.1, 78.6, 41.0, 40.5, 36.0, 29.8, 26.6, 26.4, 20.9, 18.7, −3.2, −3.6; HRMS (ESI) calculated for C_17_H_36_OSi Na^+^ [M + Na]^+^ 307.2428, found 307.2425.

#### 3.2.3. Synthesis of (4R,6R)-6-((tert-butyldimethylsilyl)oxy)-4,7,7-trimethyloctan-1-ol (11)

To a solution of alkene **10** (0.5 g, 1.7 mmol, 1.0 eq.) in dry THF (3 mL, 0.17 M), 9-BBN (0.5 M in THF, 3.52 mmol, 7.0 mL, 2.0 eq.) was added at 0 °C under an argon atmosphere. The reaction mixture was stirred at room temperature for 8 h before a saturated aqueous solution of NaHCO_3_ (10 mL) and 30% H_2_O_2_ (2 mL) were added sequentially at 0 °C and stirred for another 12 h at room temperature. The aqueous layer was extracted with EtOAc (3 × 10 mL). The combined organic layers were washed with brine (10 mL), dried over anhydrous sodium sulfate, filtered and concentrated under reduced pressure. Purification of the crude product was performed using flash chromatography on silica gel (hexanes/EtOAc = 10:1) to afford alcohol **11** (473 mg, 89%) as a colorless oil. TLC: R*_f_* = 0.5 (hexanes/EtOAc = 4:1), PMA stain. $\alpha_{D}^{24}$ = +10.8 (*c* 0.01, CHCl_3_); ^1^H NMR (400 MHz, CDCl_3_) δ 3.63 (td, J = 6.6, 1.5 Hz, 2H), 3.30 (dd, *J* = 7.3, 2.9 Hz, 1H), 1.73–1.58 (m, 1H), 1.59–1.53 (m, 1H), 1.52–1.36 (m, 4H), 1.19 (ddd, *J* = 14.2, 7.3, 4.4 Hz, 1H), 1.06–0.94 (m, 1H), 0.90 (d, *J* = 6.6 Hz, 3H), 0.88 (s, 9H), 0.84 (s, 9H), 0.04 (s, 6H); ^13^C NMR (100 MHz, CDCl_3_) δ 78.5, 63.7, 41.7, 35.9, 32.2, 30.3, 29.9, 26.5, 26.3, 20.9, 18.6, −3.3, −3.7; HRMS (ESI) calculated for C_17_H_38_O_2_SiNa^+^ [M + Na]^+^ 325.2533, found 325.2528.

#### 3.2.4. Synthesis of (4R,6R)-6-((tert-butyldimethylsilyl)oxy)-4,7,7-trimethyloctanal (12)

To a solution of alcohol **11** (1.0 g, 3.3 mmol, 1.0 eq.) and TEMPO (51 mg, 0.33 mmol, 0.1 eq.) in DCM (30 mL), a solution of NaBr (2.0 g, 19.8 mmol, 6.0 eq.) and NaHCO_3_ (1.7 g, 19.8 mmol, 6.0 eq.) were added in water (50 mL), followed by NaClO (1 M, 3.3 mL, 1.0 eq.) at 0 °C. The reaction mixture was stirred at 0 °C for 10 min and then quenched with a saturated aqueous solution of Na_2_S_2_O_3_ (3 mL) and extracted with DCM (3 × 30 mL). The combined organic layers were washed with brine (30 mL), dried over anhydrous sodium sulfate, filtered and concentrated under reduced pressure. Purification of the crude product was performed using flash chromatography on silica gel (hexanes/EtOAc = 20:1) to afford aldehyde **12** (842 mg, 85%) as a colorless oil. TLC: R*_f_* = 0.6 (hexanes/EtOAc = 10:1), PMA stain. $\alpha_{D}^{27}$ = +11.5 (*c* 0.01, CHCl_3_). ^1^H NMR (500 MHz, CDCl_3_) δ 9.78 (t, *J* = 1.8 Hz, 1H), 3.30 (dd, *J* = 7.3, 2.9 Hz, 1H), 2.58–2.44 (m, 1H), 2.40–2.29 (m, 1H), 1.90–1.71 (m, 1H), 1.65–1.53 (m, 1H), 1.44 (ddd, *J* = 14.3, 9.0, 2.8 Hz, 1H), 1.34–1.25 (m, 1H), 1.22 (ddd, *J* = 14.3, 7.1, 4.3 Hz, 1H), 0.90 (d, *J* = 6.7 Hz, 3H), 0.88 (s, 9H), 0.84 (s, 9H), 0.04 (s, 3H), 0.02 (s, 3H); ^13^C NMR (125 MHz, CDCl_3_) δ 202.7, 78.4, 41.6, 41.5, 36.0, 29.7, 28.0, 26.5, 26.3, 20.7, 18.6, −3.3, −3.7; HRMS (ESI) calculated for C_17_H_38_O_2_SiNa^+^ [M + Na]^+^ 323.2377, found 323.2367.

#### 3.2.5. Synthesis of (4R,6S)-4-(tert-butyl)-6-methyl-1,3,2-dioxathiane 2,2-dioxide (7)

To a solution of Me_4_NHB(OAc)_3_ (13.7 g, 52.1 mmol, 5.0 eq.) in anhydrous CH_3_CN (25 mL) and anhydrous AcOH (15 mL) at −40 °C, a solution of **9 [23]** (1.5 g, 10.4 mmol, 1.0 eq.) was added in anhydrous CH_3_CN (15 mL). The reaction mixture was stirred at −40 °C for 12 h, allowed to warm to ambient temperature, poured into a saturated aqueous solution of Na_2_CO_3_ (80 mL) and then extracted with EtOAc (3 × 100 mL). The combined organic layers were washed with brine (10 mL), dried over anhydrous sodium sulfate, filtered and concentrated under reduced pressure. Purification of the crude product was performed using flash chromatography on silica gel (hexanes/EtOAc = 10:1–5:1) to afford diol **S13** (1.1 g, 71%) as a white solid. [43] ^1^H NMR analysis revealed the presence of a 5:1 ratio of *anti*/*syn*.

To a solution of *anti*-diol **S13** (3.15 g, 21.5 mmol, 1.0 eq.) in dry DCM (200 mL, 0.22 M), pyridine (17.3 mL, 215.0 mmol, 10.0 eq.) and SOCl_2_ (7.9 mL, 108.0 mmol, 5.0 eq.) were added sequentially at 0 °C. The reaction mixture was allowed to stir at 0 °C for 45 min before it was quenched by the addition of water (50 mL) and then extracted with DCM (3 × 100 mL). The combined organic layers were washed with saturated aqueous KHSO_4_ solution (50 mL), followed by saturated aqueous NaHCO_3_ solution (60 mL), dried over anhydrous sodium sulfate, filtered and concentrated under reduced pressure to afford the crude sulfite, which was used in the next step without further purification. To a solution of the crude sulfite in a mixture of H_2_O/MeCN/CCl_4_ (200 mL:200 mL:100 mL), RuCl_3_·nH_2_O (562 mg, 2.15 mmol, 0.1 eq.) and NaIO_4_ (6.9 g, 32.3 mmol, 1.5 eq.) were added. The biphasic reaction mixture was vigorously stirred at room temperature for 2 h before it was diluted with Et_2_O (60 mL) and quenched with a saturated aqueous solution of NaHCO_3_ (100 mL). The aqueous layer was extracted with Et_2_O (3 × 200 mL). The combined organic layers were dried over anhydrous sodium sulfate, filtered and concentrated under reduced pressure. Purification of the crude product was performed using flash chromatography on silica gel (hexanes/EtOAc = 10:1–5:1) to afford *anti*-cyclic sulfate **7** (3.76 g, 84% for two steps) as an amorphous white solid. The spectral data are in accordance with those reported in literature for its enantiomer [23]. TLC: R*_f_* = 0.5 (hexanes/ EtOAc = 4:1), PMA stain. $\alpha_{D}^{25}$= −0.06 (*c* 0.03, CHCl_3_). ^1^H NMR (400 MHz, CDCl_3_) δ 4.94 (ddq, *J* = 6.7, 6.1, 4.4 Hz, 1H), 4.59 (dd, *J* = 11.4, 3.6 Hz, 1H), 2.30 (ddd, *J* = 14.2, 11.4, 6.1 Hz, 1H), 1.75 (ddd, *J* = 14.2, 4.4, 3.6 Hz, 1H), 1.63 (d, *J* = 6.8 Hz, 3H), 1.00 (s, 9H). ^13^C NMR (100 MHz, CDCl_3_) δ 88.6, 81.1, 34.3, 30.0, 25.2, 19.7. HRMS (ESI) calculated for C_8_H_16_SO_4_Na^+^ [M + Na]^+^ 231.0662, found 231.0661.

#### 3.2.6. Synthesis of (3R,5S)-2,2,5-trimethyloct-7-en-3-ol (S12)

The product **S12** was synthesised according to the procedures for the synthesis of **S11** from **7** (620 mg, 2.98 mmol, 1.0 eq.). Purification of the crude product was performed using flash chromatography on silica gel (hexanes/EtOAc = 20:1) to afford **S12** (370 mg, 73%) as a colorless oil. TLC: R*_f_* = 0.6 (hexanes/EtOAc = 10:1), iodine and PMA stain. $\alpha_{D}^{25}$= +24.9 (*c* 0.01, CHCl_3_). ^1^H NMR (400 MHz, CDCl_3_) δ 5.78 (ddt, *J* = 17.4, 10.4, 7.1 Hz, 1H), 5.13–4.81 (m, 2H), 3.28 (dd, *J* = 10.6, 1.8 Hz, 1H), 2.21–1.85 (m, 2H), 1.75 (dtt, *J* = 13.6, 6.7, 3.3 Hz, 1H), 1.35 (ddd, *J* = 13.9, 10.6, 3.2 Hz, 1H), 1.18 (ddd, *J* = 14.0, 10.6, 1.8 Hz, 1H), 0.89 (d, *J* = 6.5 Hz, 3H), 0.87 (s, 9H). ^13^C NMR (100 MHz, CDCl_3_) δ 137.6, 115.9, 77.4, 42.8, 38.5, 35.0, 29.7, 25.8, 18.9. HRMS (ESI) calculated for C_11_H_22_ONa^+^ [M + Na]^+^ 193.1563, found 193.1562.

#### 3.2.7. Synthesis of tert-butyldimethyl(((3R,5S)-2,2,5-trimethyloct-7-en-3-yl)oxy)silane (13)

The product **13** was synthesised according to the procedures for the synthesis of **10** from **S12** (2.9 g, 17.14 mmol, 1.0 eq.). Purification of the crude product was performed using flash chromatography on silica gel (hexanes) to afford **13** (4.67 g, 96%) as a colorless oil. TLC: R*_f_* = 0.95 (hexanes), iodine and PMA stain. $\alpha_{D}^{27}$ = +11.1 (*c* 0.01, CHCl_3_). ^1^H NMR (400 MHz, CDCl_3_) δ 5.98–5.61 (m, 1H), 5.19–4.90 (m, 2H), 3.30 (dd, *J* = 8.5, 1.8 Hz, 1H), 2.12–1.97 (m, 1H), 1.96–1.84 (m, 1H), 1.75–1.62 (m, 1H), 1.38 (ddd, *J* = 14.1, 8.5, 2.9 Hz, 1H), 1.19 (ddd, *J* = 14.0, 10.9, 1.8 Hz, 1H), 0.89 (s, 8H), 0.84 (d, *J* = 6.5 Hz, 3H), 0.84 (s, 9H), 0.04 (s, 6H). ^13^C NMR (100 MHz, CDCl_3_) δ 137.7, 115.7, 78.6, 43.1, 40.6, 35.8, 29.6, 26.6, 26.4, 19.2, 18.7, −3.2, −3.5. HRMS (ESI) calculated for C_17_H_36_OSi Na^+^ [M + Na]^+^ 307.2428, found 307.2423.

#### 3.2.8. Synthesis of (4S,6R)-6-((tert-butyldimethylsilyl)oxy)-4,7,7-trimethyloctan-1-ol (14)

The product **14** was synthesised according to the procedures for the synthesis of **11** from **13** (2.58 g, 9.08 mmol, 1.0 eq.). Purification of the crude product was performed using flash chromatography on silica gel (hexanes/EtOAc = 10:1) to afford alcohol **14** (2.33 g, 85%) as a colorless oil. TLC: R*_f_* = 0.5 (hexanes/EtOAc = 4:1), PMA stain. $\alpha_{D}^{28}$ = +11.1 (*c* 0.01, CHCl_3_). ^1^H NMR (400 MHz, CDCl_3_) δ 3.62 (td, *J* = 6.8, 1.1 Hz, 2H), 3.29 (dd, *J* = 8.6, 1.7 Hz, 1H), 1.65–1.48 (m, 4H), 1.36 (ddd, *J* = 14.0, 8.6, 2.8 Hz, 1H), 1.25 (ddd, *J* = 7.3, 6.2, 2.5 Hz, 1H), 1.25–1.12 (m, 2H), 0.89 (s, 9H), 0.84 (d, *J* = 7.3 Hz, 3H), 0.83 (s, 9H), 0.04 (s, 3H), 0.03 (s, 3H). ^13^C NMR (100 MHz, CDCl_3_) δ 78.6, 63.5, 41.0, 35.7, 34.7, 30.5, 29.3, 26.6, 26.4, 19.4, 18.7, −3.1, −3.5. HRMS (ESI) calculated for C_17_H_38_O_2_SiNa^+^ [M + Na]^+^ 325.2533, found 325.2534.

#### 3.2.9. Synthesis of (4S,6R)-6-((tert-butyldimethylsilyl)oxy)-4,7,7-trimethyloctanal (15)

The product **15** was synthesised according to the procedures for the synthesis of **12** from **14** (2.0 g, 6.6 mmol, 1.0 eq.). Purification of the crude product was performed using flash chromatography on silica gel (hexanes/EtOAc = 20:1) to afford aldehyde **15** (1.68 g, 85%) as a colorless oil. TLC: R*_f_* = 0.6 (hexanes/EtOAc = 10:1), PMA stain. $\alpha_{D}^{25}$ = +12.7 (*c* 0.01, CHCl_3_). ^1^H NMR (400 MHz, CDCl_3_) δ 9.76 (t, *J* = 1.8 Hz, 1H), 3.29 (dd, *J* = 8.6, 1.7 Hz, 1H), 2.51–2.30 (m, 2H), 1.68–1.43 (m, 3H), 1.35 (ddd, *J* = 13.9, 8.6, 2.5 Hz, 1H), 1.20 (ddd, *J* = 14.0, 10.4, 1.8 Hz, 1H), 0.88 (s, 9H), 0.85 (d, *J* = 6.1 Hz, 3H), 0.83 (s, 9H), 0.03 (s, 3H), 0.02 (s, 3H). ^13^C NMR (100 MHz, CDCl_3_) δ 202.9, 78.4, 41.9, 40.7, 35.7, 30.5, 29.2, 26.5, 26.4, 19.2, 18.7, −3.2, −3.5. HRMS (ESI) calculated for C_17_H_38_O_2_SiNa^+^ [M + Na]^+^ 323.2377, found 323.2376.

#### 3.2.10. Synthesis of (6S,11R,13R,Z)-13-(tert-butyl)-2,2,3,3,6,11,15,15,16,16-decamethyl-4,14-dioxa-3,15-disilaheptadec-7-ene (16)

To a cooled (−78 °C) stirring solution of sulfone **8 [44]** (200 mg, 0.52 mmol, 1.2 eq.) in dry toluene (4 mL, 0.1 M), NaHMDS (2 M in THF, 0.26 mL, 0.52 mmol, 1.2 eq.) was added dropwise for 1 h, followed by a solution of aldehyde **12** (130 mg, 0.43 mmol, 1.0 eq.) in dry toluene (2 mL, 0.22 M). The reaction mixture was stirred at −78 °C for 3 h and then quenched with a saturated aqueous solution of NH_4_Cl (10 mL). The layers were separated and the aqueous layer was extracted with MTBE (3 × 15 mL). The combined organic layers were dried over anhydrous sodium sulfate, filtered and concentrated under reduced pressure. Purification of the crude product was performed using flash chromatography on silica gel (hexanes/EtOAc = 100:1) to afford olefin **16** (149 mg, 74%) as a colorless oil. TLC: R*_f_* = 0.6 (hexanes/ EtOAc = 80:1), iodine and PMA stain. $\alpha_{D}^{24}$ = +15.8 (*c* 0.01, CHCl_3_). ^1^H NMR (400 MHz, CDCl_3_ as a mixture of *Z/E* = 3:1) δ (5.53–5.27 (m) and 5.14 (ddt, *J* = 11.0, 9.5, 1.6 Hz), 2H), 3.47 (ddd, *J* = 9.7, 6.0, 4.9 Hz, 1H), 3.35 (ddd, *J* = 9.8, 7.3, 5.3 Hz, 1H), 3.30 (dd, *J* = 7.1, 2.9 Hz, 1H), (2.69–2.54 (m) and 2.33–2.21 (m), 1H), 2.21–2.08 (m, 1H), 2.12–1.98 (m, 1H), 2.02–1.86 (m, 1H), 1.64–1.51 (m, 1H), 1.52–1.37 (m, 2H), 1.24–1.12 (m, 1H), 1.09–0.97 (m, 1H), (0.97 (d, *J* = 6.8 Hz) and 0.96 (d, *J* = 6.7 Hz), 3H), 0.92 (d, *J* = 6.6 Hz, 3H), 0.90 (m, 18H), 0.85 (s, 9H), 0.09 (m, 12H). ^13^C NMR (100 MHz, CDCl_3_) δ 132.8, 132.6, 130.5, 130.4, 78.7, 78.6, 68.5, 68.2, 41.9, 41.8, 39.5, 36.7, 36.3, 36.0, 35.0, 30.2, 30.0, 29.7, 26.5, 26.4, 26.2, 25.1, 20.9, 20.8, 18.7, 18.6, 17.7, 16.9, −3.2, −3.7, −5.1, −5.1. HRMS (ESI) calculated for C_27_H_58_O_2_Si_2_Na^+^ [M + Na]^+^ 493.3869, found 493.3869.

#### 3.2.11. Synthesis of (2S,7R,9R)-2,7,10,10-tetramethylundecane-1,9-diol (17)

To a solution of alkene **16** (149 mg, 0.317 mmol, 1.0 eq.) in anaerobic MeOH (10 mL, 0.03 M), PdCl_2_ (17 mg, 0.095 mmol, 0.3 eq.) was added under an argon atmosphere. The reaction flask was evacuated and purged with H_2_ three times and then the reaction was stirred at ambient temperature under a hydrogen atmosphere for 10 h. The reaction flask was then evacuated and purged with nitrogen three times. The catalyst was removed via filtration through Celite. The filter cake was rinsed thoroughly with MeOH and the filtrate was concentrated in vacuo to provide the crude product. Purification of the crude product was performed using flash chromatography on silica gel (hexanes/EtOAc = 3:1) to afford diol **17** (39 mg, 50%) as a colorless oil. TLC: R*_f_* = 0.3 (hexanes/ EtOAc = 3:1), PMA stain. $\alpha_{D}^{26}$ = +22.6 (*c* 0.01, CHCl_3_). ^1^H NMR (500 MHz, CDCl_3_) δ 3.48 (dd, *J* = 10.5, 5.8 Hz, 1H), 3.42 (dd, *J* = 10.5, 6.3 Hz, 1H), 3.28 (dd, *J* = 10.2, 1.8 Hz, 1H), 1.65–1.57 (m, 3H), 1.45–1.39 (m, 2H), 1.38–1.33 (m, 3H), 1.25–1.19 (m, 3H), 1.18–1.14 (m, 1H), 1.11–1.05 (m, 1H), 1.03–0.99 (m, 1H), 0.92 (d, *J* = 6.7 Hz, 3H), 0.90 (d, *J* = 6.8 Hz, 3H) 0.87 (s, 9H). ^13^C NMR (125 MHz, CDCl_3_) δ 77.7, 68.4, 39.4, 35.9, 35.4, 35.1, 33.2, 29.9, 27.4, 27.1, 25.8, 21.2, 16.8. HRMS (ESI) calculated for C_15_H_32_O_2_Na^+^ [M + Na]^+^ 267.2295, found 267.2293.

#### 3.2.12. Synthesis of (2S,7R,9R)-9-hydroxy-2,7,10,10-tetramethylundecanoic acid (S14)

To a solution of diol **17** (53 mg, 0.217 mmol, 1.0 eq.) in DCM (3 mL, 0.07 M), TEMPO (7 mg, 0.043 mmol, 0.2 eq.), H_2_O (0.2 mL, 11.0 mmol, 50 eq.) and PhI(OAc)_2_ (175 mg, 0.54 mmol, 2.5 eq.) were sequentially added and stirred at room temperature for 20 h before it was quenched with saturated aqueous solution of Na_2_S_2_O_3_ (3 mL) and extracted with DCM (3 × 10 mL). The combined organic layers were washed with brine (10 mL), dried over anhydrous sodium sulfate, filtered and concentrated under reduced pressure. Purification of the crude product was performed using flash chromatography on silica gel (hexanes/EtOAc = 10:1–5:1) to afford the corresponding acid **S14** (51 mg, 90%) as a colorless oil. **TLC**: R*_f_* = 0.4 (hexanes/EtOAc = 3:1), PMA stain. $\alpha_{D}^{24}$ = +20.6 (*c* 0.01, CHCl_3_). ^1^H NMR δ 3.29 (dd, *J* = 10.3, 1.8 Hz, 1H), 2.52–2.36 (m, 1H), 1.77–1.64 (m, 1H), 1.67–1.57 (m, 1H), 1.49–1.37 (m, 2H), 1.40–1.27 (m, 4H), 1.25–1.13 (m, 2H), 1.16 (d, *J* = 7.0 Hz, 3H), 1.08–0.95 (m, 1H), 0.91 (d, *J* = 6.7 Hz, 3H), 0.87 (s, 9H). ^13^C NMR (100 MHz, CDCl_3_) δ 182.7, 77.8, 39.5, 39.3, 35.2, 35.0, 33.6, 29.8, 27.5, 26.7, 25.7, 21.1, 17.1. HRMS (ESI) calculated for C_15_H_30_O_3_Na^+^ [M + Na]^+^ 281.2087, found 281.2091.

#### 3.2.13. Synthesis of (2S,7R,9R)-N-allyl-9-hydroxy-N,2,7,10,10-pentamethylundecanamide (18)

To a solution of acid **S14** (39 mg, 0.15 mmol, 1.0 eq.) and *N*-allylmethylamine (29 μL, 0.30 mmol, 2.0 eq.) in dry DCM (2 mL, 0.08 M), HOAt (41 mg, 0.30 mmol, 2.0 eq.), DMAP (56 mg, 0.46 mmol, 3.0 eq.) and EDCI (58 mg, 0.30 mmol, 2.0 eq.) were sequentially added at 0 °C. The reaction mixture was allowed to stir at room temperature for 12 h, quenched with saturated aqueous solution of NaHCO_3_ (5 mL) and extracted with DCM (3 × 5 mL). The combined organic layers were washed with brine (5 mL), dried over anhydrous sodium sulfate, filtered and concentrated under reduced pressure. Purification of the crude product was performed using flash chromatography on silica gel (hexanes/EtOAc = 3:1) to afford the amide **18** (32 mg, 68%) as a colorless oil. TLC: R*_f_* = 0.4 (hexanes/EtOAc = 2:1), iodine and PMA stain. $\alpha_{D}^{26}$ = +26.0 (*c* 0.01, CHCl_3_). ^1^H NMR (400 MHz, CDCl_3_ as a 1:1 mixture of two major conformers) δ 5.82–5.66 (m, 1H), 5.24–5.04 (m, 2H), 4.07–3.94 (m, 1H), 3.94–3.90 (m, 1H), 3.34–3.20 (m, 1H), (2.97 and 2.91 (s), 3H), 2.74–2.52 (m, 1H), 1.87 (s, 1H), 1.78–1.66 (m, 1H), 1.65–1.55 (m, 1H), 1.51–1.40 (m, 1H), 1.36–1.32 (m, 1H), 1.32–1.29 (m, 1H), 1.29–1.26 (m, 1H), 1.26–1.21 (m, 2H), 1.21–1.19 (m, 1H), 1.18–1.12 (m, 1H), (1.09 and 1.08 (d, *J* = 6.7 Hz), 3H), 1.04–0.92 (m, 1H), (0.89 and 0.89 (d, *J* = 6.7 Hz), 3H), 0.87 (s, 9H). ^13^C NMR (100 MHz, CDCl_3_ as a 1:1 mixture of two major conformers) δ 177.3 and 176.6, 133.4 and 133.1, 117.0 and 116.6, 77.4, 52.2 and 50.3, 39.5 and 39.5, 36.0 and 35.8, 35.1 and 35.0, 34.9 and 34.8, 34.4, 34.1 and 34.0, 29.5 and 29.5, 27.7 and 27.7, 26.8 and 26.8, 25.8 and 25.8, 21.2 and 21.2, 18.4 and 17.8. HRMS (ESI) calculated for C_19_H_37_NO_2_Na^+^ [M + Na]^+^ 334.2717, found 334.2717.

#### 3.2.14. Synthesis of (3R,5R,10S)-11-(allyl(methyl)amino)-2,2,5,10-tetramethyl-11-oxoundecan-3-yl acrylate (19)

To a solution of alcohol **18** (32 mg, 0.1 mmol, 1.0 eq.) in dry DCM (2 mL, 0.05 M), Et_3_N (69 μL, 0.5 mmol, 5.0 eq.), DMAP (2.4 mg, 0.02 mmol, 0.2 eq.) and acryloyl chloride (24 μL, 0.3 mmol, 3.0 eq.) were sequentially added at 0 °C. The reaction mixture was allowed to stir at room temperature for 12 h, quenched with water (1 mL) and extracted with DCM (3 × 3 mL). The combined organic layers were washed with brine (2 mL), dried over anhydrous sodium sulfate, filtered and concentrated under reduced pressure. Purification of the crude product was performed using flash chromatography on silica gel (hexanes/EtOAc = 5:1) to afford ester **19** (33 mg, 90%) as a colorless oil. TLC: R*_f_* = 0.4 (hexanes/EtOAc = 3:1), iodine and PMA stain. $\alpha_{D}^{26}$ = +23.6 (*c* 0.01, CHCl_3_). ^1^H NMR (400 MHz, CDCl_3_ as a 1:1 mixture of two major conformers) δ 6.37 (dd, *J* = 17.3, 1.6 Hz, 1H), 6.11 (dd, *J* = 17.3, 10.4 Hz, 1H), 5.80 (dd, *J* = 10.4, 1.6 Hz, 1H), 5.80–5.66 (m, 1H), 5.26–5.04 (m, 2H), 4.88 (ddd, *J* = 7.9, 4.3, 1.1 Hz, 1H), 4.06–3.95 (m, 1H), 3.98–3.89 (m, 1H), (2.97 and 2.92 (s, 3H)), 2.75–2.52 (m, 1H), 1.71–1.58 (m, 1H), 1.51–1.36 (m, 3H), 1.37–1.23 (m, 3H), 1.27–1.10 (m, 3H), (1.09 and 1.08 (d, *J* = 6.8 Hz, 3H)), 1.06–0.94 (m, 1H), 0.88 (s, 9H), (0.85 and 0.84 (d, *J* = 6.5 Hz, 3H)). ^13^C NMR (100 MHz, CDCl_3_ as a 1:1 mixture of two major conformers) δ 177.4 and 176.7, 166.2, 133.5 and 133.2, 130.3, 129.1, 117.0 and 116.6, 79.2, 52.2 and 50.2, 37.3, 35.9 and 35.7, 35.7, 35.0, 34.8 and 34.7, 34.4 and 34.0, 29.8, 28.1 and 28.1, 27.0 and 27.0, 26.0, 20.9, 18.24 and 17.6. HRMS (ESI) calculated for C_22_H_39_NO_3_Na^+^ [M + Na]^+^ 388.2822, found 388.2822.

#### 3.2.15. Synthesis of (2S,7R,9R)-laingolide A (1a)

To a solution of diene **19** (33 mg, 0.09 mmol, 1.0 eq.) in DCE (90 mL, 0.001 M) at room temperature, second-generation Grubbs catalyst (**G-II**) (7.6 mg, 0.009 mmol, 0.1 eq.) was added. The reaction mixture was heated at 80 °C for 24 h and then a second portion of **G-II** (7.6 mg, 0.009 mmol, 0.1 eq.) was added. The reaction mixture was kept at 80 °C, stirred for another 24 h and then concentrated under reduced pressure. Purification of the crude product was performed using flash chromatography on silica gel (hexanes/EtOAc = 5:1) to afford an inseparable mixture of the desired product **S15** and a minor unidentified byproduct as a white solid.

To a solution of the above mixture of **S15** and a minor unidentified byproduct in degassed dry toluene (1 mL) under argon, a solution of RuH(PPh_3_)_3_(CO)Cl (8.6 mg, 0.009 mmol, 0.1 eq.) was added in degassed dry toluene (7 mL). The reaction mixture was heated to reflux for 24 h, cooled to room temperature, then concentrated under reduced pressure to provide the crude product. Purification of the crude product was performed using flash chromatography on silica gel (hexanes/EtOAc = 5:1) to afford (2S,7R,9R)-laingolide A (**1a**) (13.6 mg, 45% for two steps) as a white solid. TLC: R*_f_* = 0.6 (hexanes/ EtOAc = 2:1), UV and PMA stain. $\alpha_{D}^{26}$ = +301 (*c* 0.01, MeOH). ^1^H NMR (400 MHz, CDCl_3_) δ 6.76 (d, *J* = 13.8 Hz, 1H), 5.21 (ddd, *J* = 13.8, 9.4, 6.0 Hz, 1H), 4.94 (dd, *J* = 11.1, 2.5 Hz, 1H), 3.17–3.08 (m, 1H), 3.11 (s, 3H), 2.98 (ddd, *J* = 16.0, 9.5, 0.8 Hz, 1H), 2.86–2.72 (m, 1H), 1.80–1.63 (m, 2H), 1.59–1.52 (m, 1H), 1.46–1.40 (m, 1H), 1.40–1.33 (m, 2H), 1.32–1.27 (m, 2H), 1.25–1.17 (m, 2H), 1.15 (d, *J* = 6.5 Hz, 3H), 0.96–0.91 (m, 1H), 0.89 (d, *J* = 6.8 Hz, 3H), 0.88 (s, 9H). ^13^C NMR (100 MHz, CDCl_3_) δ 176.3, 173.3, 133.4, 104.2, 77.9, 37.7, 37.1, 35.7, 35.4, 34.3, 34.2, 31.3, 30.5, 27.6, 27.4, 26.7, 20.1, 17.2. HRMS (ESI) calculated for C_20_H_35_NO_3_Na^+^ [M + Na]^+^ 360.2509, found 360.2510.

#### 3.2.16. Synthesis of (6R,11R,13R,Z)-13-(tert-butyl)-2,2,3,3,6,11,15,15,16,16-decamethyl-4,14-dioxa-3,15-disilaheptadec-7-ene (20)

Product **20** was synthesised according to the procedures for the synthesis of **16** from **12** (910 mg, 3.0 mmol, 1.0 eq.) and sulfone *ent*-8 (1.4 g, 3.6 mmol, 1.2 eq.). Purification of the crude product was performed using flash chromatography on silica gel (hexanes/EtOAc = 100:1) to afford **20** (998 mg, 70%) as a colorless oil. TLC: R*_f_* = 0.6 (hexanes/EtOAc = 80:1), iodine and PMA stain. $\alpha_{D}^{26}$ = +1.10 (*c* 0.01, CHCl_3_). ^1^H NMR (400 MHz, CDCl_3_ as a mixture of *Z/E* = 3:1) δ (5.50–5.26 (m) and 5.14 (ddd, *J* = 10.9, 9.4, 1.5 Hz) 2H), 3.55–3.42 (m, 1H), 3.41–3.31 (m, 1H), 3.34–3.27 (m, 1H), (2.78–2.54 (m) and 2.37–2.20 (m), 1H), 2.22–2.03 (m, 1H), 2.07–1.94 (m, 1H), 1.69–1.51 (m, 1H), 1.51–1.37 (m, 2H), 1.26–1.13 (m, 1H), 1.10–0.98 (m, 1H), (0.96 (d, *J* = 6.7 Hz) and 0.96 (d, *J* = 6.7 Hz), 3H), 0.95–0.86 (m, 21H), 0.88–0.83 (m, 9H), 0.07–0.03 (m, 12H). ^13^C NMR (100 MHz, CDCl_3_) δ 132.8, 132.6, 130.5, 130.4, 78.7, 78.6, 68.5, 68.2, 41.9, 39.5, 36.7, 36.4, 36.0, 36.0, 35.0, 30.3, 30.0, 29.8, 26.6, 26.4, 26.2, 26.2, 25.2, 20.9, 18.7, 18.7, 18.6, 18.5, 17.7, 16.9, −3.2, −3.2, −3.7, −5.1, −5.1. HRMS (ESI) calculated for C_27_H_58_O_2_Si_2_Na^+^ [M + Na]^+^ 493.3869, found 493.3866.

#### 3.2.17. Synthesis of (2R,7R,9R)-2,7,10,10-tetramethylundecane-1,9-diol (21)

Product **21** was synthesised according to the procedures for the synthesis of **17** from **20** (250 mg, 0.53 mmol, 1.0 eq.). Purification of the crude product was performed using flash chromatography on silica gel (hexanes/EtOAc = 3:1) to afford **21** (59.5 mg, 46%) as a colorless oil. TLC: R*_f_* = 0.3 (hexanes/EtOAc = 3:1), PMA stain. $\alpha_{D}^{27}$ = +27.7 (*c* 0.01, CHCl_3_). ^1^H NMR (500 MHz, CDCl_3_) δ 3.47 (dd, *J* = 10.4, 5.9 Hz, 1H), 3.39 (dd, *J* = 10.5, 6.5 Hz, 1H), 3.26 (dd, *J* = 10.3, 1.8 Hz, 1H), 1.80 (s, 2H), 1.72–1.51 (m, 2H), 1.47–1.35 (m, 2H), 1.37–1.21 (m, 5H), 1.21–1.06 (m, 2H), 1.01 (ddd, *J* = 18.2, 9.5, 3.4 Hz, 1H), 0.91 (d, *J* = 6.7 Hz, 3H), 0.89 (d, *J* = 6.7 Hz, 3H), 0.86 (s, 9H). ^13^C NMR (125 MHz, CDCl_3_) δ 77.7, 68.4, 39.5, 35.8, 35.5, 35.0, 33.2, 30.0, 27.2, 27.1, 25.8, 21.1, 16.6. HRMS (ESI) calculated for C_15_H_32_O_2_Na^+^ [M + Na]^+^ 267.2295, found 267.2293.

#### 3.2.18. Synthesis of (2R,7R,9R)-9-hydroxy-2,7,10,10-tetramethylundecanoic acid (S16)

Product **S16** was synthesised according to the procedures for the synthesis of **S14** from **21** (258 mg, 1.06 mmol, 1.0 eq.). Purification of the crude product was performed using flash chromatography on silica gel (hexanes/EtOAc = 10:1–5:1) to afford **S16** (246 mg, 90%) as a colorless oil. TLC: R*_f_* = 0.4 (hexanes/EtOAc = 3:1), PMA stain. $\alpha_{D}^{27}$ = +17.7 (*c* 0.01, CHCl_3_). ^1^H NMR (400 MHz, CDCl_3_) δ 3.28 (dd, *J* = 10.3, 1.8 Hz, 1H), 2.53–2.32 (m, 1H), 1.82–1.56 (m, 2H), 1.49–1.37 (m, 2H), 1.39–1.17 (m, 5H), 1.21–1.13 (m, 1H), 1.16 (d, *J* = 6.9 Hz, 3H), 1.09–0.95 (m, 1H), 0.91 (d, *J* = 6.7 Hz, 3H), 0.87 (s, 9H). ^13^C NMR (100 MHz, CDCl_3_) δ 182.8, 77.9, 39.5, 39.3, 35.4, 35.0, 33.7, 30.0, 27.6, 26.8, 25.8, 21.1, 16.9. HRMS (ESI) calculated for C_15_H_30_O_3_Na^+^ [M + Na]^+^ 281.2087, found 281.2088.

#### 3.2.19. Synthesis of (2R,7R,9R)-N-allyl-9-hydroxy-N,2,7,10,10-pentamethylundecanamide (22)

Product **22** was synthesised according to the procedures for the synthesis of **18** from **S16** (105 mg, 0.4 mmol, 1.0 eq.). Purification of the crude product was performed using flash chromatography on silica gel (hexanes/EtOAc = 3:1) to afford **22** (83.4 mg, 67%) as a colorless oil. TLC: R*_f_* = 0.4 (hexanes/EtOAc = 2:1), iodine and PMA stain. $\alpha_{D}^{27}$ = +10.6 (*c* 0.01, CHCl_3_). ^1^H NMR (400 MHz, CDCl_3_ as a 1:1 mixture of two major conformers) δ 6.04–5.59 (m, 1H), 5.26–4.94 (m, 2H), 4.14–3.94 (m, 1H), 3.97–3.88 (m, 1H), 3.26 (dd, *J* = 10.2, 1.7 Hz, 1H), (2.97 and 2.92 (s, 3H)), 2.79–2.48 (m, 1H), 1.80–1.55 (m, 3H), 1.44–1.28 (m, 3H), 1.29–1.25 (m, 1H), 1.24 (s, 1H), 1.22–1.12 (m, 3H), (1.10 and 1.08 (d, *J* = 6.7 Hz, 3H)), 1.05–0.95 (m, 1H), (0.91 and 0.90 (d, *J* = 6.8 Hz, 3H)), 0.87 (s, 9H). ^13^C NMR (100 MHz, CDCl_3_ as a 1:1 mixture of two major conformers) δ 177.3 and 176.7, 133.4 and 133.2, 117.0 and 116.6, 77.7, 52.1 and 50.2, 39.4, 35.9 and 35.7, 35.6, 35.0, 34.8 and 34.5, 34.2 and 34.0, 30.1 and 30.1, 28.1 and 28.1, 27.1 and 27.0, 25.8 and 25.8, 21.2, 18.2 and 17.6. HRMS (ESI) calculated for C_19_H_37_NO_2_Na^+^ [M + Na]^+^ 334.2717, found 334.2718.

#### 3.2.20. Synthesis of (3R,5R,10R)-11-(allyl(methyl)amino)-2,2,5,10-tetramethylundecan-3-yl acrylate (23)

Product **23** was synthesised according to the procedures for the synthesis of **19** from **22** (63 mg, 0.2 mmol, 1.0 eq.). Purification of the crude product was performed using flash chromatography on silica gel (hexanes/EtOAc = 5:1) to afford **23** (64.3 mg, 88%) as a colorless oil. TLC: R*_f_* = 0.4 (hexanes/EtOAc = 3:1), iodine and PMA stain. $\alpha_{D}^{27}$ = +8.9 (*c* 0.01, CHCl_3_). ^1^H NMR (400 MHz, CDCl_3_ as a 1:1 mixture of two major conformers) δ 6.37 (dd, *J* = 17.3, 1.6 Hz, 1H), 6.11 (dd, *J* = 17.3, 10.4 Hz, 1H), 5.80 (dd, *J* = 10.4, 1.6 Hz, 1H), 5.79–5.66 (m, 1H), 5.27–5.04 (m, 2H), 4.94–4.82 (m, 1H), 4.06–3.95 (m, 1H), 3.97–3.84 (m, 1H), (2.97 and 2.91 (s, 3H)), 2.76–2.52 (m, 1H), 1.71–1.58 (m, 1H), 1.41 (ddd, *J* = 7.2, 5.6, 1.9 Hz, 2H), 1.39–1.26 (m, 1H), 1.27–1.16 (m, 5H), (1.10 and 1.08 (d, *J* = 6.7 Hz, 1H)), 1.06–0.95 (m, 1H), 0.88 (s, 9H), (0.85 and 0.84 (d, *J* = 6.5 Hz, 3H)). ^13^C NMR (100 MHz, CDCl_3_ as a 1:1 mixture of two major conformers) δ 177.4 and 176.7, 166.3, 133.5 and 133.2, 130.3, 129.1, 117.0 and 116.6, 79.2, 52.2 and 50.2, 37.2, 35.9 and 35.7, 35.5 and 35.0, 34.8 and 34.6, 34.3, 33.9, 29.7 and 29.7, 28.0 and 28.0, 26.8 and 26.8, 26.0, 20.9, 18.2 and 17.61. HRMS (ESI) calculated for C_22_H_39_NO_3_Na^+^ [M + Na]^+^ 388.2822, found 388.2822.

#### 3.2.21. Synthesis of (2R,7R,9R)-laingolide A (1b)

Product **S17** was synthesised according to the procedures for the synthesis of **S15** from **23** (10.3 mg, 0.027 mmol, 1.0 eq.). Purification of the crude product was performed using flash chromatography on silica gel (hexanes/EtOAc = 5:1) to afford an inseparable mixture of the desired product **S17** and a minor unidentified byproduct (9 mg) as a white solid.

(2*R*,7*R*,9*R*)-laingolide A (**1b**) was synthesised according to the procedures for the synthesis of (2*S*,7*S*,9*R*)-laingolide A (**1a**) from the above mixture of **S17** and a minor unidentified byproduct. Purification of the crude product was performed using flash chromatography on silica gel (hexanes/EtOAc = 5:1) to afford (2*R*,7*R*,9*R*)-laingolide A (**1b**) (4 mg, 44% for two steps) as a white solid. TLC: R*_f_* = 0.6 (hexanes/EtOAc = 2:1), UV and PMA stain. $\alpha_{D}^{23}$ = −76.3 (*c* 0.005, CHCl_3_). ^1^H NMR (500 MHz, CDCl_3_) δ 7.09 (d, *J* = 13.9 Hz, 1H), 5.16 (dt, *J* = 14.1, 7.2 Hz, 1H), 4.92 (dd, *J* = 11.9, 1.8 Hz, 1H), 3.09 (s, 3H), 3.05 (ddd, *J* = 13.3, 7.4, 0.9 Hz, 1H), 2.99 (ddd, *J* = 13.3, 6.9, 1.4 Hz, 1H), 2.91 (dt, *J* = 13.4, 6.5 Hz, 1H), 1.65 – 1.61 (m, 0H), 1.58 – 1.51 (m, 2H), 1.46 – 1.36 (m, 1H), 1.33 – 1.25 (m, 3H), 1.25 – 1.18 (m, 2H), 1.15 (d, *J* = 6.6 Hz, 3H), 1.10 – 0.99 (m, 1H), 0.88 (s, 9H), 0.85 – 0.79 (m, 1H), 0.78 (d, *J* = 6.4 Hz, 3H). ^13^C NMR (125 MHz, CDCl_3_) δ 176.5, 172.1, 133.8, 103.6, 78.5, 38.6, 37.3, 35.7, 35.4, 35.3, 34.1, 31.4, 26.7, 26.7, 26.6, 26.1, 20.9, 18.0. HRMS (ESI) calculated for C_20_H_35_NO_3_Na^+^ [M + Na]^+^ 360.2509, found 360.2510.

#### 3.2.22. Synthesis of (6S,11S,13R,Z)-13-(tert-butyl)-2,2,3,3,6,11,15,15,16,16-decamethyl-4,14-dioxa-3,15-disilaheptadec-7-ene (24)

Product **24** was synthesised according to the procedures for the synthesis of **16** from 1**5** (780 mg, 2.6 mmol, 1.0 eq.) and sulfone **8** (1.2 g, 3.1 mmol, 1.2 eq.). Purification of the crude product was performed using flash chromatography on silica gel (hexanes/EtOAc = 100:1) to afford **24** (831 mg, 68%) as a colorless oil. TLC: R*_f_* = 0.6 (hexanes/EtOAc = 80:1), iodine and PMA stain. $\alpha_{D}^{27}$ = +18.8 (*c* 0.01, CHCl_3_). ^1^H NMR (400 MHz, CDCl_3_ as a mixture of *Z*/*E* = 7:1) δ (5.45–5.28 (m) and 5.12 (ddt, *J* = 10.9, 9.4, 1.6 Hz), 2H), 3.46 (dd, *J* = 9.7, 5.9 Hz, 1H), 3.35 (dd, *J* = 9.8, 7.4 Hz, 1H), 3.31 (dd, *J* = 8.4, 1.7 Hz, 2H), (2.72–2.57 (m) and 2.33–2.21 (m), 1H), 2.17–1.96 (m, 2H), 1.68–1.54 (m, 1H), 1.41–1.25 (m, 2H), 1.29–1.15 (m, 2H), 0.96 (d, *J* = 6.8 Hz, 3H), 0.91 (s, 18H), 0.85 (d, *J* = 6.2 Hz, 12H), 0.05 (s, 12H). ^13^C NMR (100 MHz, CDCl_3_) δ 132.5, 130.6, 78.7, 68.2, 41.2, 39.0, 35.8, 35.0, 29.4, 26.6, 26.5, 26.2, 25.4, 19.1, 17.7, −3.1, −3.5, −5.1, −5.1. HRMS (ESI) calculated for C_27_H_58_O_2_Si_2_Na^+^ [M + Na]^+^ 493.3869, found 493.3868.

#### 3.2.23. Synthesis of (2S,7S,9R)-2,7,10,10-tetramethylundecane-1,9-diol (25)

Product **25** was synthesised according to the procedures for the synthesis of **17** from **24** (235 mg, 0.5 mmol, 1.0 eq.). Purification of the crude product was performed using flash chromatography on silica gel (hexanes/EtOAc = 3:1) to afford **25** (55 mg, 45%) as a colorless oil. TLC: R*_f_* = 0.3 (hexanes/EtOAc = 3:1), PMA stain. $\alpha_{D}^{25}$ = +14.4 (*c* 0.01, CHCl_3_). ^1^H NMR (400 MHz, CDCl_3_) δ 3.48 (dd, *J* = 10.6, 5.8 Hz, 1H), 3.39 (ddd, *J* = 10.3, 6.5, 0.8 Hz, 1H), 3.27 (dd, *J* = 10.6, 1.8 Hz, 1H), 1.74–1.53 (m, 4H), 1.46–1.33 (m, 2H), 1.32–1.25 (m, 4H), 1.24–1.08 (m, 4H), 0.89 (d, *J* = 6.7 Hz, 3H), 0.86 (d, *J* = 7.1 Hz, 3H), 0.87 (s, 9H). ^13^C NMR (100 MHz, CDCl_3_) δ 77.4, 68.4, 39.1, 38.5, 35.8, 34.9, 33.2, 29.7, 27.4, 27.3, 25.8, 19.0, 16.7. HRMS (ESI) calculated for C_15_H_32_O_2_Na^+^ [M + Na]^+^ 267.2295, found 267.2293.

#### 3.2.24. Synthesis of (2S,7S,9R)-9-hydroxy-2,7,10,10-tetramethylundecanoic acid (S18)

Product **S18** was synthesised according to the procedures for the synthesis of **S14** from **25** (50 mg, 0.21 mmol, 1.0 eq.). Purification of the crude product was performed using flash chromatography on silica gel (hexanes/EtOAc = 10:1–5:1) to afford **S18** (47.8 mg, 91%) as a colorless oil. TLC: R*_f_* = 0.4 (hexanes/EtOAc = 3:1), PMA stain. $\alpha_{D}^{27}$ = +13.8 (*c* 0.01, CHCl_3_). ^1^H NMR (500 MHz, CDCl_3_) δ 3.29 (dd, *J* = 10.6, 1.7 Hz, 1H), 2.52–2.35 (m, 1H), 1.72–1.64 (m, 1H), 1.64–1.56 (m, 1H), 1.47–1.39 (m, 1H), 1.35–1.28 (m, 5H), 1.26–1.23 (m, 1H), 1.23–1.10 (m, 2H), 1.16 (d, *J* = 7.0 Hz, 3H), 0.87 (s, 9H), 0.86 (d, *J* = 6.6 Hz, 3H). ^13^C NMR (125 MHz, CDCl_3_) δ 182.6, 77.6, 39.5, 38.9, 38.3, 34.9, 33.7, 29.5, 27.4, 27.0, 25.8, 19.1, 17.0. HRMS (ESI) calculated for C_15_H_30_O_3_Na^+^ [M + Na]^+^ 281.2087, found 281.2087.

#### 3.2.25. Synthesis of (2S,7S,9R)-N-allyl-9-hydroxy-N,2,7,10,10-pentamethylundecanamide (26)

Product **26** was synthesised according to the procedures for the synthesis of **18** from **S18** (47.8 mg, 0.182 mmol, 1.0 eq.). Purification of the crude product was performed using flash chromatography on silica gel (hexanes/EtOAc = 3:1) to afford **26** (42.5 mg, 75%) as a colorless oil. TLC: R*_f_* = 0.4 (hexanes/EtOAc = 2:1), iodine and PMA stain. $\alpha_{D}^{25}$ = +16.7 (*c* 0.01, CHCl_3_). ^1^H NMR (500 MHz, CDCl_3_ as a 1:1 mixture of two major conformers) δ 5.82–5.65 (m, 1H), 5.30–4.98 (m, 2H), 4.07–3.92 (m, 1H), 3.91 (d, *J* = 4.7 Hz, 1H), 3.26 (d, *J* = 10.5 Hz, 1H), (2.96 and 2.90 (s, 3H)), 2.73–2.51 (m, 1H), 1.74–1.62 (m, 1H), 1.61–1.55 (m, 1H), 1.40–1.27 (m, 3H), 1.27–1.18 (m, 5H), 1.17–1.10 (m, 2H), (1.09 and1.07 (d, *J* = 6.9 Hz, 3H)), 0.86 (s, 9H), 0.84 (d, *J* = 7.1 Hz, 3H). ^13^C NMR (125 MHz, CDCl_3_ as a 1:1 mixture of two major conformers) δ 177.3 and 176.6, 133.4 and 133.2, 117.0 and 116.6, 77.3, 52.1 and 50.2, 39.0, 38.4, 35.9 and 35.7, 34.9 and 34.8, 34.5 and 34.2, 33.9, 29.7, 28.0 and 27.9, 27.3, 25.8, 19.0, 18.2 and 17.61. HRMS (ESI) calculated for C_19_H_37_NO_2_Na^+^ [M + Na]^+^ 334.2717, found 334.2721.

#### 3.2.26. Synthesis of (3R,5S,10S)-11-(allyl(methyl)amino)-2,2,5,10-tetramethyl-11-oxoundecan-3-yl acrylate (27)

Product **27** was synthesised according to the procedures for the synthesis of **19** from **26** (116 mg, 0.372 mmol, 1.0 eq.). Purification of the crude product was performed using flash chromatography on silica gel (hexanes/EtOAc = 5:1) to afford **27** (115 mg, 85%) as a colorless oil. TLC: R*_f_* = 0.4 (hexanes/EtOAc = 3:1), iodine and PMA stain. $\alpha_{D}^{26}$ = +8.8 (*c* 0.01, CHCl_3_). ^1^H NMR (400 MHz, CDCl_3_ as a 1:1 mixture of two major conformers) δ 6.37 (dd, *J* = 17.4, 1.5 Hz, 1H), 6.11 (dd, *J* = 17.3, 10.4 Hz, 1H), 5.80 (dd, *J* = 10.4, 1.7 Hz, 1H), 5.72 (ddt, *J* = 16.5, 11.5, 5.9 Hz, 1H), 5.24–5.02 (m, 2H), 4.89 (d, *J* = 10.9 Hz, 1H), 4.08–3.93 (m, 1H), 3.95–3.87 (m, 1H), (2.95 and 2.91 (s, 3H)), 2.72–2.46 (m, 1H), 1.74–1.1 (m, 1H), 1.58–1.52 (m, 1H), 1.36–1.29 (m, 1H), 1.27–1.12 (m, 8H), (1.08 and 1.06 (d, *J* = 6.5 Hz, 3H)), 0.86 (s, 12H). ^13^C NMR (100 MHz, CDCl_3_ as a 1:1 mixture of two major conformers) δ 177.2 and 176.6, 166.3, 133.4 and 133.1, 130.4, 129.0, 117 and 116.6, 79.0, 52.1 and 50.2, 38.1 and 37.1, 35.9 and 35.7, 34.8 and 34.8, 34.5, 34.2, 33.9, 29.5, 27.9 and 27.9, 27.2, 26.1, 19.3, 18.2 and 17.6. HRMS (ESI) calculated for C_22_H_39_NO_3_Na^+^ [M + Na]^+^ 388.2822, found 388.2824.

#### 3.2.27. Synthesis of (2S,7S,9R)-laingolide A (1c)

Product **S19** was synthesised according to the procedures for the synthesis of **S15** from **27** (28 mg, 0.077 mmol, 1.0 eq.). Purification of the crude product was performed using flash chromatography on silica gel (hexanes/EtOAc = 5:1) to afford an inseparable mixture of the desired product **S19** and a minor unidentified byproduct (20 mg) as a white solid.

(2*S*,7*S*,9*R*)-laingolide A (**1c**) was synthesised according to the procedures for the synthesis of (2*S*,7*R*,9*R*)-laingolide A (**1a**) from the above mixture of **S19** and a minor unidentified byproduct. Purification of the crude product was performed using flash chromatography on silica gel (hexanes/EtOAc = 5:1) to afford (2*S*,7*S*,9*R*)-laingolide A (**1c**) (13 mg, 50% for two steps) as a white solid. TLC: R*_f_* = 0.6 (hexanes/EtOAc = 2:1), UV and PMA stain. $\alpha_{D}^{26}$ = +145.6 (*c* 0.01, CHCl_3_). ^1^H NMR (500 MHz, CDCl_3_) δ 7.02 (dd, *J* = 13.7, 1.4 Hz, 1H), 5.18 (ddd, *J* = 13.6, 10.3, 5.7 Hz, 1H), 4.81 (dd, *J* = 11.3, 1.3 Hz, 1H), 3.10 (s, 3H), 3.07 (ddd, *J* = 12.3, 5.6, 1.5 Hz, 1H), 3.03–2.95 (m, 1H), 2.94 (dd, *J* = 12.3, 10.4 Hz, 1H), 1.64–1.57 (m, 1H), 1.60–1.52 (m, 1H), 1.47–1.34 (m, 1H), 1.35–1.27 (m, 1H), 1.30–1.17 (m, 4H), 1.15 (d, *J* = 6.5 Hz, 3H), 1.17–1.12 (m, 2H), 1.03–0.95 (m, 1H), 0.89 (s, 9H), 0.84 (d, *J* = 5.7 Hz, 3H). ^13^C NMR (125 MHz, CDCl_3_) δ 176.4, 172.5, 133.5, 105.0, 79.7, 37.6, 36.8, 36.6, 36.2, 35.5, 35.1, 31.2, 27.7, 26.8, 26.7, 26.2, 21.2, 18.5. HRMS (ESI) calculated for C_20_H_35_NO_3_Na^+^ [M + Na]^+^ 360.2509, found 360.2512.

#### 3.2.28. Synthesis of (6R,11S,13R,Z)-13-(tert-butyl)-2,2,3,3,6,11,15,15,16,16-decamethyl-4,14-dioxa-3,15-disilaheptadec-7-ene (28)

Product **28** was synthesised according to the procedures for the synthesis of **16** from **15** (700 mg, 2.34 mmol, 1.0 eq.) and sulfone ***ent*-8** (1.1 g, 2.8 mmol, 1.2 eq.). Purification of the crude product was performed using flash chromatography on silica gel (hexanes/EtOAc = 100:1) to afford **28** (847 mg, 77%) as a colorless oil. TLC: R*_f_* = 0.6 (hexanes/EtOAc = 80:1), iodine and PMA stain. $\alpha_{D}^{27}$ = −1.9 (*c* 0.01, CHCl_3_). ^1^H NMR (400 MHz, CDCl_3_ as a mixture of *Z*/*E* = 7:1) δ (5.50–5.26 (m), 5.11 (ddt, *J* = 11.0, 9.6, 1.6 Hz), 2H), 3.52–3.43 (m, 1H), 3.39–3.33 (m, 1H), 3.32–3.26 (m, 1H), (2.69–2.55 (m) and 2.32–2.20 (m), 1H), 2.14–1.90 (m, 2H), 1.69–1.49 (m, 1H), 1.38–1.32 (m, 1H), 1.31–1.24 (m, 1H), 1.26–1.12 (m, 2H), (0.97 (d, *J* = 6.8 Hz) and 0.95 (d, *J* = 6.7 Hz), 3H), 0.90 (s, 18H), 0.87–0.81 (m, 12H), 0.04 (m, 12H). ^13^C NMR (100 MHz, CDCl_3_) δ 132.5, 130.5, 78.6, 68.2, 41.1, 39.0, 35.8, 35.0, 29.3, 26.6, 26.4, 26.1, 25.4, 19.2, 18.7, 18.5, 17.7, −3.1, −3.5, −5.1, −5.1. HRMS (ESI) calculated for C_27_H_58_O_2_Si_2_Na^+^ [M + Na]^+^ 493.3869, found 493.3866.

#### 3.2.29. Synthesis of (2R,7S,9R)-2,7,10,10-tetramethylundecane-1,9-diol (29)

Product **29** was synthesised according to the procedures for the synthesis of **17** from **28** (250 mg, 0.53 mmol, 1.0 eq.). Purification of the crude product was performed using flash chromatography on silica gel (hexanes/EtOAc = 3:1) to afford **29** (60.8 mg, 47%) as a colorless oil. TLC: R*_f_* = 0.3 (hexanes/ EtOAc = 3:1), PMA stain. $\alpha_{D}^{26}$ = +19.1 (*c* 0.01, CHCl_3_). ^1^H NMR (400 MHz, CDCl_3_) δ 3.44 (ddd, *J* = 10.2, 5.8, 1.7 Hz, 1H), 3.35 (ddd, *J* = 10.5, 6.5, 1.9 Hz, 1H), 3.24 (dt, *J* = 10.6, 1.5 Hz, 1H), 2.14 (d, *J* = 31.1 Hz, 2H), 1.68–1.50 (m, 2H), 1.42–1.30 (m, 2H), 1.30–1.19 (m, 5H), 1.17–0.98 (m, 3H), 0.87 (d, *J* = 6.7 Hz, 3H), 0.85–0.82 (m, 12H). ^13^C NMR (100 MHz, CDCl_3_) δ 77.3, 68.2, 39.0, 38.4, 35.8, 34.9, 33.2, 29.6, 27.4, 27.3, 25.8, 19.0, 16.7. HRMS (ESI) calculated for C_15_H_32_O_2_Na^+^ [M + Na]^+^ 267.2295, found 267.2295.

#### 3.2.30. Synthesis of (2R,7S,9R)-9-hydroxy-2,7,10,10-tetramethylundecanoic acid (S20)

Product **S20** was synthesised according to the procedures for the synthesis of **S14** from **29** (112 mg, 0.46 mmol, 1.0 eq.). Purification of the crude product was performed using flash chromatography on silica gel (hexanes/EtOAc = 10:1–5:1) to afford **S20** (108 mg, 91%) as a colorless oil. TLC: R*_f_* = 0.4 (hexanes/EtOAc = 3:1), PMA stain. $\alpha_{D}^{27}$ = +8.4 (*c* 0.01, CHCl_3_). ^1^H NMR (500 MHz, CDCl_3_) δ 3.29 (dd, *J* = 10.6, 1.7 Hz, 1H), 2.51–2.37 (m, 1H), 1.78–1.57 (m, 2H), 1.46–1.38 (m, 1H), 1.36–1.28 (m, 5H), 1.28–1.23 (m, 1H), 1.22–1.18 (m, 1H), 1.16 (d, *J* = 6.9 Hz, 3H), 1.16–1.13 (m, 1H), 0.88 (s, 9H), 0.86 (d, *J* = 6.5 Hz, 3H). ^13^C NMR (125 MHz, CDCl_3_) δ 182.7, 77.6, 39.5, 39.0, 38.2, 34.9, 33.7, 29.6, 27.5, 27.0, 25.8, 19.0, 17.1. HRMS (ESI) calculated for C_15_H_30_O_3_Na^+^ [M + Na]^+^ 281.2087, found 281.2087.

#### 3.2.31. Synthesis of (2R,7S,9R)-N-allyl-9-hydroxy-N,2,7,10,10-pentamethylundecanamide (30)

Product **30** was synthesised according to the procedures for the synthesis of **18** from **S20** (116 mg, 0.45 mmol, 1.0 eq.). Purification of the crude product was performed using flash chromatography on silica gel (hexanes/EtOAc = 3:1) to afford **30** (105 mg, 75%) as a colorless oil. TLC: R*_f_* = 0.4 (hexanes/EtOAc = 2:1), iodine and PMA stain. $\alpha_{D}^{26}$ = +2.8 (*c* 0.01, CHCl_3_). ^1^H NMR (400 MHz, CDCl_3_ as a 1:1 mixture of two major conformers) δ 5.97–5.56 (m, 1H), 5.30–5.00 (m, 2H), 4.04–3.92 (m, 1H), 3.93 (dt, *J* = 4.9, 1.8 Hz, 1H), 3.27 (dt, *J* = 10.3, 1.4 Hz, 1H), (2.97 and 2.92 (s, 3H)), 2.79–2.43 (m, 1H), 1.79–1.53 (m, 3H), 1.38–1.23 (m, 6H), 1.21–1.13 (m, 3H), (1.10 and 1.08 (d, *J* = 6.8 Hz, 3H)), 0.87 (s, 9H), (0.85 and 0.85 (d, *J* = 6.5 Hz, 3H)). ^13^C NMR (100 MHz, CDCl_3_ as a 1:1 mixture of two major conformers) δ 177.3 and 176.6, 133.4 and 133.2, 117.0 and 116.6, 77.4, 52.2 and 50.2, 39.1, 38.4 and 38.3, 35.9 and 35.7, 34.9 and 34.8, 34.5 and 34.3, 33.9, 29.6, 27.9 and 27.9, 27.3, 25.8, 19.0, 18.3 and 17.7. HRMS (ESI) calculated for C_19_H_37_NO_2_Na^+^ [M + Na]^+^ 334.2717, found 334.2716.

#### 3.2.32. Synthesis of (3R,5S,10R)-11-(allyl(methyl)amino)-2,2,5,10-tetramethyl-11-oxoundecan-3-yl acrylate (31)

Product **31** was synthesised according to the procedures for the synthesis of **19** from **30** (88 mg, 0.28 mmol, 1.0 eq.). Purification of the crude product was performed using flash chromatography on silica gel (hexanes/EtOAc = 5:1) to afford **31** (88 mg, 86%) as a colorless oil. TLC: R*_f_* = 0.4 (hexanes/EtOAc = 3:1), iodine and PMA stain. $\alpha_{D}^{27}$ = +12.1 (*c* 0.01, CHCl_3_). ^1^H NMR (400 MHz, CDCl_3_ as a 1:1 mixture of two major conformers) δ 6.38 (dd, *J* = 17.3, 1.6 Hz, 1H), 6.12 (dd, *J* = 17.3, 10.4 Hz, 1H), 5.80 (dd, *J* = 10.3, 1.6 Hz, 1H), 5.78–5.66 (m, 1H), 5.33–5.04 (m, 2H), 4.90 (dd, *J* = 11.0, 1.2 Hz, 1H), 4.16–3.94 (m, 1H), 3.92 (dt, *J* = 4.9, 1.8 Hz, 1H), (2.96 and 2.91 (s, 3H)), 2.72–2.49 (m, 1H), 1.92–1.46 (m, 3H), 1.29–1.15 (m, 8H), (1.09 and 1.08 (d, *J* = 6.8 Hz, 3H)), 0.88 (s, 12H). ^13^C NMR (100 MHz, CDCl_3_ as a 1:1 mixture of two major conformers) δ 177.3 and 176.6, 166.4, 133.4 and 133.2, 130.4, 129.0, 117.0 and 116.6, 79.0, 52.1 and 50.2, 38.2, 37.1, 35.9 and 35.7, 34.9 and 34.8, 34.5 and 34.3, 33.9, 29.5, 28.0 and 27.9, 27.2, 26.1, 19.2, 18.2 and 17.6. HRMS (ESI) calculated for C_22_H_39_NO_3_Na^+^ [M + Na]^+^ 388.2822, found 388.2821.

#### 3.2.33. Synthesis of (2R,7S,9R)-laingolide A (1d)

Product **S21** was synthesised according to the procedures for the synthesis of **S15** from **31** (30.9 mg, 85 μmol, 1.0 eq.). Purification of the crude product was performed using flash chromatography on silica gel (hexanes/EtOAc = 5:1) to afford an inseparable mixture of the desired product **S21** and a minor unidentified byproduct (28 mg) as a white solid.

(2*R*,7*S*,9*R*)-laingolide A (**1d**) was synthesised according to the procedures for the synthesis of (2*S*,7*R*,9*R*)-laingolide A (**1a**) from the above mixture of the desired product **S21** and a minor unidentified byproduct. Purification of the crude product was performed using flash chromatography on silica gel (hexanes/EtOAc = 5:1) to afford (2*R*,7*S*,9*R*)-laingolide A (**1d**) (13.4 mg, 47% for two steps) as a white solid. TLC: R*_f_* = 0.6 (hexanes/EtOAc = 2:1), UV and PMA stain. $\alpha_{D}^{23}$ = −55.3 (*c* 0.01, CHCl_3_). ^1^H NMR (400 MHz, CDCl_3_) δ 6.74 (d, *J* = 13.9 Hz, 1H), 5.21 (ddd, *J* = 13.8, 8.6, 6.3 Hz, 1H), 4.86 (dd, *J* = 10.9, 1.3 Hz, 1H), 3.20 (ddd, *J* = 16.5, 6.3, 1.4 Hz, 1H), 3.11 (s, 3H), 3.07 (ddd, *J* = 16.5, 8.7, 1.0 Hz, 1H), 2.68 (dqd, *J* = 12.9, 6.6, 4.4 Hz, 1H), 1.63–1.50 (m, 1H), 1.49–1.33 (m, 3H), 1.29–1.25 (m, 1H), 1.25–1.23 (m, 2H), 1.22–1.16 (m, 2H), 1.14 (d, *J* = 6.4 Hz, 4H), 1.14–1.05 (m, 1H), 0.88 (s, 9H), 0.85 (d, *J* = 5.8 Hz, 3H). ^13^C NMR (100 MHz, CDCl_3_) δ 176.6, 171.9, 132.6, 106.8, 80.5, 38.7, 37.5, 36.2, 35.6, 35.0, 33.7, 32.2, 26.9, 26.6, 25.3, 24.9, 18.9, 16.6. HRMS (ESI) calculated for C_20_H_35_NO_3_Na^+^ [M + Na]^+^ 360.2509, found 360.2513.

## 4. Conclusions

In summary, we have unambiguously established the relative and absolute configuration of laingolide A through the total synthesis of four diastereomers of the natural product. The key features of the convergent and fully stereocontrolled route included a copper-catalysed stereospecific Kumada-type coupling, a Julia-Kocienski olefination and an RCM/alkene migration sequence to access the desired macrocyclic enamide.

## Data Availability

Not applicable.

## Supplementary Materials

The following are available online at https://www.mdpi.com/article/10.3390/md19050247/s1: initial approaches via the cross-metathesis and ^1^H- and ^13^C-NMR charts of all the compounds.
