# Supplementary material for: Total Synthesis and Structural Reassignment of Laingolide A
Source: Mar Drugs. 2021 Apr 27;19(5):247. doi: 10.3390/md19050247 (PMC8145716; doi:10.3390/md19050247)

Supplementary Information

Total Synthesis and Structural Reassignment of Laingolide A

Fusong Wu [1], Tao Zhang [1], Jie Yu [1], Yian Guo [1,2,*] and Tao Ye [1,*]

Table of Contents

1. Initial approach via cross metathesis 2
2. synthesis of ***ent*-8**..4
3. Comparison of ^{1}H NMR and ^{13}C NMR data of laingolide A 6
4. References..7
5. NMR spectra 8

1. Initial approach via cross metathesis

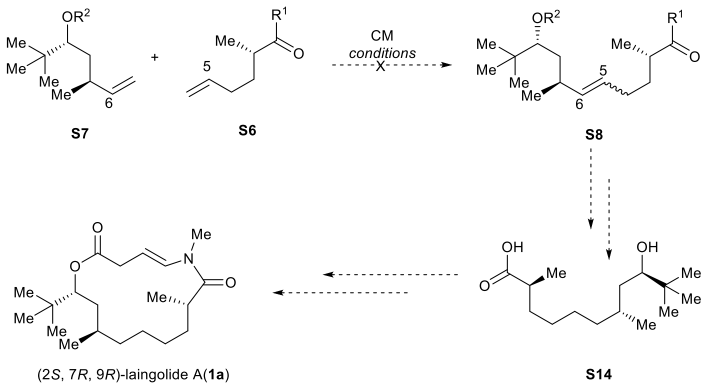

Scheme 1. Initial route towards laingolide A

Experimental Procedure:

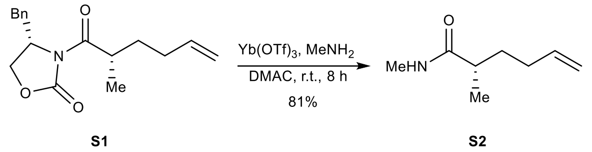

To a solution of $MeNH_2$ (2 M in THF, 2.6 mL, 5.2 mmol, 3.0 eq.) in dry DMAC (5 mL) was added $Yb(OTf)_3$ (108 mg, 0.17 mmol, .0.1 eq.) and **S1** [1] (500 mg, 1.7 mmol, 1.0 eq.) at room temperature under argon atmosphere. The reaction mixture was allowed to stir at room temperature for 8 h and then quenched with water (20 mL). The aqueous layer was extracted with EtOAc (3 × 30 mL). The combined organic layers were washed with brine, dried over anhydrous sodium sulfate, filtered, and concentrated under reduced pressure. Purification of the crude product was performed by flash column chromatography on silica gel (Hexanes/EtOAc = 5:1) to afford amide **S2** (194 mg, 81%) as a colorless oil.

TLC: R_f = 0.3 (Hexanes/EtOAc = 3:1), iodine & PMA stain.

$[\alpha]_D^{31}$ = +12.1 (*c* 1.4, $CHCl_3$).

^{1}H NMR (300 MHz, $CDCl_3$) δ 6.25 (s, 1H), 5.70 (ddt, *J* = 16.9, 10.1, 6.6 Hz, 1H), 5.04 – 4.78 (m, 2H), 2.72 (d, *J* = 4.7 Hz, 3H), 2.42 – 2.09 (m, 1H), 2.04 – 1.89 (m, 2H), 1.84 – 1.57 (m, 1H), 1.49 – 1.28 (m, 1H), 1.06 (d, *J* = 6.8 Hz, 3H).

^{13}C NMR (75 MHz, $CDCl_3$) δ 177.2, 138.2, 114.8, 40.5, 33.3, 31.6, 26.1, 17.9.

HRMS (ESI) calculated for $C_8H_{15}NONa^+$ $[M+Na]^+$ 164.1046, found 164.1049.

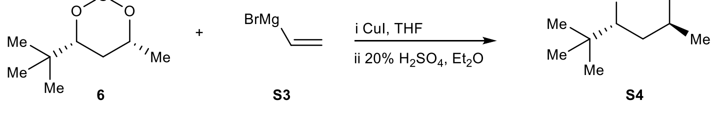

Table S1. Optimization of Cu-mediated vinylation

Entry	**S3**	CuI	Temperature	yield
1	5 equiv.	1.2 equiv.	-20 °C	trace

2	5 equiv.	1.2 equiv.	-20 °C~r.t.	trace
3	5 equiv.	1.2 equiv.	0 °C~r.t.	trace
4	3 equiv.	1.2 equiv.	0 °C~r.t.	trace
5	5 equiv.	0.6 equiv.	0 °C~r.t.	10%
6	5 equiv.	0.05 equiv.	0 °C~r.t.	20%
7	5 equiv.	0.1 equiv.	0 °C~r.t.	40%
8	5 equiv.	0.2 equiv.	0 °C~r.t.	35%

To a solution of cyclic sulfate **6** [2] (100 mg, 0.48 mmol, 1.0 eq.) and CuI in dry THF (1 mL) was add vinylmagnesium bromide (1.0 M in THF) under argon atmosphere. The purple-colored reaction mixture was allowed to stir until the cyclic sulfate **6** was consumed completely and then concentrated in *vacuo*. The solid residue was redissolved in Et_2O (5 mL) and treated with 20% aqueous H_2SO_4 (1.5 mL) solution. The contents of the flask were then stirred vigorously for another 12 h before the phases were separated. The aqueous layer was extracted with Et_2O (3 × 10 mL). The combined organic layers were dried over anhydrous sodium sulfate, filtered, and concentrated under reduced pressure. Purification of the crude product was performed by flash column chromatography on silica gel (Hexanes/EtOAc = 20:1) to afford alcohol **S4** as a colorless oil.

TLC: R_f = 0.6 (Hexanes/EtOAc = 10:1), iodine & PMA stain.

$[\alpha]_D^{31}$ = +13.7 (*c* 1.0, $CHCl_3$).

^{1}H NMR (400 MHz, $CDCl_3$) δ 5.70 – 5.53 (m, 1H), 5.02 (dd, *J* = 17.3, 1.8 Hz, 1H), 5.00 – 4.91 (m, 1H), 3.22 (dd, *J* = 10.3, 2.3 Hz, 1H), 2.79 – 2.28 (m, 1H), 1.51 (s, 1H), 1.40 – 1.27 (m, 2H), 1.03 (d, *J* = 6.8 Hz, 3H), 0.87 (s, 9H).

^{13}C NMR (100 MHz, $CDCl_3$) δ 144.2, 113.8, 77.3, 38.6, 35.3, 34.8, 25.8, 21.8.

HRMS (ESI) calculated for $C_{10}H_{20}ONa^+$ $[M+Na]^+$ 179.1406, found 179.1410

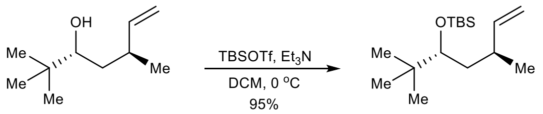

S4 **S5**

To a solution of alcohol **S4** (180 mg, 1.15 mmol, 1.0 eq.) in dry DCM (5 mL, 0.23 M) was added Et_3N (2.3 mmol, 0.32 mL, 2.0 eq.) and TBSOTf (1.38 mmol, 0.32 mL, 1.2 eq.) at 0 °C. The reaction mixture was allowed to stir at 0 °C for 2 h before it was diluted with DCM (5 mL) and quenched with saturated aqueous solution of NH_4Cl (5 mL). The aqueous layer was extracted with DCM (3 × 10 mL). The combined organic layers were washed with brine (10 mL), dried over anhydrous sodium sulfate, filtered, and concentrated under reduced pressure. Purification of the crude product was performed by flash chromatography on silica gel (Hexanes) to afford silyl ether **S5** (295 mg, 95%) as a colorless oil.

TLC: R_f = 0.95 (Hexanes), iodine & PMA stain.

$[\alpha]_D^{31}$ = +8.6 (*c* 1.0, $CHCl_3$).

^{1}H NMR (400 MHz, $CDCl_3$) δ 5.60 (ddd, *J* = 17.3, 10.3, 8.0 Hz, 1H), 5.02 – 4.97 (m, 1H), 4.95 (ddd, *J* = 10.4, 1.9, 0.8 Hz, 1H), 3.26 (dd, *J* = 7.5, 1.9 Hz, 1H), 2.38 – 2.27 (m, 1H), 1.50 (ddd, *J* = 14.3, 10.6,

1.9 Hz, 1H), 1.28 (ddd, *J* = 14.3, 7.5, 3.5 Hz, 1H), 1.00 (d, *J* = 6.7 Hz, 3H), 0.90 (s, 9H), 0.84 (s, 9H), 0.08 (s, 3H), 0.05 (s, 3H).
^{13}C NMR (100 MHz, $CDCl_3$) δ 144.8, 113.3, 78.9, 41.0, 35.8, 35.3, 26.4, 26.4, 22.2, 18.7, -3.0, -3.6.
HRMS (ESI) calculated for $C_{16}H_{34}OSiNa^+$ $[M+Na]^+$ 293.2271, found 293.2275.

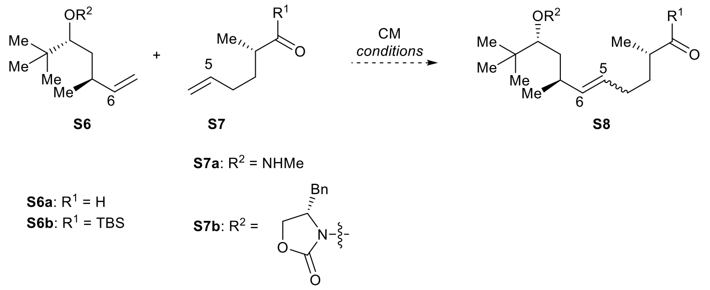

Table S2. Optimization of the Cross metathesis

Entry	S7	S6	S7/S6	Condition	Result
1	**S7a**	**S6a**	5:1	G-I, benzoquinone, DCM, 40 °C	trace
2	**S7a**	**S6a**	5:1	G-II, benzoquinone, DCM, 40 °C	trace
3	**S7a**	**S6a**	5:1	HG-II, benzoquinone, DCM, 40 °C	trace
4	**S7a**	**S6a**	10:1	G-I, benzoquinone, DCM, 40 °C	trace
5	**S7a**	**S6a**	10:1	G-II, benzoquinone, DCM, 40 °C	trace
6	**S7a**	**S6a**	10:1	HG-II, benzoquinone, DCM, 40 °C	trace
7	**S7a**	**S6a**	10:1	G-I, benzoquinone, toluene, 40 °C	trace
8	**S7a**	**S6a**	10:1	G-II, benzoquinone, toluene, 40 °C	trace
9	**S7a**	**S6a**	10:1	HG-II, benzoquinone, toluene, 40 °C	trace
10	**S7a**	**S6b**	10:1	G-II, benzoquinone, DCM, 40 °C	trace
11	**S7a**	**S6b**	10:1	G-I, benzoquinone, DCM, 40 °C	trace
12	**S7a**	**S6b**	10:1	HG-II, benzoquinone, DCM, 40 °C	trace
13	**S7b**	**S6b**	10:1	G-II, benzoquinone, DCM, 40 °C	trace
14	**S7b**	**S6b**	10:1	G-I, benzoquinone, DCM, 40 °C	trace
15	**S7b**	**S6b**	10:1	HG-II, benzoquinone, DCM, 40 °C	trace

2. synthesis of *ent*-8

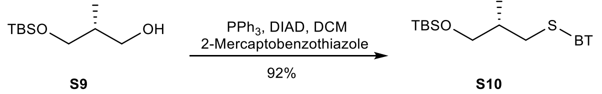

To a solution of alcohol **S9** [3] (3.3 g, 16.2 mmol, 1.0 eq.), Ph_3P (5.1 g, 19.4 mmol, 1.2 eq.) and 2-mercaptobenzthiazol (3.25 g, 19.4 mmol, 1.2 eq.) in dry DCM (80 mL, 0.2 M) under argon

atmosphere, was added DIAD (3.83 mL, 19.4 mmol, 1.2 eq.) at 0 °C. The mixture was stirred for 2 h at the room temperature before it was diluted with DCM (20 mL) and then quenched with saturated aqueous solution of $NaHCO_3$ (100 mL). The aqueous layer was separated and extracted with DCM (3 × 100 mL). The combined organic layers were washed with brine, dried over anhydrous sodium sulfate, filtered, and concentrated under reduced pressure. Purification of the crude product was performed by flash column chromatography on silica gel (Hexanes/ EtOAc = 10:1-5:1) to afford **S10** (5.26 g, 92%) as a colorless oil.

TLC: R_f = 0.6 (Hexanes/EtOAc = 4:1), UV & PMA stain.

$[\alpha]_D^{25}$ = +2.8 (*c* 0.005, $CHCl_3$).

^{1}H NMR (400 MHz, $CDCl_3$) δ 7.85 (ddd, *J* = 8.2, 1.2, 0.6 Hz, 1H), 7.75 (ddd, *J* = 8.0, 1.3, 0.6 Hz, 1H), 7.40 (ddd, *J* = 8.3, 7.3, 1.3 Hz, 1H), 7.31 – 7.26 (m, 1H), 3.65 (dd, *J* = 10.0, 5.1 Hz, 1H), 3.56 (dd, *J* = 10.0, 5.9 Hz, 1H), 3.51 (dd, *J* = 12.9, 6.2 Hz, 1H), 3.23 (dd, *J* = 12.9, 7.1 Hz, 1H), 2.24 – 2.06 (m, 1H), 1.07 (d, *J* = 6.8 Hz, 3H), 0.91 (s, 9H), 0.06 (s, 3H), 0.06 (s, 3H).

^{13}C NMR (100 MHz, $CDCl_3$) δ 167.9, 153.5, 135.3, 126.1, 124.2, 121.6, 121.0, 66.6, 37.2, 36.1, 26.1, 18.5, 16.4, -5.2, -5.3.

HRMS (ESI) calculated for $C_{17}H_{28}NOS_2Si^+$ $[M+H]^+$ 354.1376, found 354.1376.

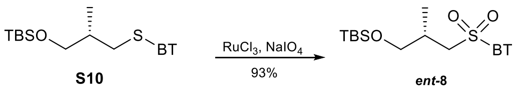

To a solution of **S10** (2.0 g, 5.66 mmol, 1.0 eq.) and $NaIO_4$ (3.6 g, 17.0 mmol, 3.0 eq.) in CCl_4 (11.0 mL), CH_3CN (22.0 mL), and H_2O (22.0 mL) was added $RuCl_3{\cdot}nH_2O$ (0.6 mg, 2.8 μmol, 0.05 eq.) at room temperature. The reaction mixture was stirred for 1 h and diluted with Et_2O (50 mL) and quenched with saturated aqueous solution of $NaHCO_3$ (30 mL). The aqueous layer was extracted with Et_2O (3 × 50 mL). The combined organic layers were washed with brine, dried over anhydrous sodium sulfate, filtered, and concentrated under reduced pressure. Purification of the crude product was performed by flash column chromatography on silica gel (Hexanes/EtOAc = 8:1-4:1) to afford sulfone ***ent*-8** (2.03 g, 93%) as a colorless oil.

TLC: R_f = 0.5 (Hexanes/EtOAc = 4:1), UV & PMA stain.

$[\alpha]_D^{27}$ = +3.9 (*c* 0.01, $CHCl_3$).

^{1}H NMR (400 MHz, $CDCl_3$) δ 8.21 (dd, *J* = 8.2, 1.1 Hz, 1H), 8.10 – 7.91 (m, 1H), 7.63 (td, *J* = 7.7, 1.5 Hz, 1H), 7.58 (td, *J* = 7.6, 1.4 Hz, 1H), 3.83 (dd, *J* = 14.5, 4.5 Hz, 1H), 3.64 (dd, *J* = 10.0, 4.8 Hz, 1H), 3.43 (dd, *J* = 10.0, 6.2 Hz, 1H), 3.29 (dd, *J* = 14.5, 8.0 Hz, 1H), 2.53 – 2.32 (m, 1H), 1.12 (d, *J* = 6.8 Hz, 3H), 0.82 (s, 9H), -0.01 (s, 3H), -0.03 (s, 3H).

^{13}C NMR (100 MHz, $CDCl_3$) δ 166.5, 152.8, 136.9, 128.1, 127.7, 125.6, 122.4, 66.4, 57.7, 31.7, 25.9, 18.3, 16.9, -5.4, -5.4.

HRMS (ESI) calculated for $C_{17}H_{27}NO_3S_2SiNa^+$ $[M+Na]^+$ 408.1094, found 408.1095.

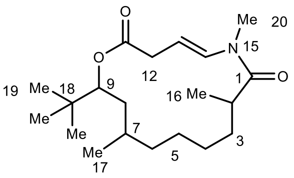

Reported Structure of Isolated laingolide A

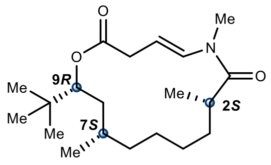

Assigned Structure of (+)-laingolide A(**1c**)

3. Comparison of ^{1}H NMR and ^{13}C NMR data of laingolide A

Table 1 Comparison of ^{1}H NMR data of laingolide A (Natural Product and Synthetic Sample)

NO.	laingolide A				
	Natural (δ_1)	Sample **1a** (δ_2)	Sample **1b** (δ_3)	Sample **1c** (δ_4)	Sample **1d** (δ_5)
1	-	-	-	-	-
2	2.99, m	2.86–2.72, m	2.91, dt (13.4, 6.5)	3.03–2.95, m	2.68, dqd (12.9, 6.6, 4.4)
3a	1.56, m	1.80–1.63, m	1.58–1.51, m	1.60–1.52, m	1.49–1.33, m
3b	1.40, m	1.59–1.52, m	1.58–1.51, m	1.47–1.34, m	1.49–1.33, m
4a	1.24, m	1.40–1.33, m	1.33–1.25, m	1.30–1.17, m	1.25–1.23, m
4b	1.04, m	1.25–1.17, m	1.10–0.99, m	1.17–1.12, m	1.14, d (6.4)
5a	1.26, m	1.40–1.33, m	1.33–1.25, m	1.30–1.17, m	1.29–1.25, m
5b	1.12, m	1.25–1.17, m	1.25–1.18, m	1.17–1.12, m	1.22–1.16, m
6a	1.32, m	1.46–1.40, m	1.46–1.36, m	1.35–1.27, m	1.49–1.33, m
6b	1.23, m	1.32–1.27, m	1.33–1.25, m	1.30–1.17, m	1.25–1.23, m
7	1.17, m	1.32–1.27, m	1.25–1.18, m	1.30–1.17, m	1.22–1.16, m
8a	1.58, m	1.80–1.63, m	1.65–1.61, m	1.64–1.57, m	1.63–1.50, m
8b	0.99, m	0.96–0.91, m	0.85–0.79, m	1.03–0.95, m	1.14–1.05, m
9	4.81, dd (11.0, <1.0)	4.94, dd (11.1, 2.5)	4.92, dd (11.9, 1.8)	4.81, dd (11.3, 1.3)	4.86, dd (10.9, 1.3)
10(O)	-	-	-	-	-
11	-	-	-	-	-
12a	3.06, ddd (12.0, 6.0, 1.5)	3.17–3.08, m	3.05, ddd (13.3, 7.4, 0.9)	3.07, ddd (12.3, 5.6, 1.5)	3.2, ddd (16.5, 6.3, 1.4)
12b	2.94, dd (12.0, 10.8)	2.98, ddd (16.0, 9.5, 0.8)	2.99, ddd (13.3, 6.9, 1.4)	2.94, dd (12.3, 10.4)	3.07, ddd (16.5, 8.7, 1.0)
13	5.18, ddd (14.0, 10.8, 6.0)	5.21, ddd (13.8, 9.4, 6.0)	5.16, dt (14.1, 7.2)	5.18, ddd (13.6, 10.3, 5.7)	5.21, ddd (13.8, 8.6, 6.3)
14	7.01, dd (14.0, 1.5)	6.76, d (13.8)	7.09, d (13.9)	7.02, dd (13.7, 1.4)	6.74, d (13.9)
15(N)	-	-	-	-	-

16	1.16, 3H, d (6.6)	1.15, 3H, d (6.5)	1.15, 3H, d (6.6)	1.15, 3H, d (6.5)	1.14, 3H, d (6.4)
17	0.83, 3H, d (7.0)	0.89, 3H, s	0.78, 3H, d (6.4)	0.84, 3H, d (5.7)	0.85, 3H, d (5.8)
18	-	-	-	-	-
19	0.89, 9H, s	0.88, 9H, s	0.88, 9H, s	0.89, 9H, s	0.88, 9H, s
20	3.10, 3H, s	3.11, 3H, s	3.09, 3H, s	3.10, 3H, s	3.11, 3H, s
21	-	-	-	-	-

Table 2 Comparison of ^{13}C NMR data of laingolide A
(Natural Product and Synthetic Sample)

No.	laingolide A								
	Natural (δ_1)	Sample **1a** (δ_2)	$\Delta_\delta=\delta_1-\delta_2$	Sample **1b** (δ_3)	$\Delta_\delta=\delta_1-\delta_3$	Sample **1c** (δ_4)	$\Delta_\delta=\delta_1-\delta_4$	Sample **1d** (δ_5)	$\Delta_\delta=\delta_1-\delta_5$
1	176.4	176.3	0.1	176.5	-0.1	176.4	0	176.6	-0.2
2	36.2	35.4	0.8	35.4	0.8	36.2	0	35.6	0.6
3	36.6	35.7	0.9	35.7	0.9	36.6	0	36.2	0.4
4	26.2	26.7	-0.5	26.1	0.1	26.2	0	24.9	1.3
5	26.8	27.6	-0.8	26.7	0.1	26.8	0	26.6	0.2
6	36.8	37.1	-0.3	37.3	-0.5	36.8	0	37.5	-0.7
7	27.7	30.5	-2.8	26.7	1	27.7	0	26.9	0.8
8	35.5	34.3	1.2	35.3	0.2	35.5	0	35.0	0.5
9	79.7	77.9	1.8	78.5	1.2	79.7	0	80.5	-0.8
10									
11	172.5	173.3	-0.8	172.1	0.4	172.5	0	171.9	0.6
12	37.6	37.7	-0.1	38.6	-1	37.6	0	38.7	-1.1
13	105.0	104.2	0.8	103.6	1.4	105.0	0	106.8	-1.8
14	133.6	133.4	0.2	133.8	-0.2	133.5	0.1	132.6	1
15									
16	18.5	17.2	1.3	18.0	0.5	18.5	0	16.6	1.9
17	21.2	20.1	1.1	20.9	0.3	21.2	0	18.9	2.3
18	35.1	34.2	0.9	34.1	1	35.1	0	33.7	1.4
19	26.7	27.4	-0.7	26.6	0.1	26.7	0	25.3	1.4
20	31.2	31.3	-0.1	31.4	-0.2	31.2	0	32.2	-1

4. References

[1] Ghosh, A.K.; and Gong, G. L. *J. Am. Chem. Soc.* **2004**, *126*, 3704.
[2] Tello-Aburto, R.; Newar, T. D.; Maio, W.A. *J. Org. Chem.* **2012**, *77*, 6271.
[3] Millán, A.; Martinez, P. D. G.; Aggarwal, V. K. *Chem. Eur. J.* **2018**, *24*, 730.

5. NMR spectra

^{1}H NMR Spectrum of **S2** (300 MHz, $CDCl_3$)

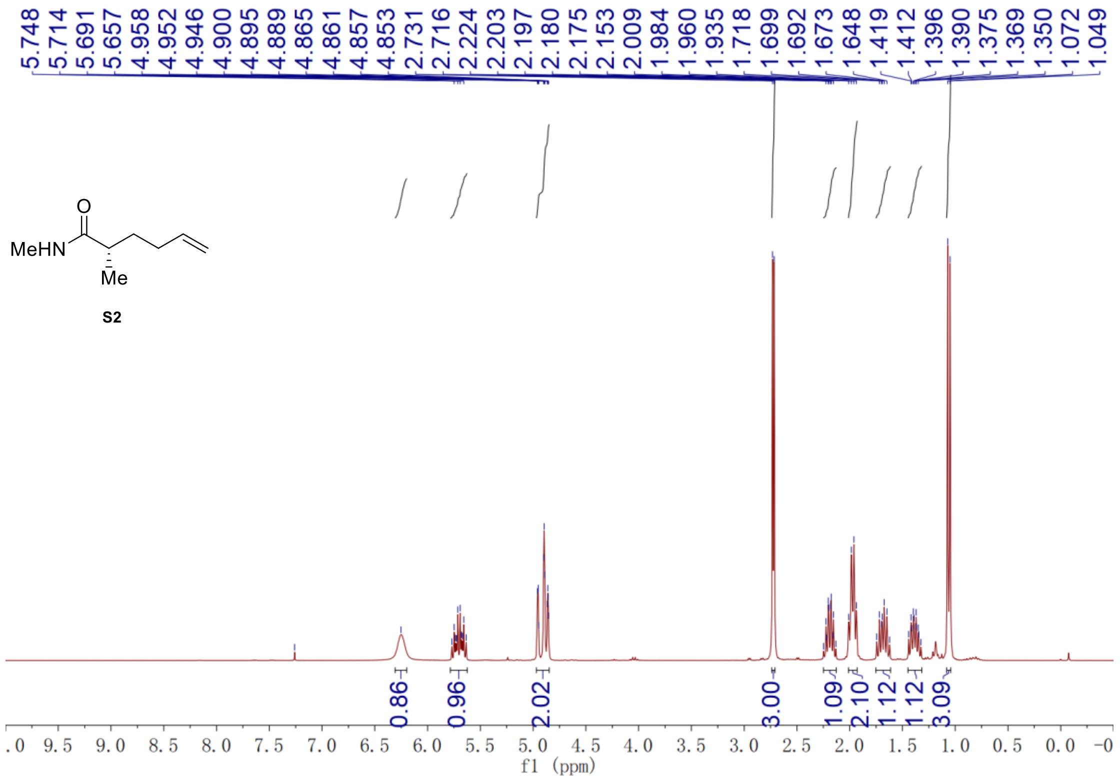

^{13}C NMR Spectrum of **S2** (75 MHz, $CDCl_3$)

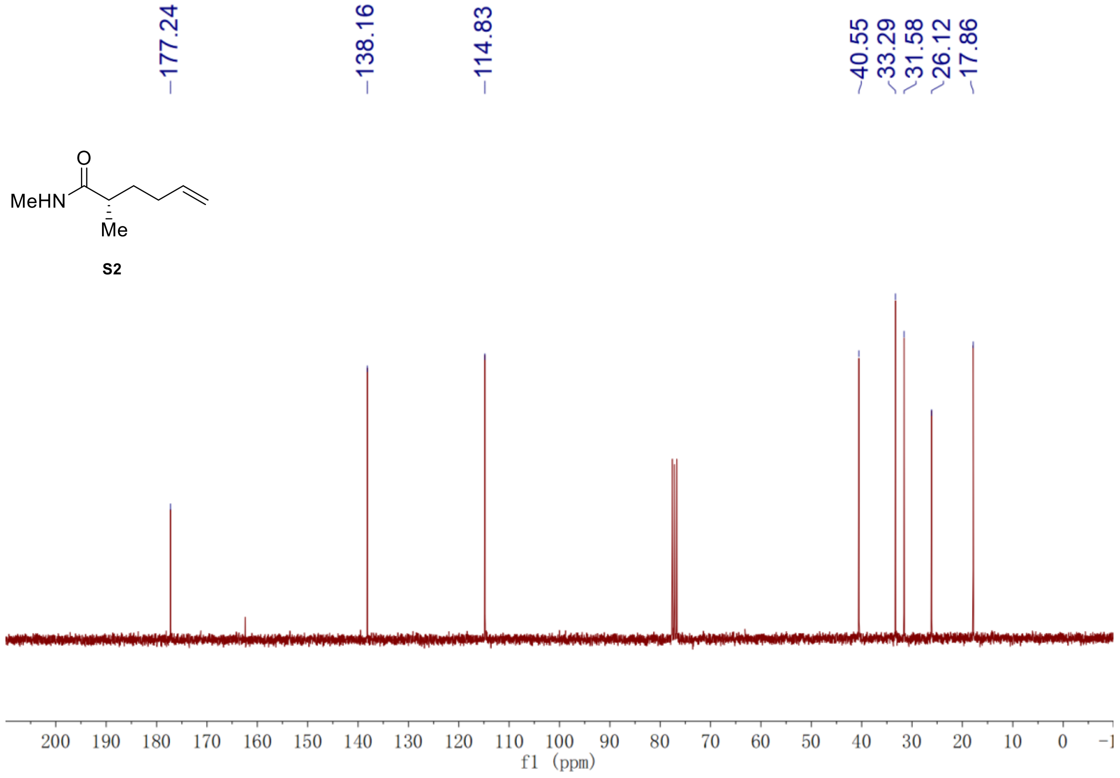

^{1}H NMR Spectrum of **S4** (400 MHz, $CDCl_3$)

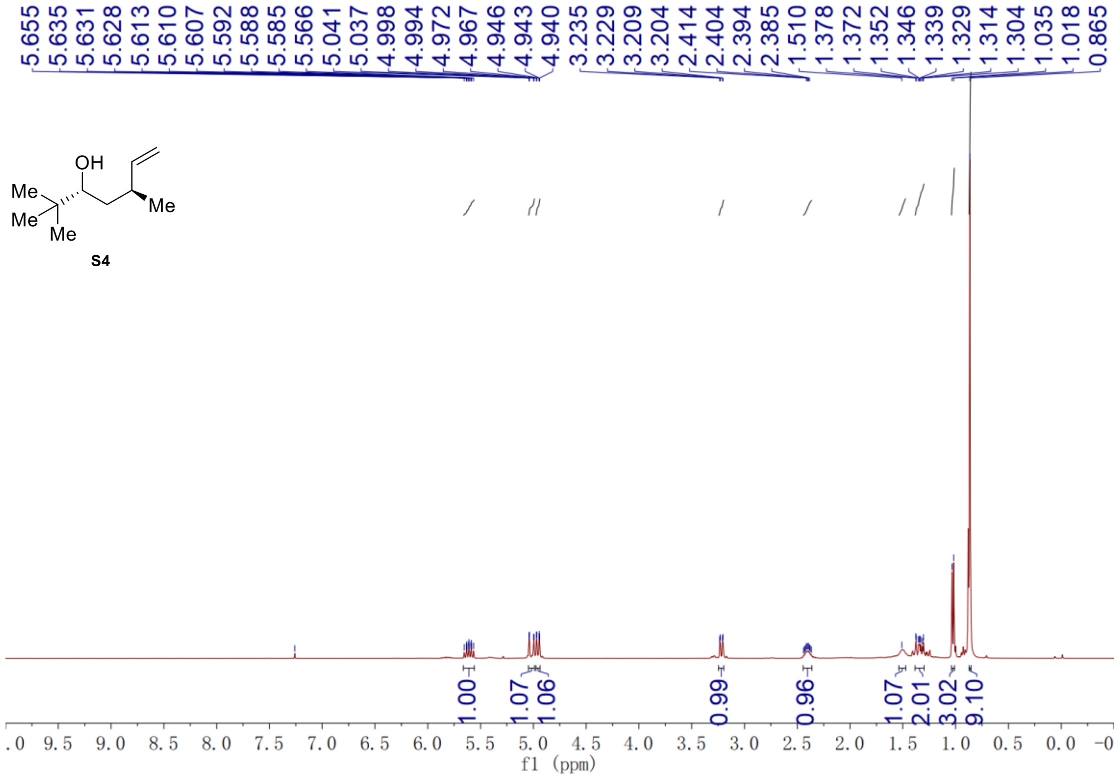

^{13}C NMR Spectrum of **S4** (100 MHz, $CDCl_3$)

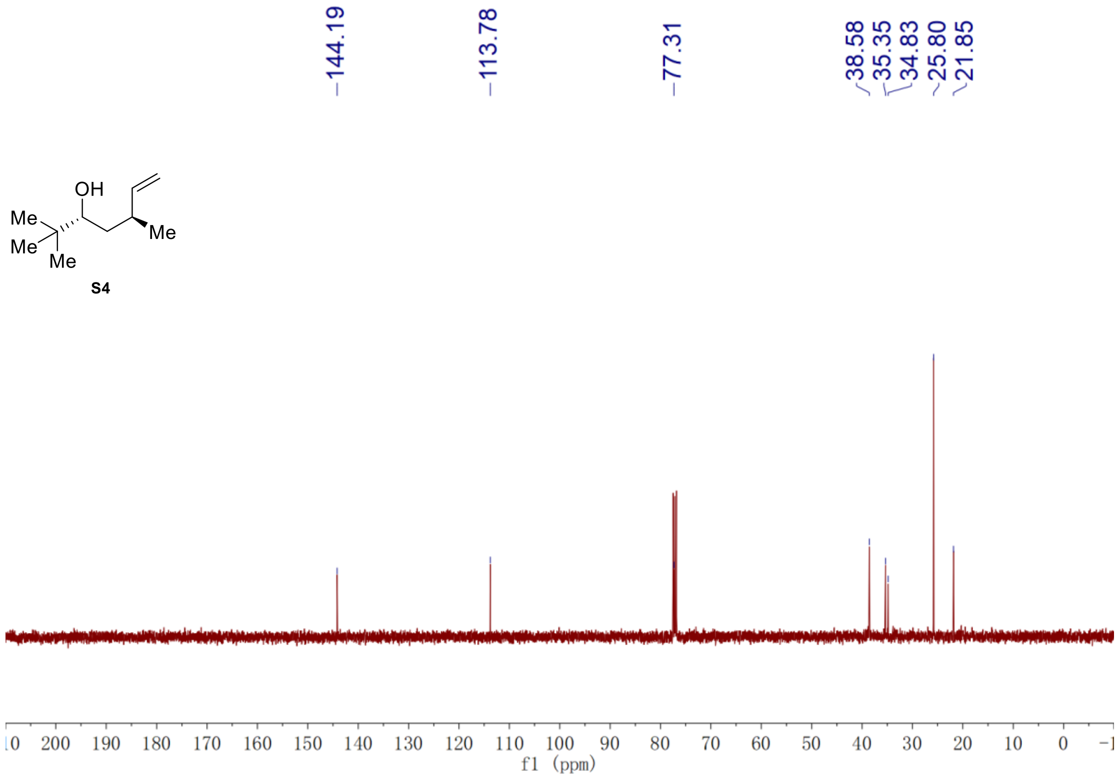

^{1}H NMR Spectrum of **S5** (400 MHz, $CDCl_3$)

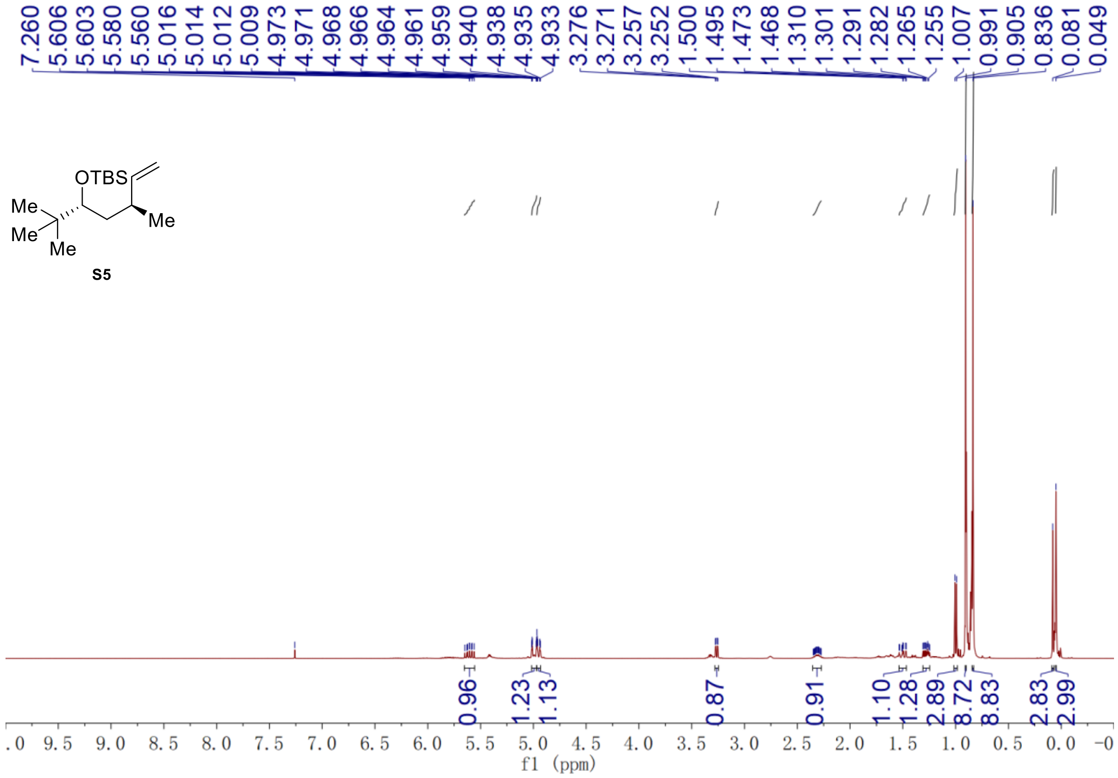

^{13}C NMR Spectrum of **S5** (100 MHz, $CDCl_3$)

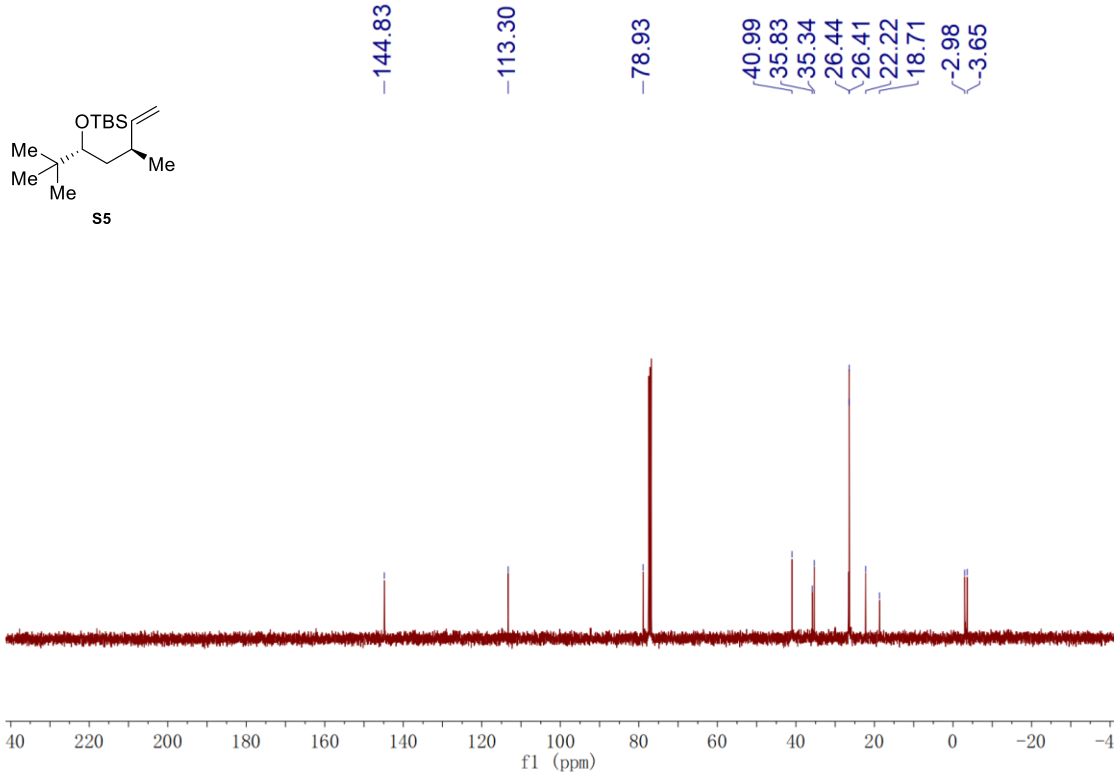

^{1}H NMR Spectrum of **S10** (400 MHz, $CDCl_3$)

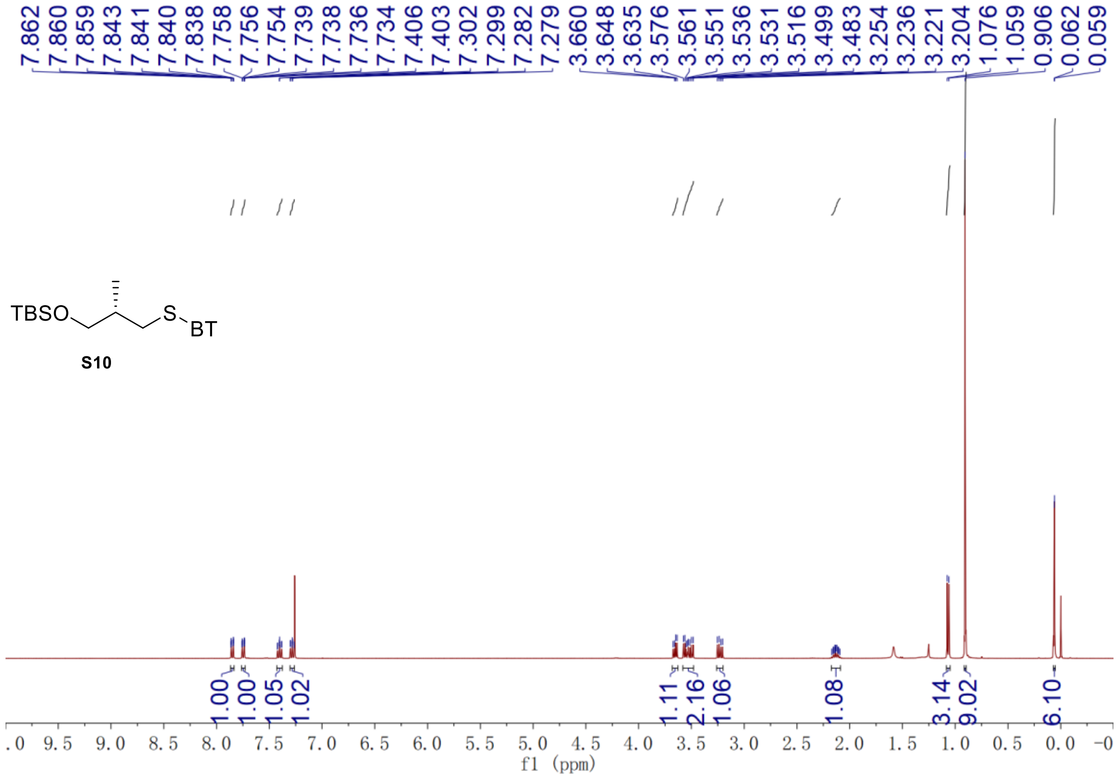

^{13}C NMR Spectrum of **S10** (100 MHz, $CDCl_3$)

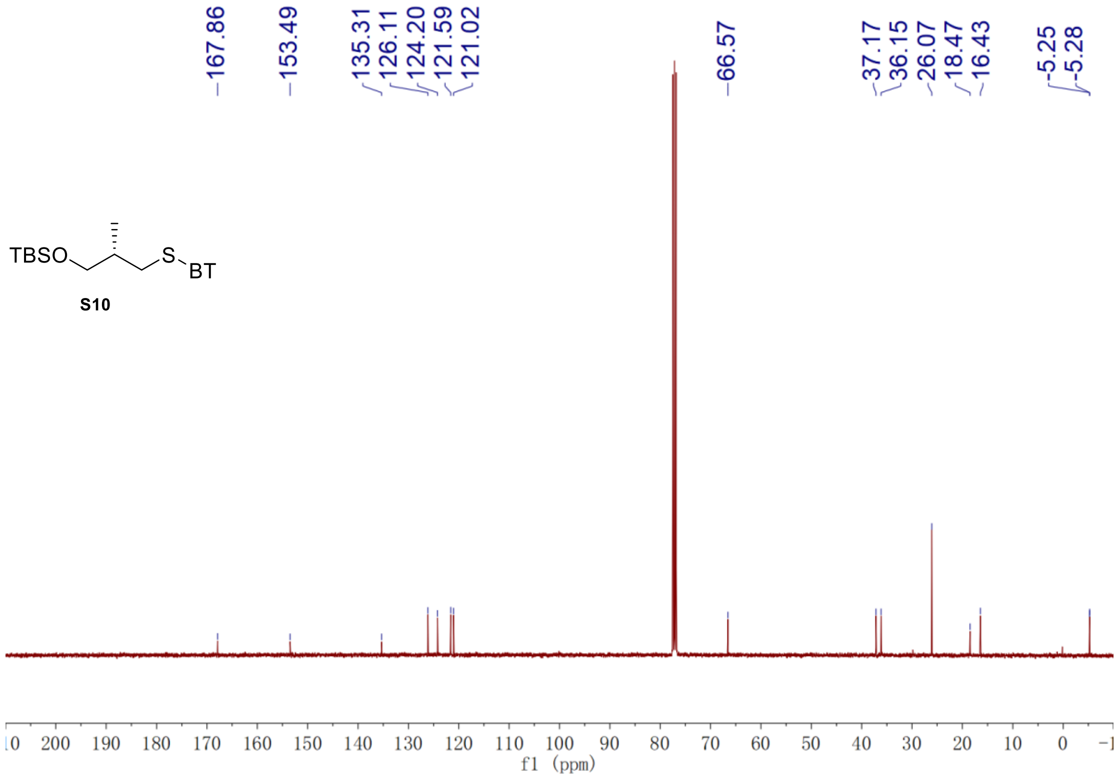

^{1}H NMR Spectrum of ***ent*-8** (400 MHz, $CDCl_3$)

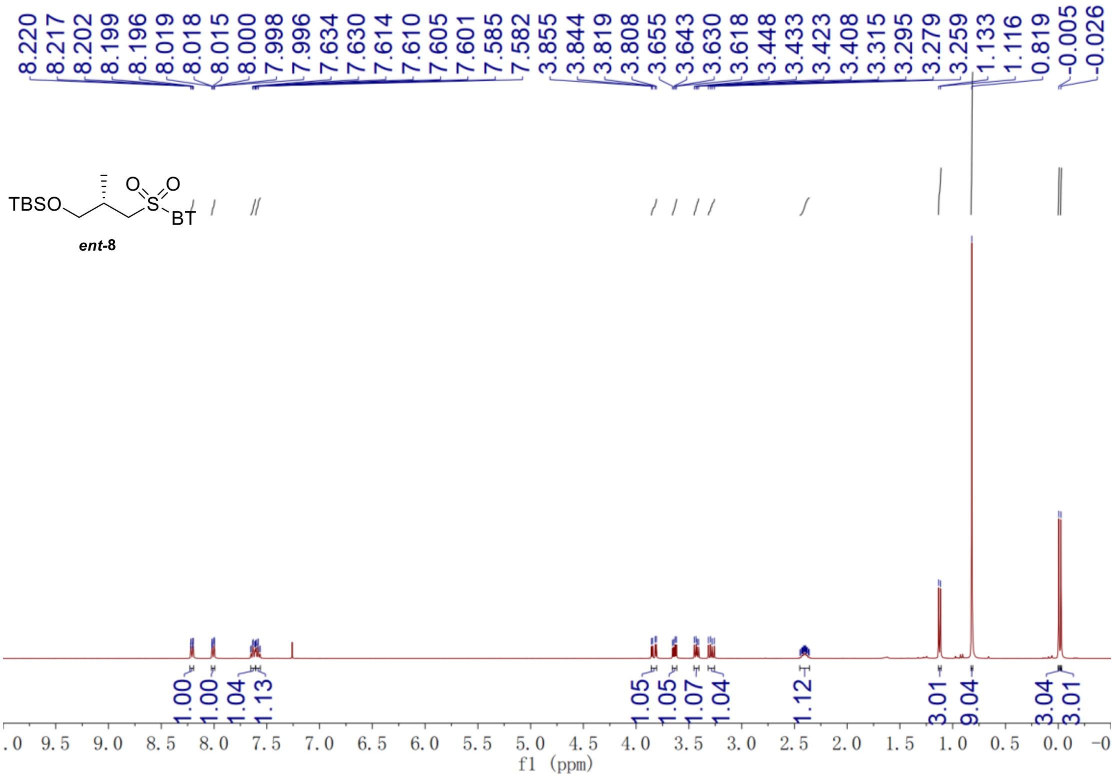

^{13}C NMR Spectrum of ***ent*-8** (100 MHz, $CDCl_3$)

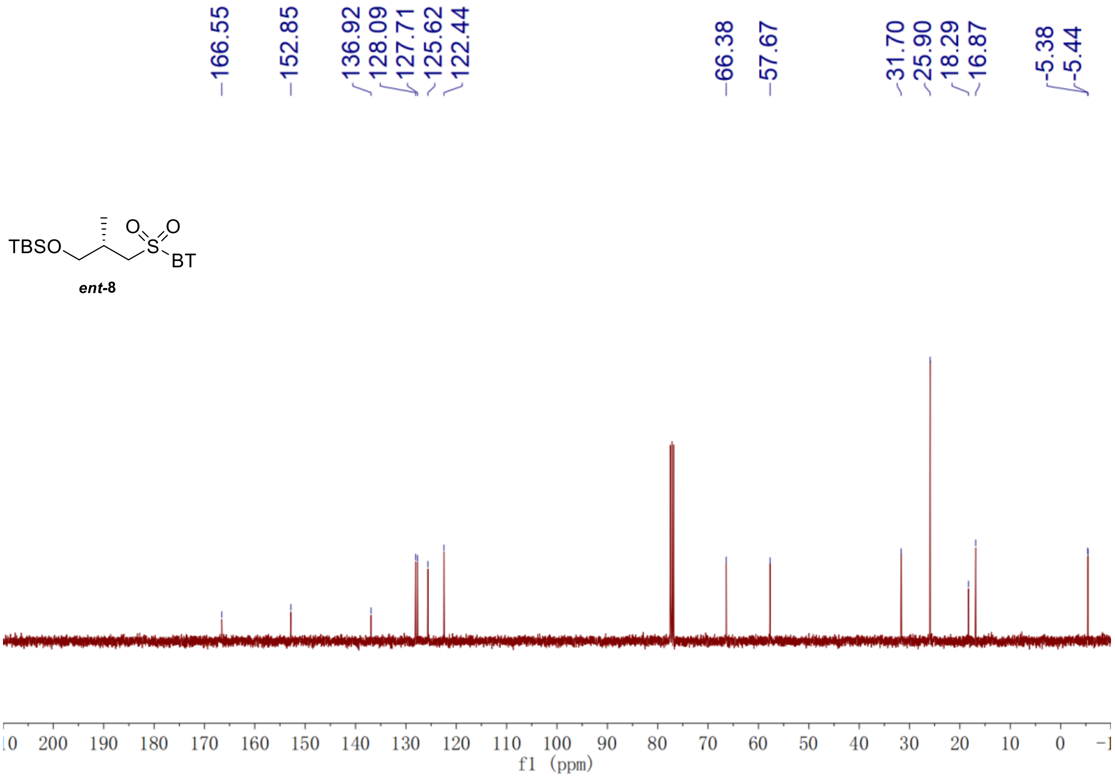

^{1}H NMR Spectrum of **S11** (500 MHz, $CDCl_3$)

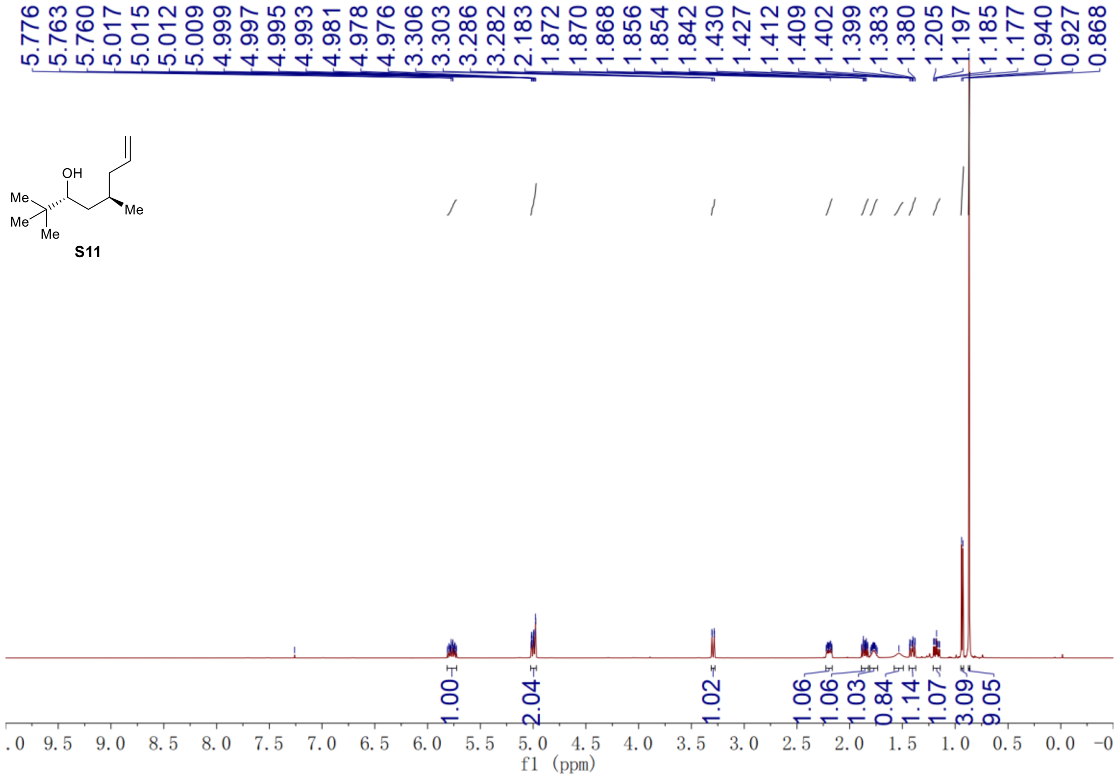

^{13}C NMR Spectrum of **S11** (125 MHz, $CDCl_3$)

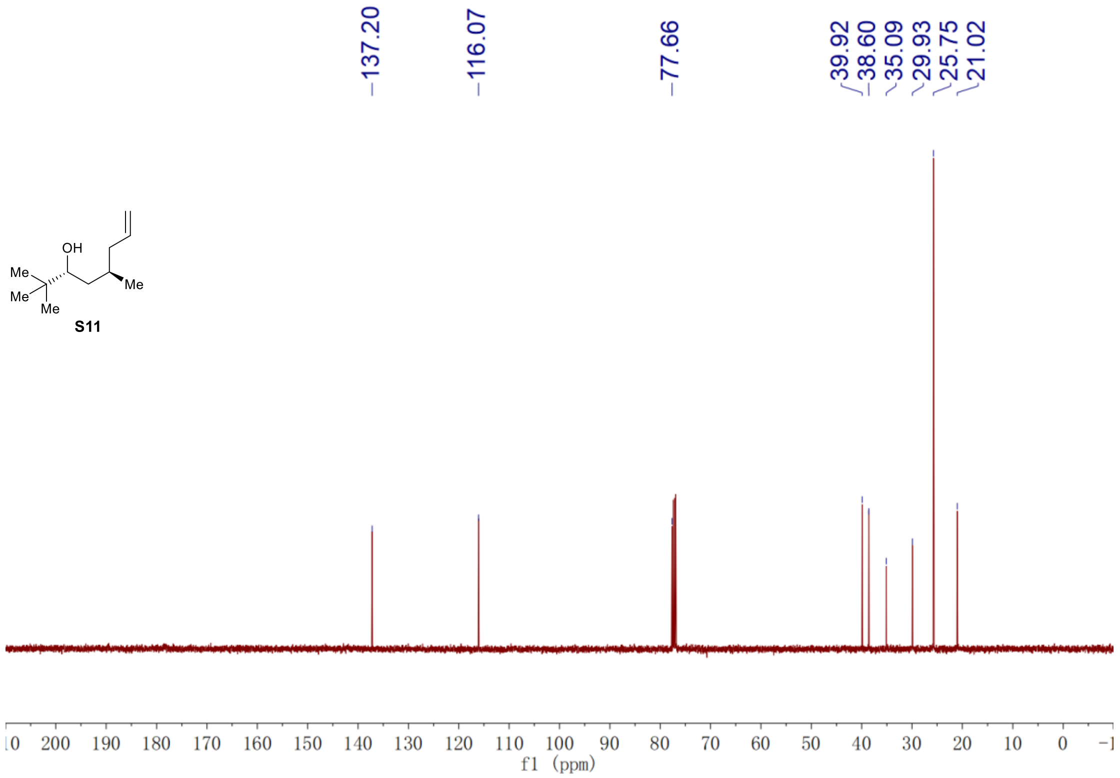

^{1}H NMR Spectrum of **10** (500 MHz, $CDCl_3$)

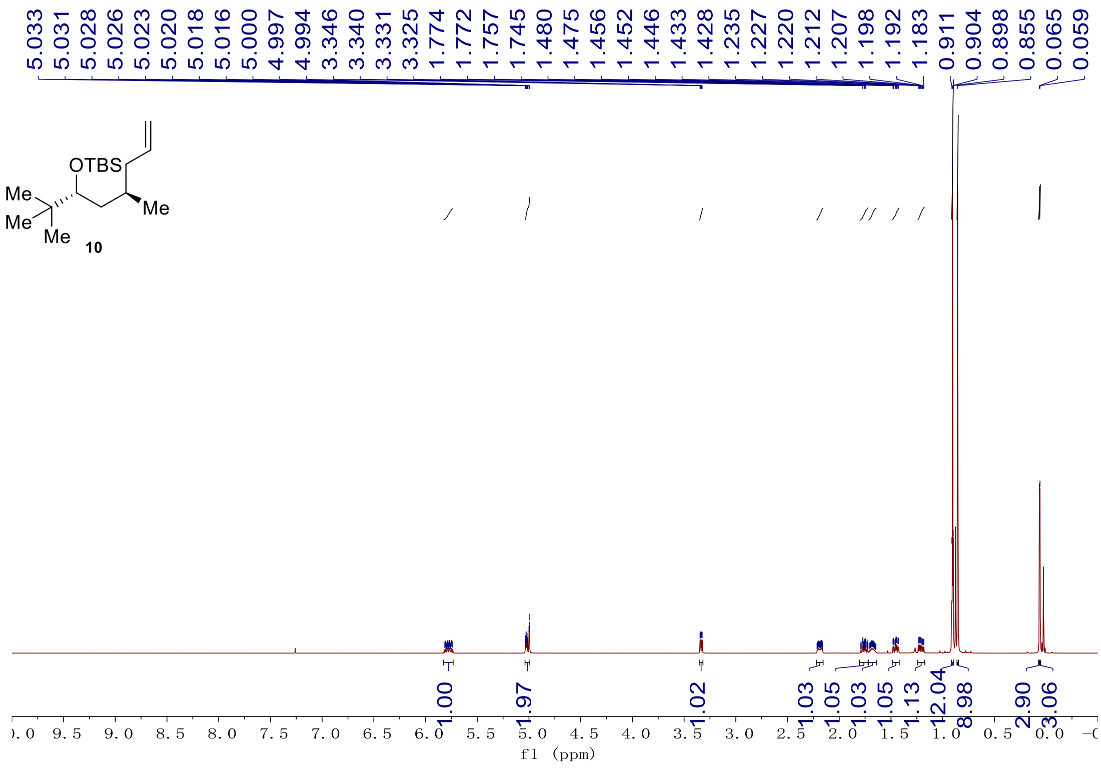

^{13}C NMR Spectrum of **10** (125 MHz, $CDCl_3$)

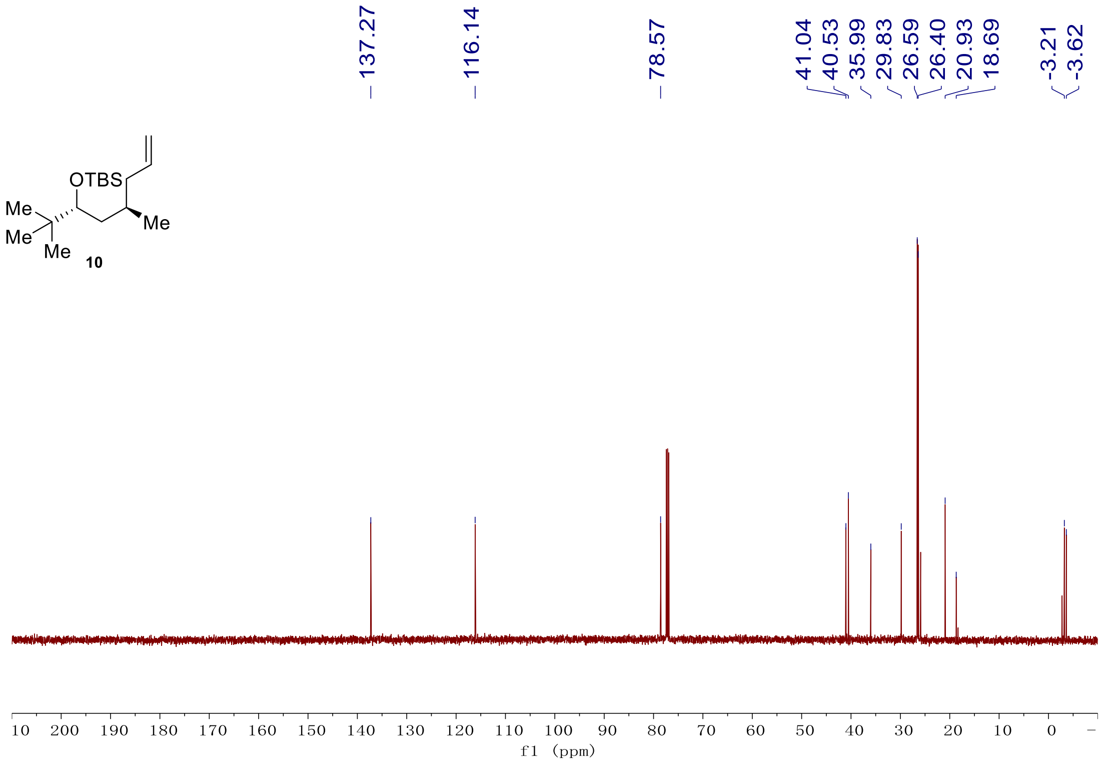

^{1}H NMR Spectrum of **11** (400 MHz, $CDCl_3$)

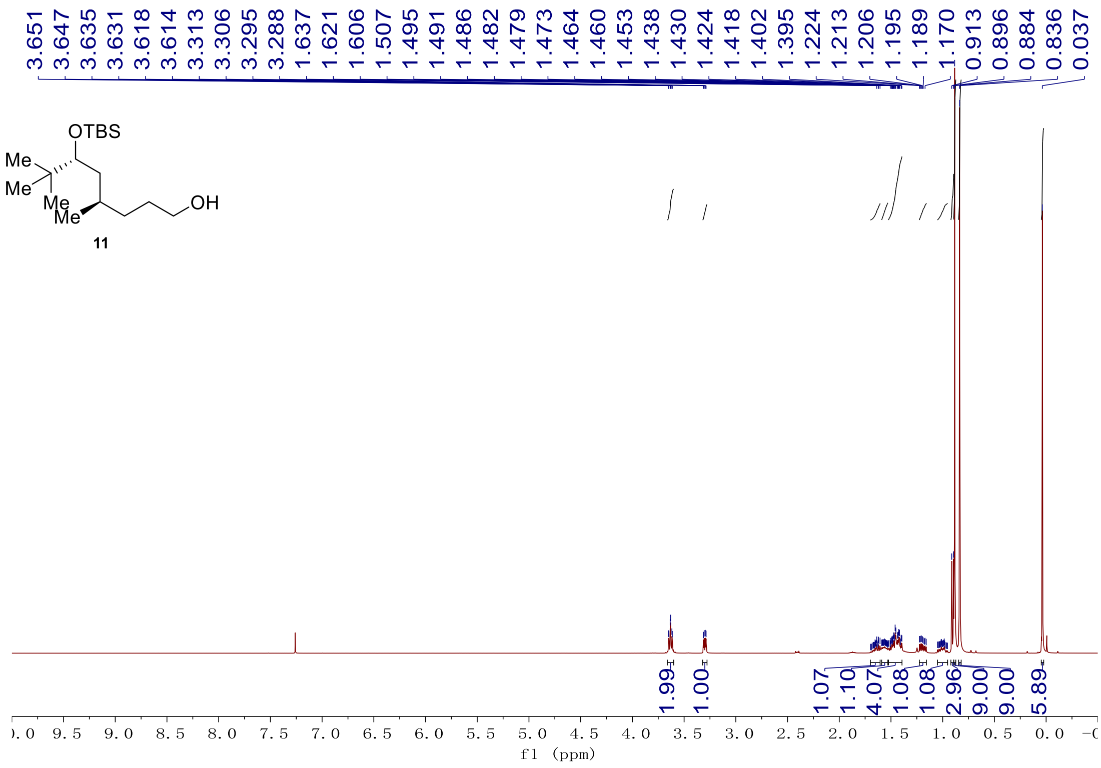

^{13}C NMR Spectrum of **11** (100 MHz, $CDCl_3$)

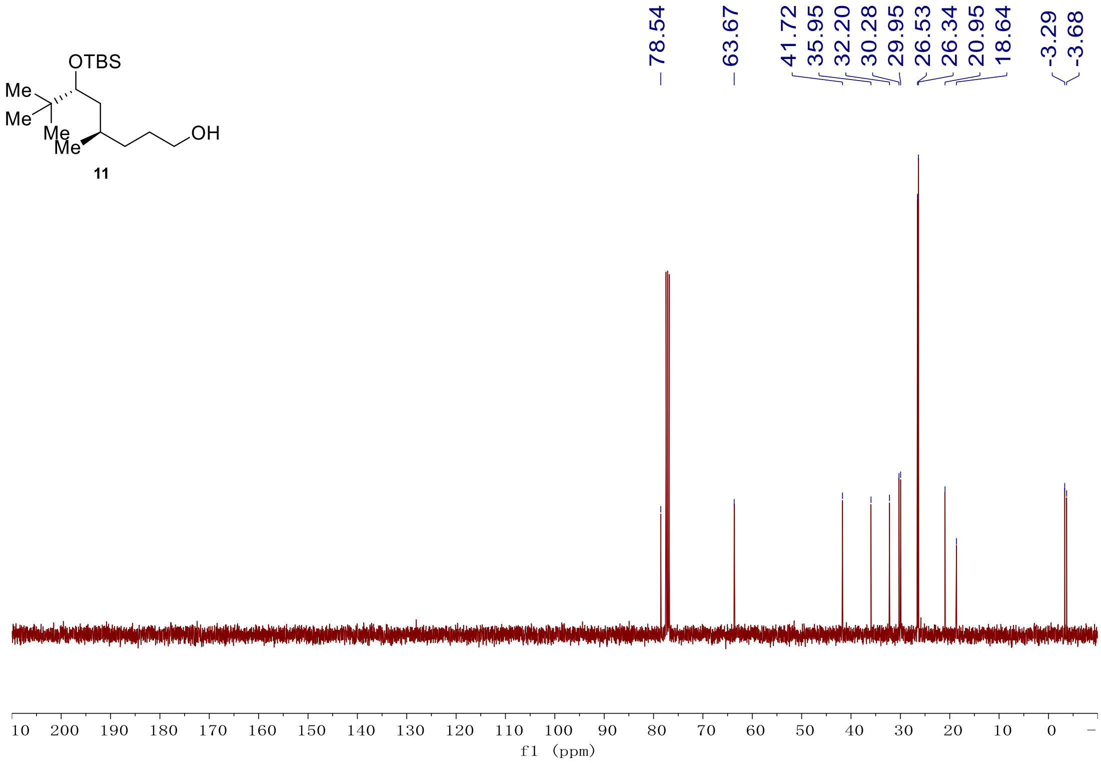

^{1}H NMR Spectrum of **12** (500 MHz, $CDCl_3$)

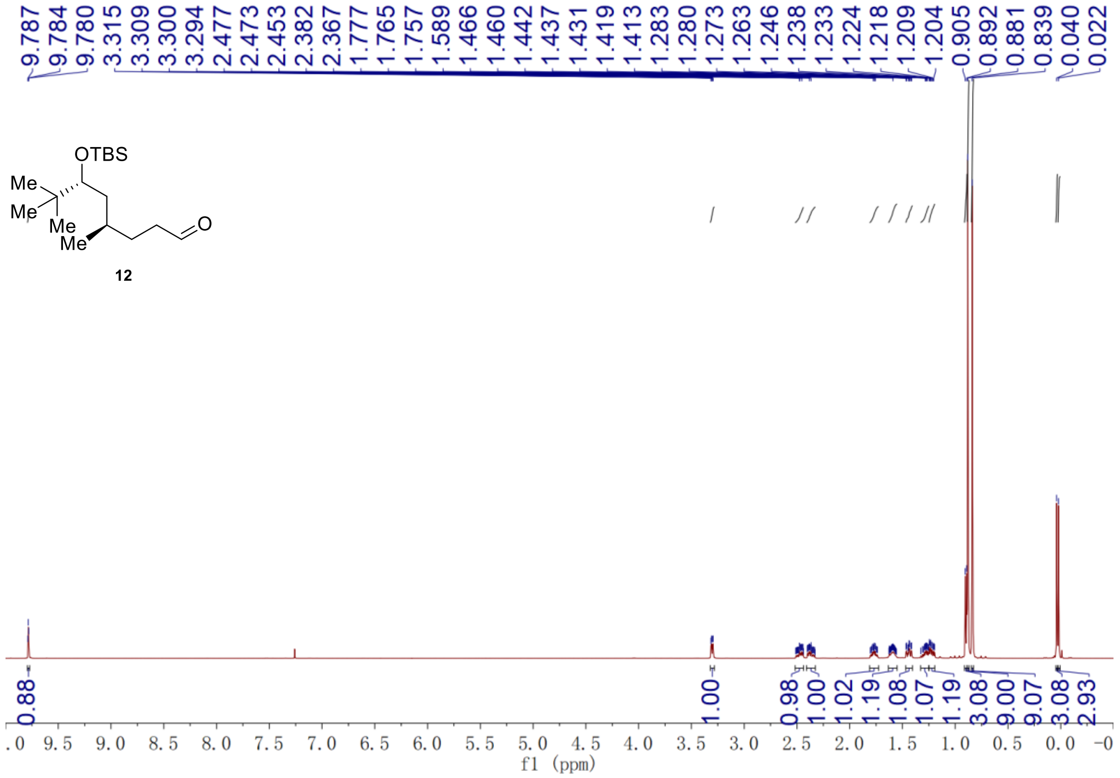

^{13}C NMR Spectrum of **12** (125 MHz, $CDCl_3$)

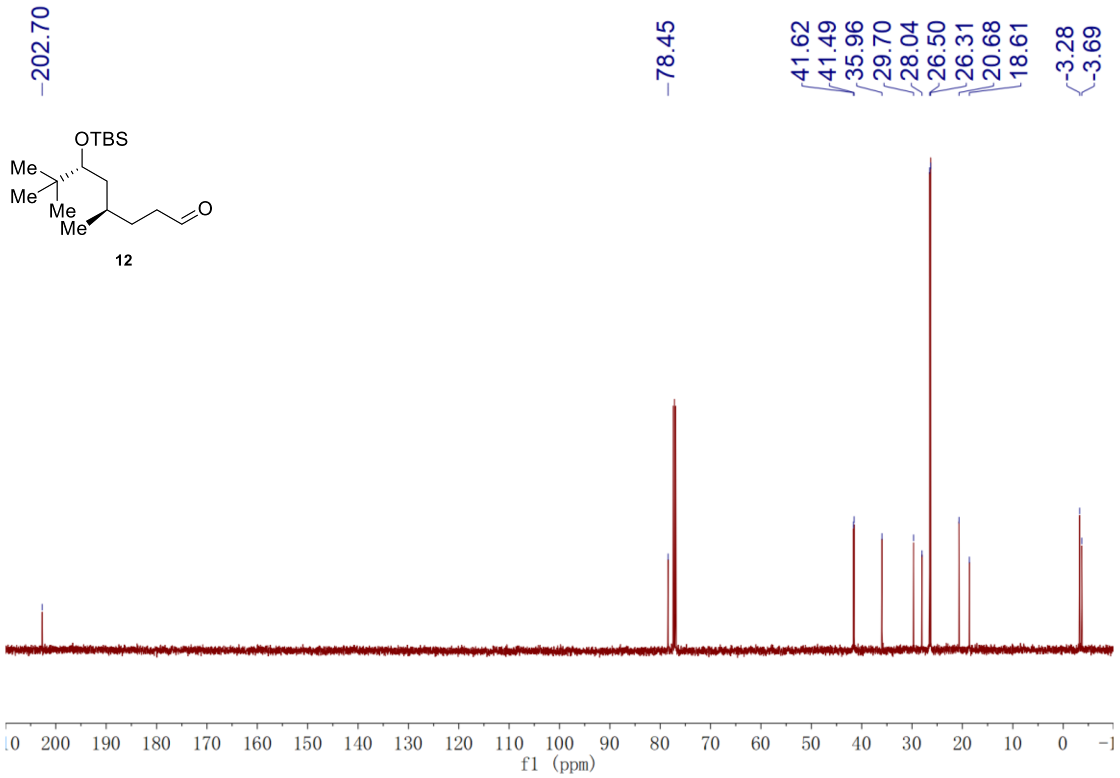

^{1}H NMR Spectrum of **7** (400 MHz, $CDCl_3$)

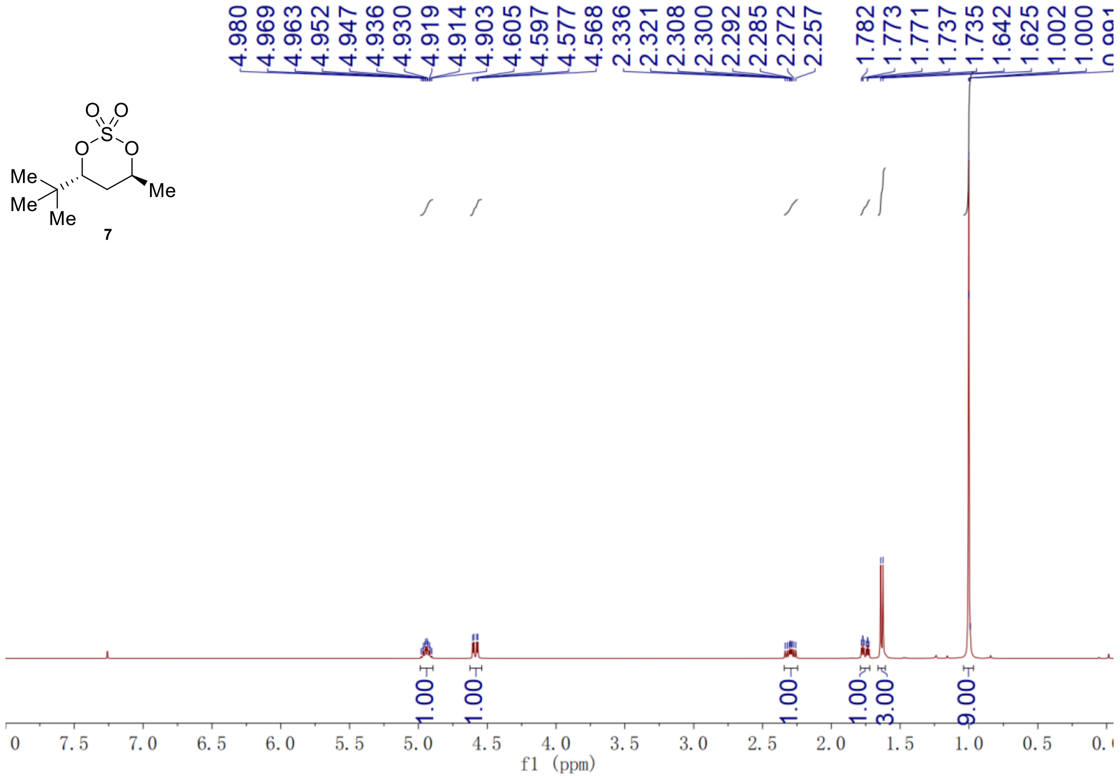

^{13}C NMR Spectrum of **7** (100 MHz, $CDCl_3$)

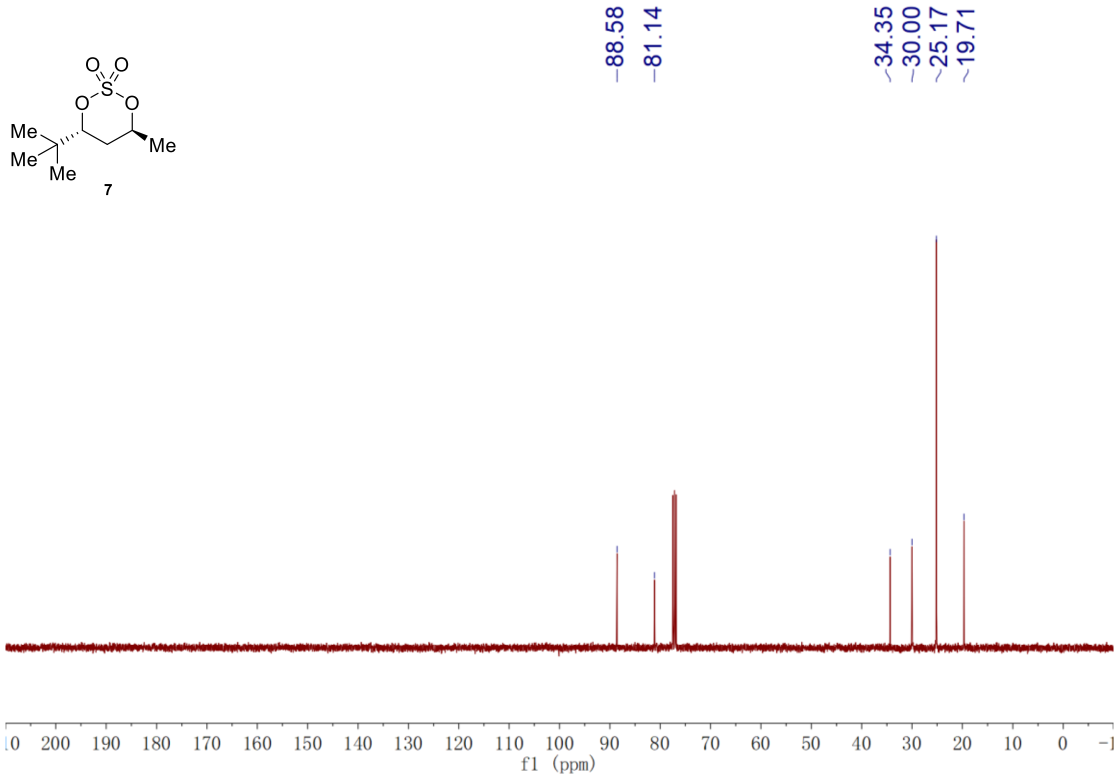

^{1}H NMR Spectrum of **S12** (400 MHz, $CDCl_3$)

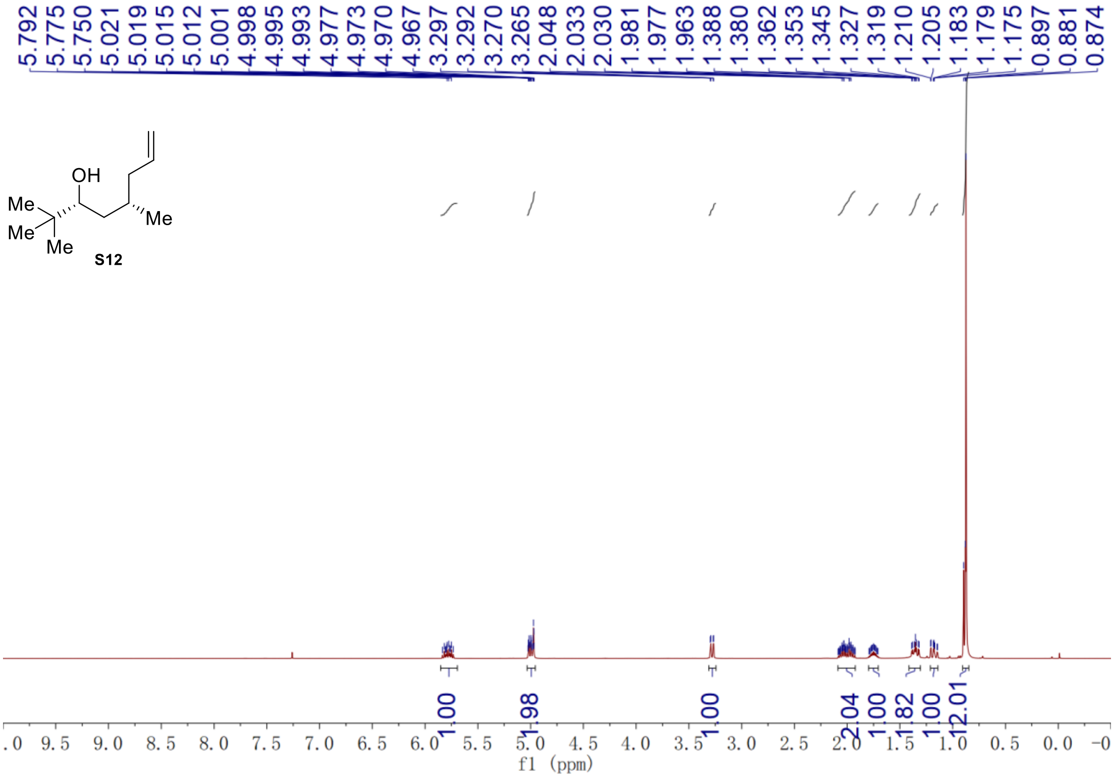

^{13}C NMR Spectrum of **S12** (100 MHz, $CDCl_3$)

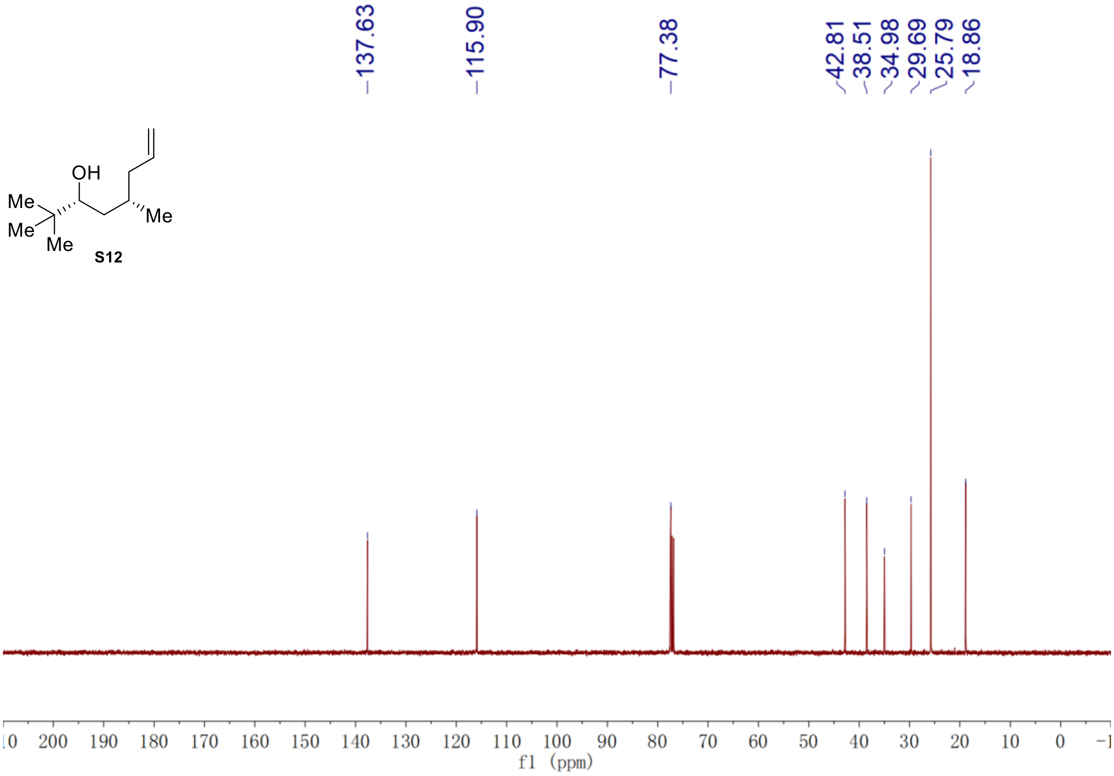

^{1}H NMR Spectrum of **13** (400 MHz, $CDCl_3$)

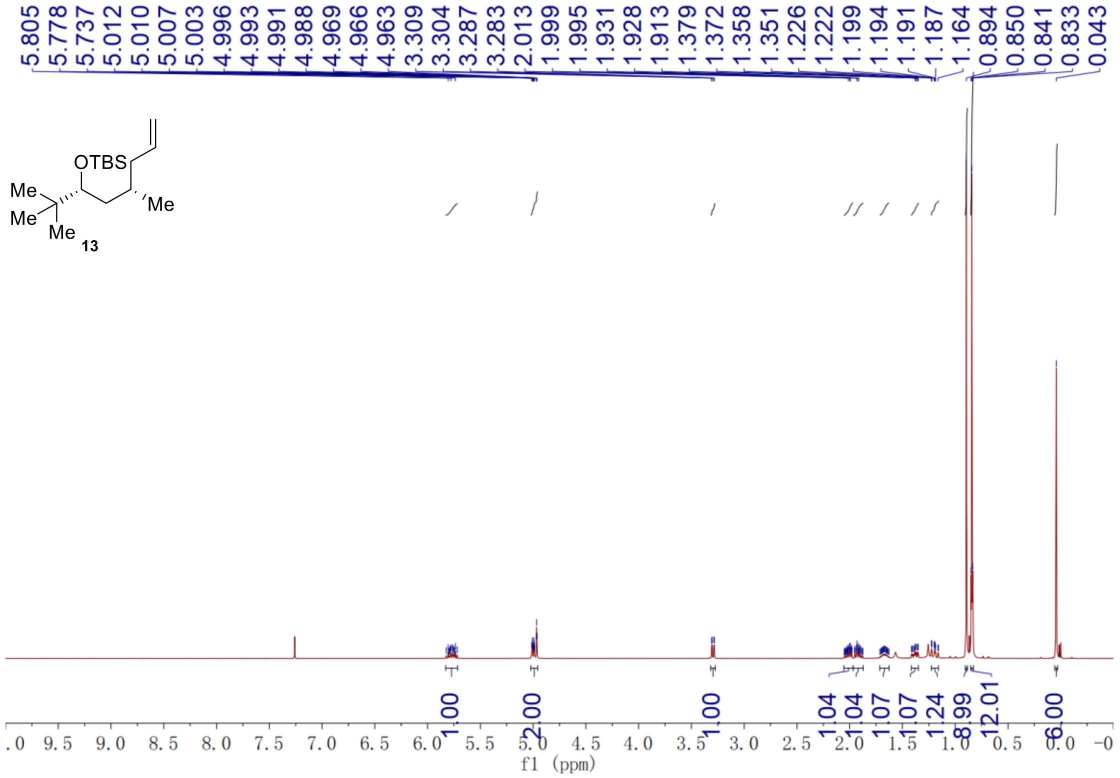

^{13}C NMR Spectrum of **13** (100 MHz, $CDCl_3$)

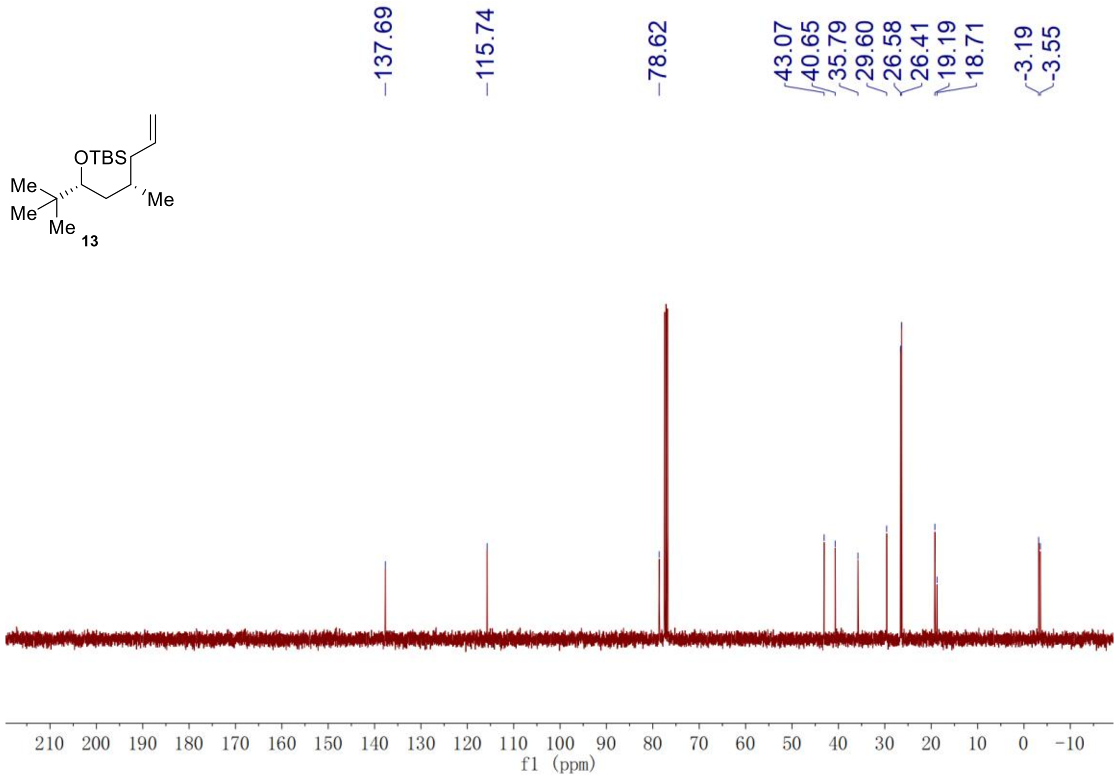

^{1}H NMR Spectrum of **14** (400 MHz, $CDCl_3$)

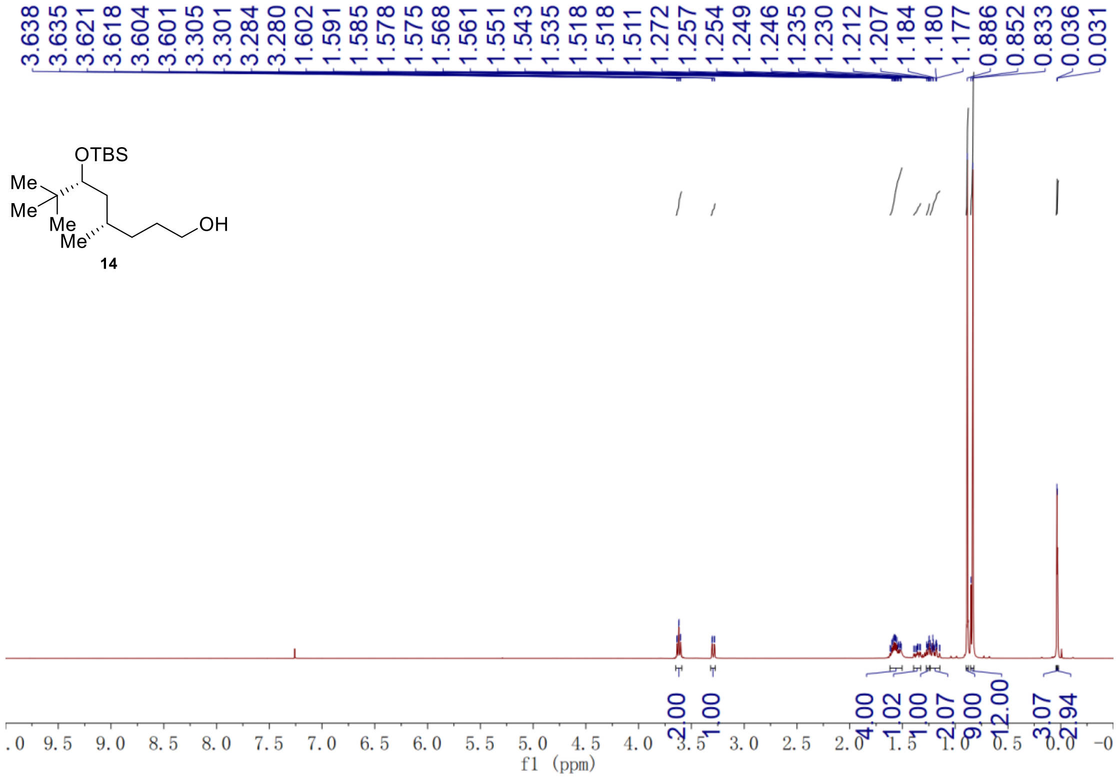

^{13}C NMR Spectrum of **14** (100 MHz, $CDCl_3$)

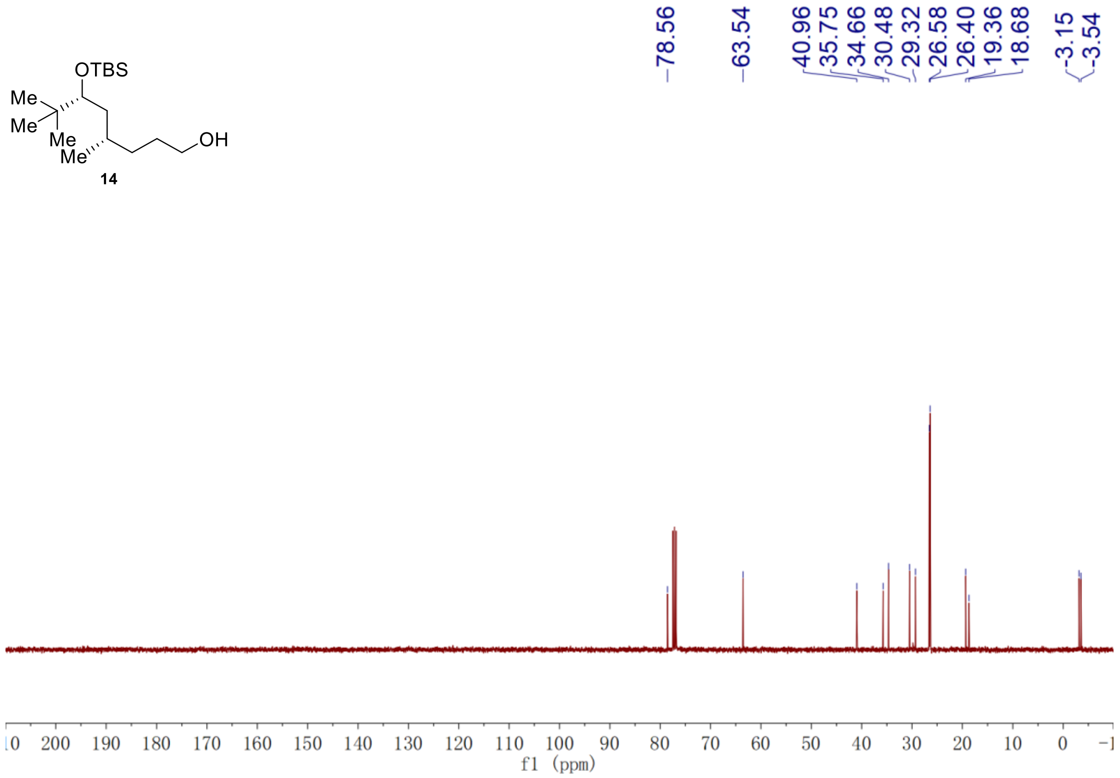

^{1}H NMR Spectrum of **15** (400 MHz, $CDCl_3$)

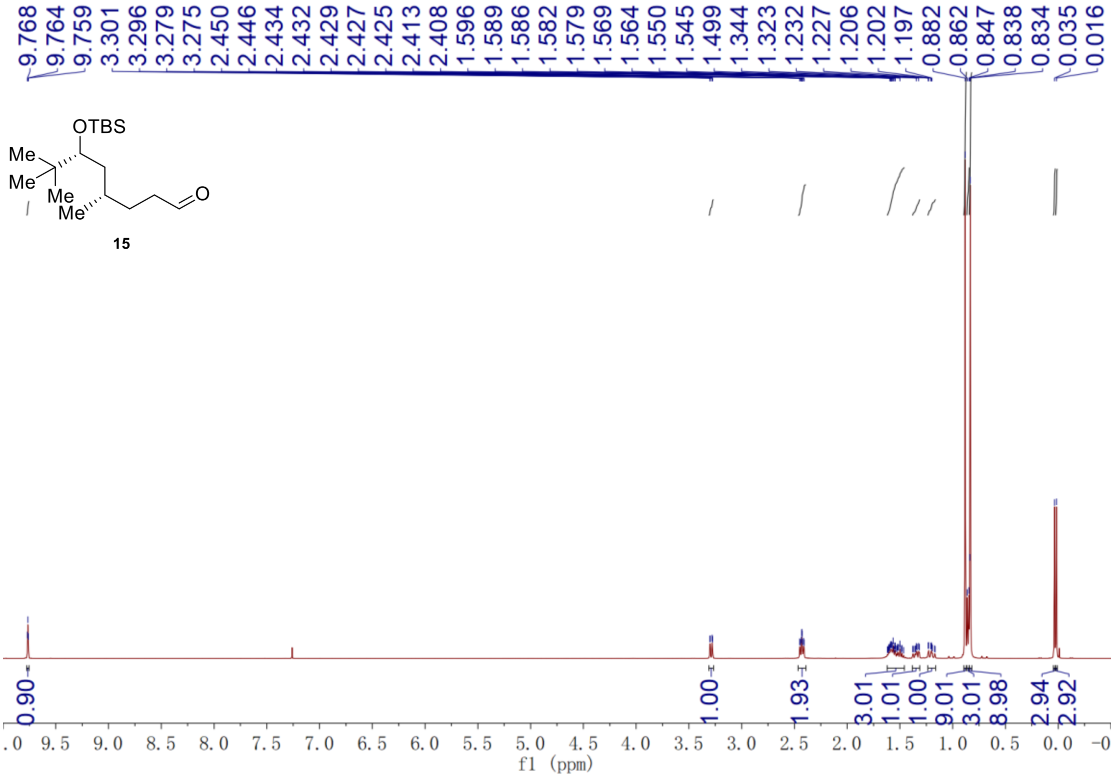

^{13}C NMR Spectrum of **15** (100 MHz, $CDCl_3$)

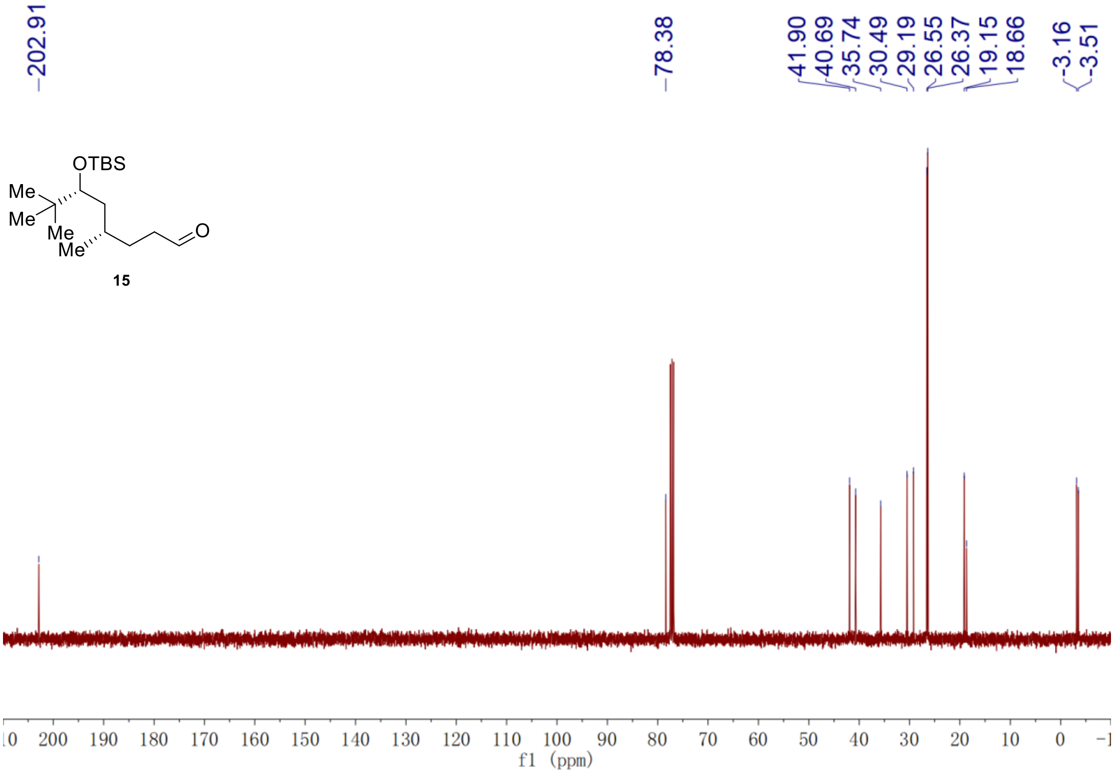

^{1}H NMR Spectrum of **16** (400 MHz, $CDCl_3$)

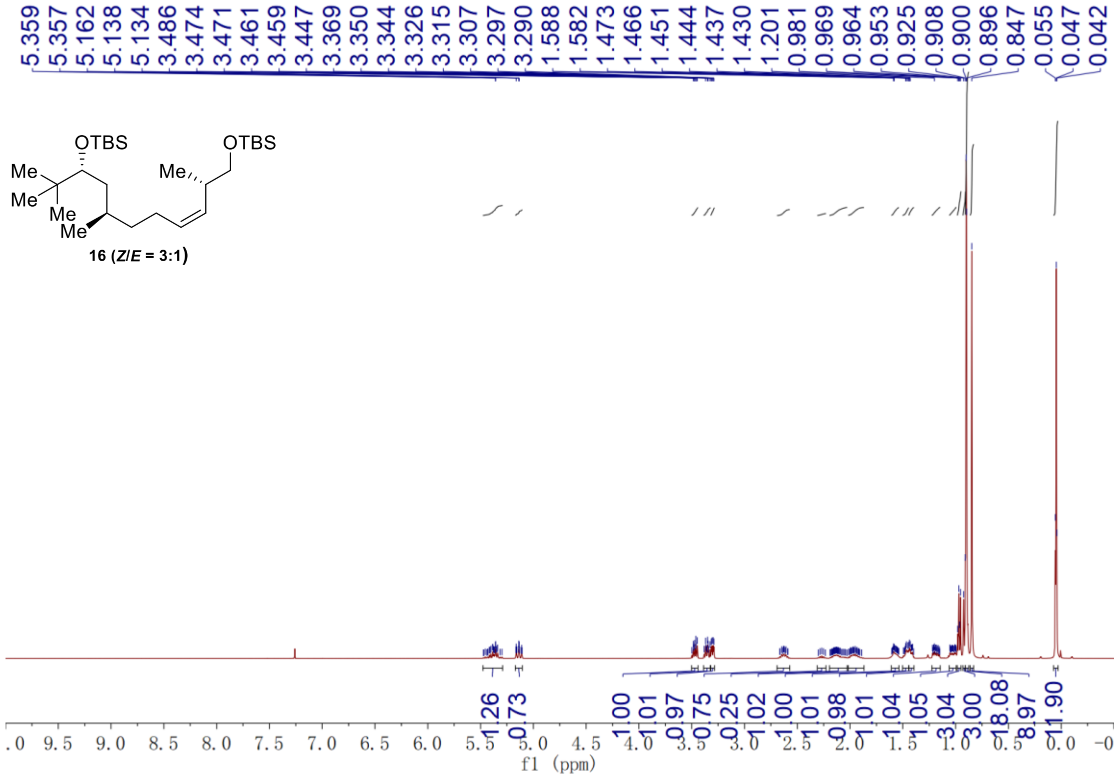

^{13}C NMR Spectrum of **16** (100 MHz, $CDCl_3$)

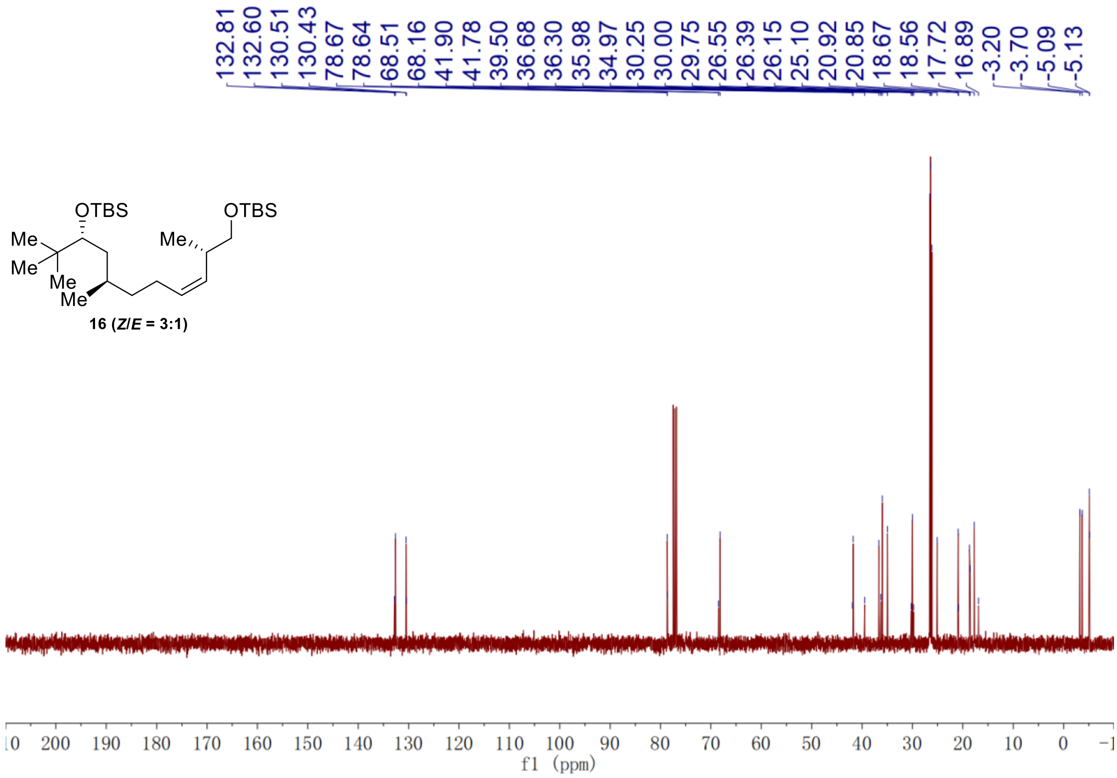

^{1}H NMR Spectrum of **17** (500 MHz, $CDCl_3$)

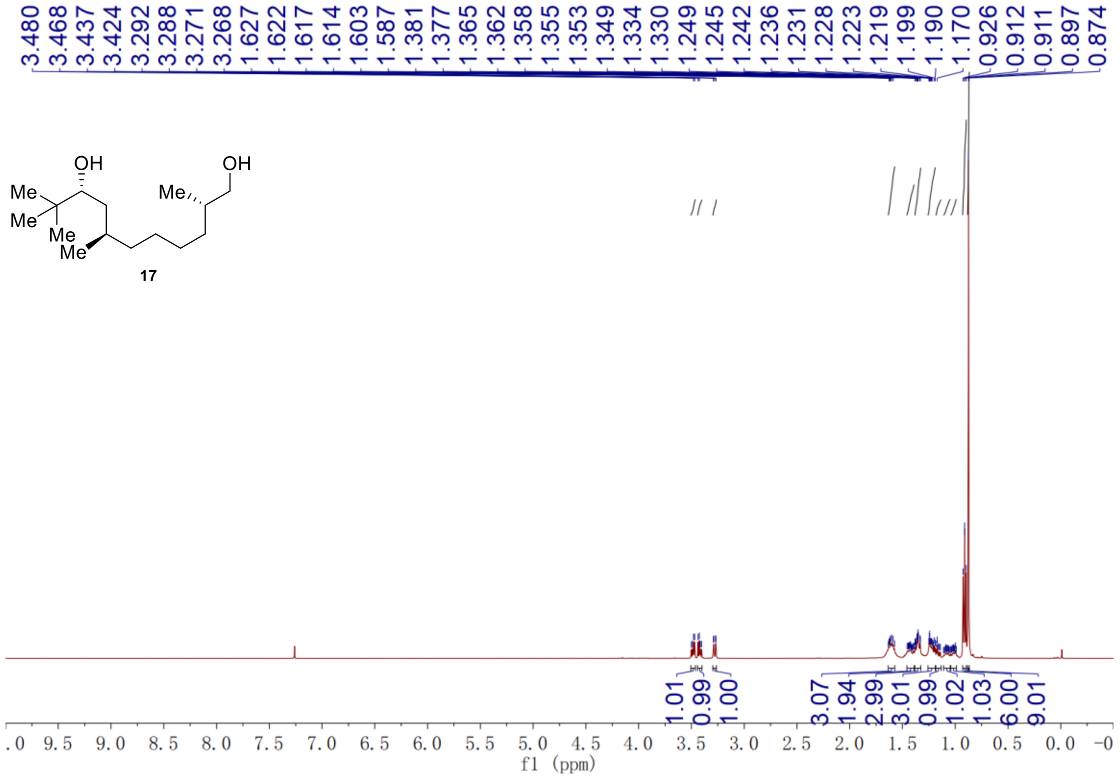

^{13}C NMR Spectrum of **17** (125 MHz, $CDCl_3$)

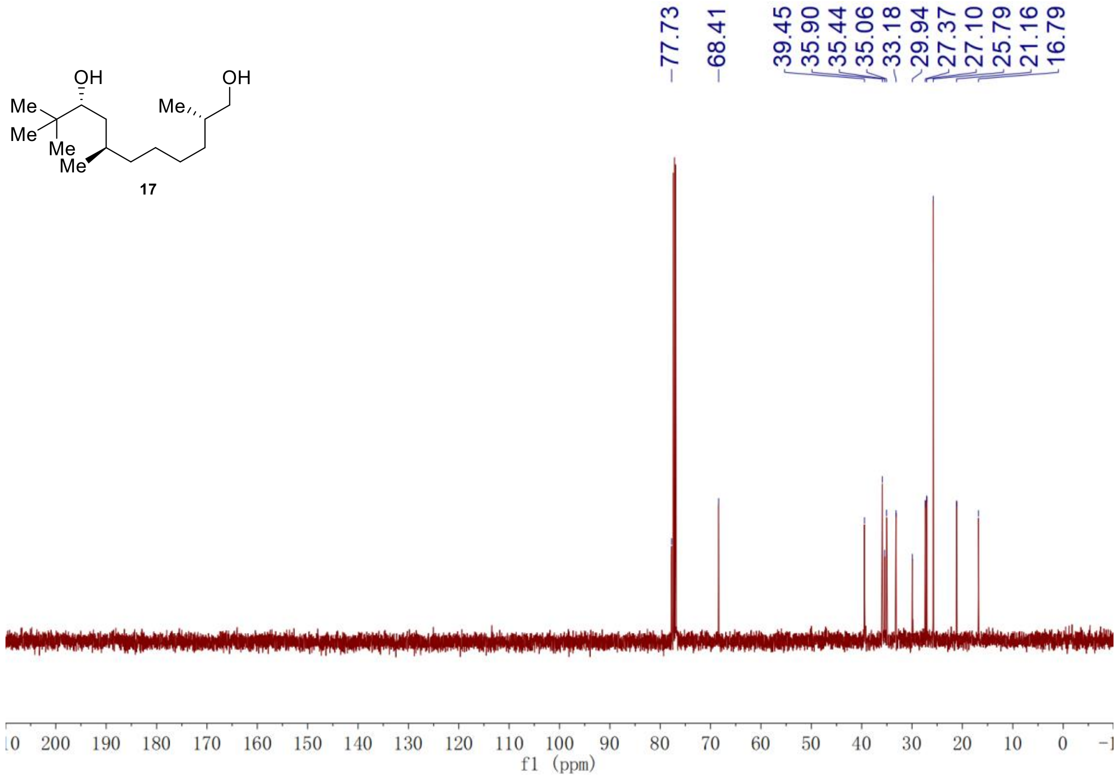

^{1}H NMR Spectrum of **S14** (400 MHz, $CDCl_3$)

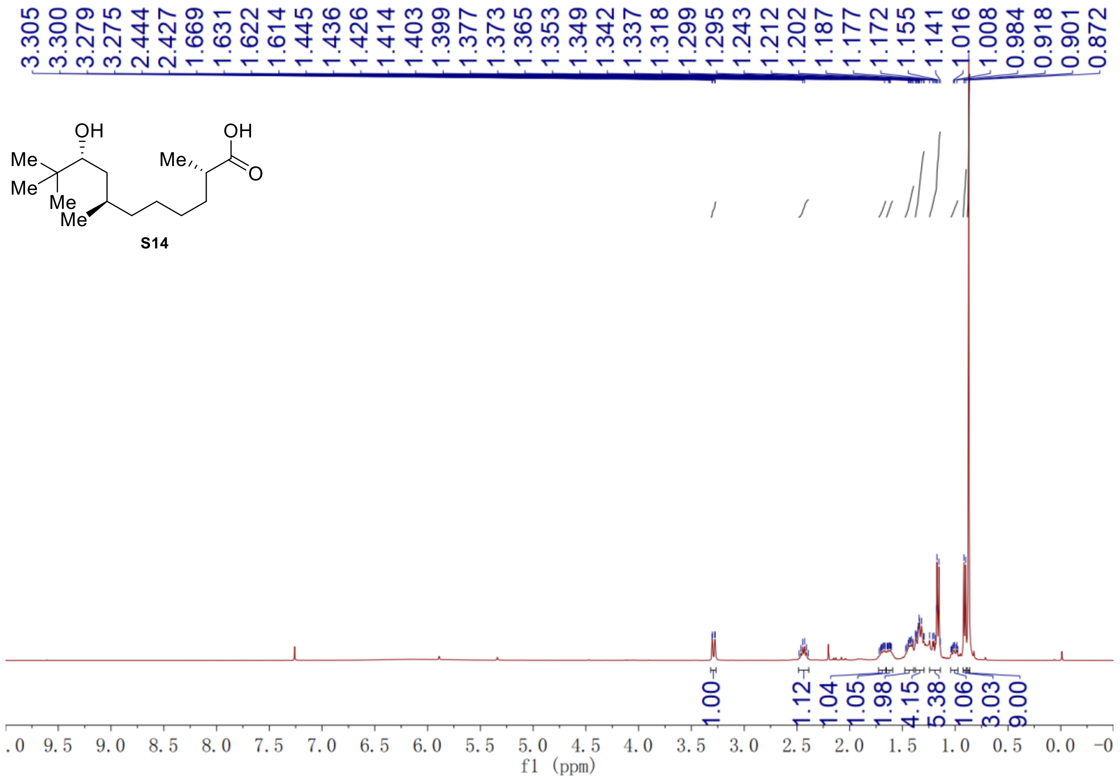

^{13}C NMR Spectrum of **S14** (100 MHz, $CDCl_3$)

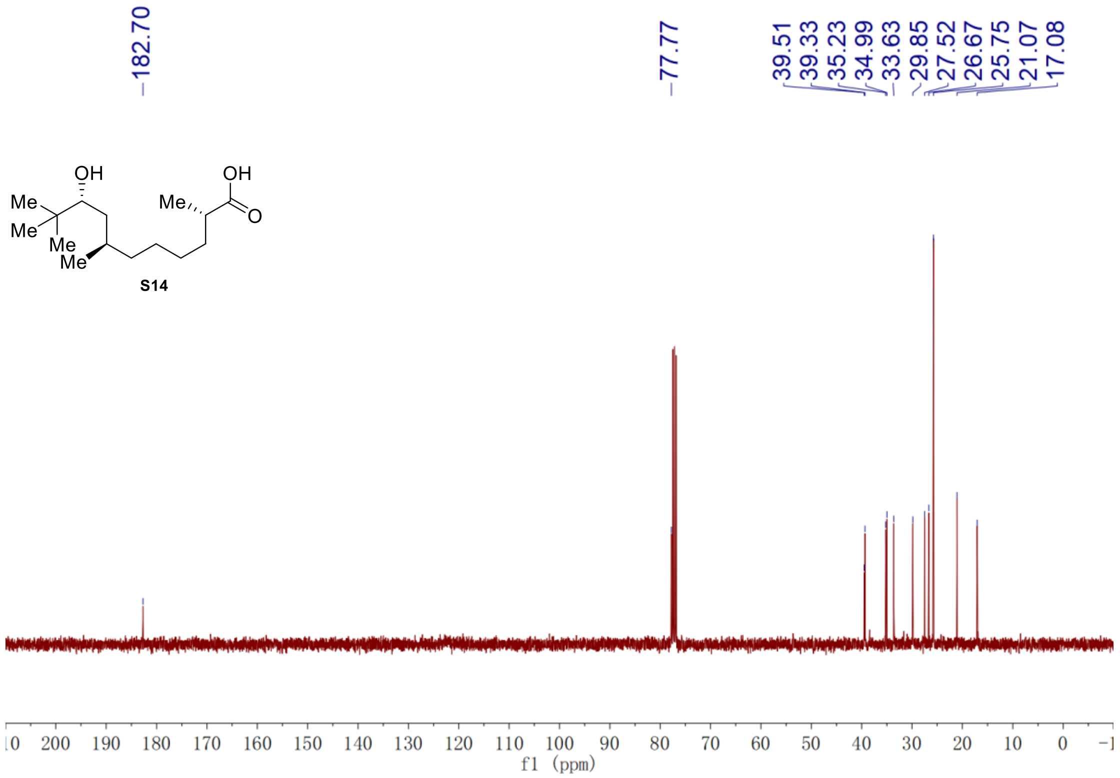

^{1}H NMR Spectrum of **18** (400 MHz, $CDCl_3$)

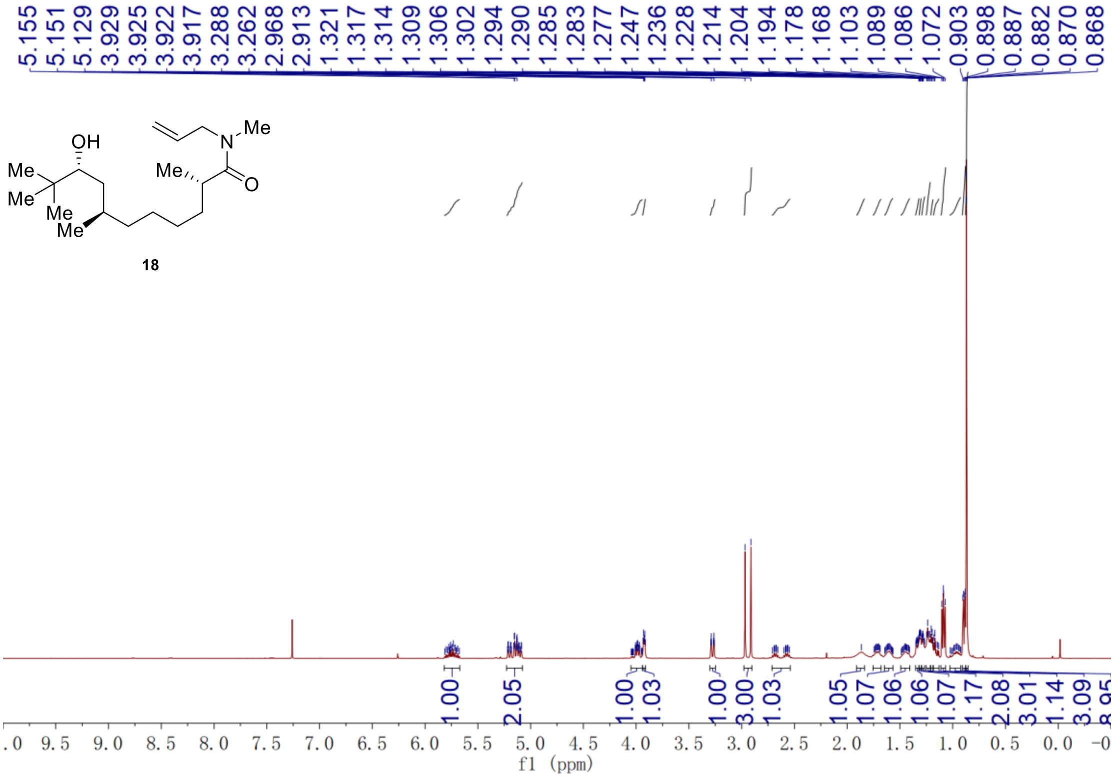

^{13}C NMR Spectrum of **18** (100 MHz, $CDCl_3$)

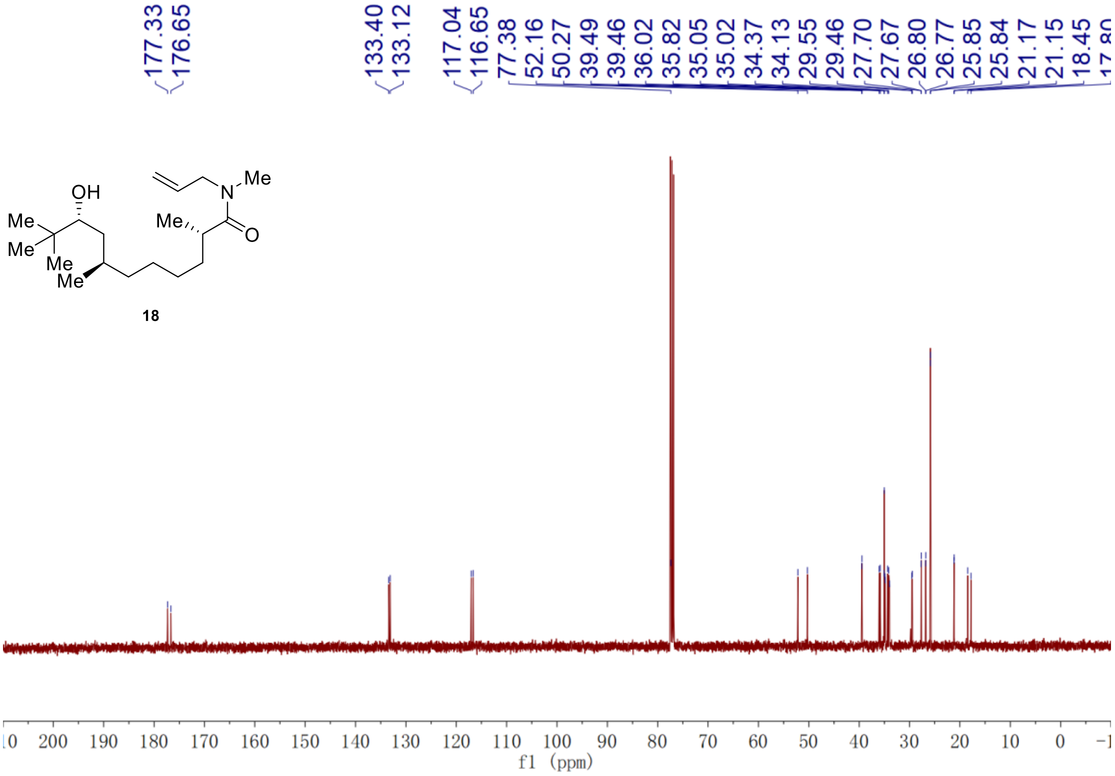

^{1}H NMR Spectrum of **19** (400 MHz, $CDCl_3$)

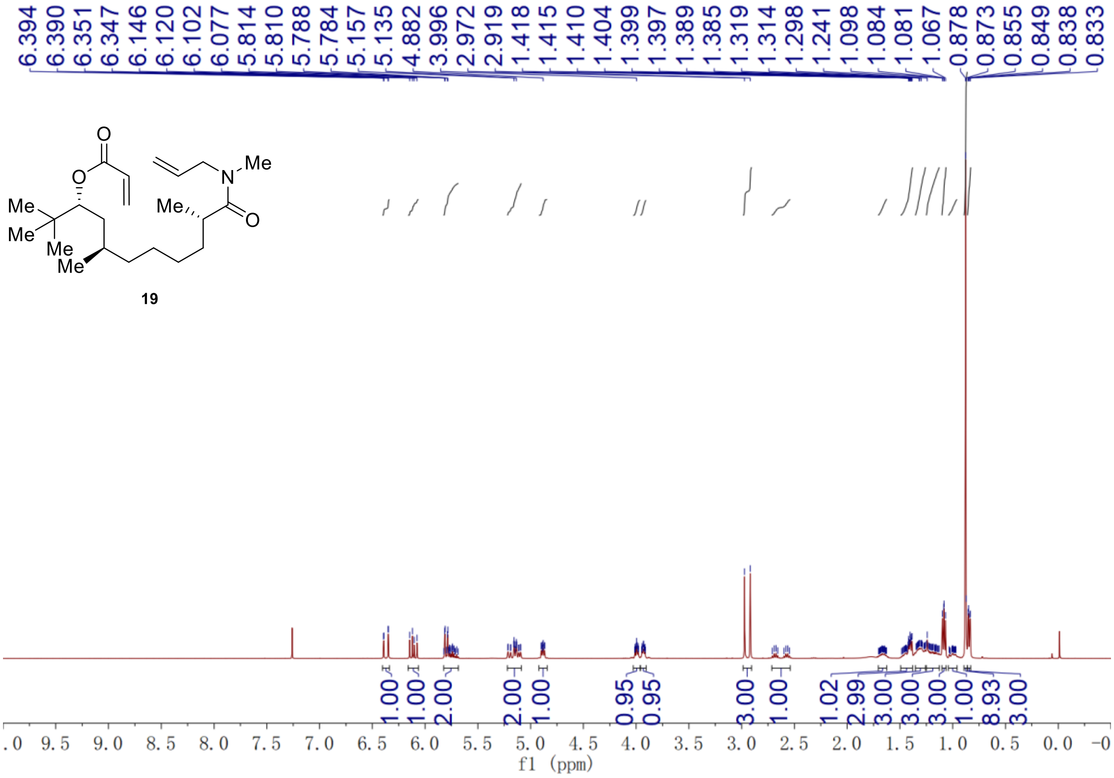

^{13}C NMR Spectrum of **19** (100 MHz, $CDCl_3$)

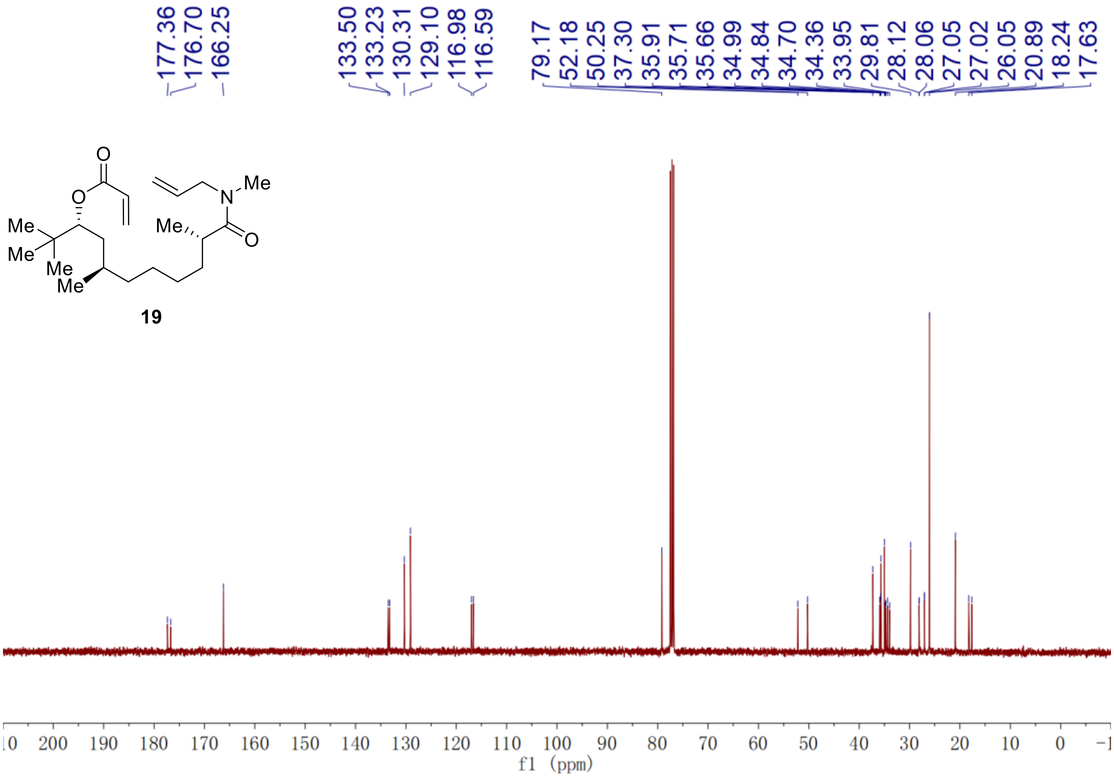

¹H NMR Spectrum of (2*S*,7*R*,9*R*)-laingolide A **(1a)** (400 MHz, $CDCl_3$)

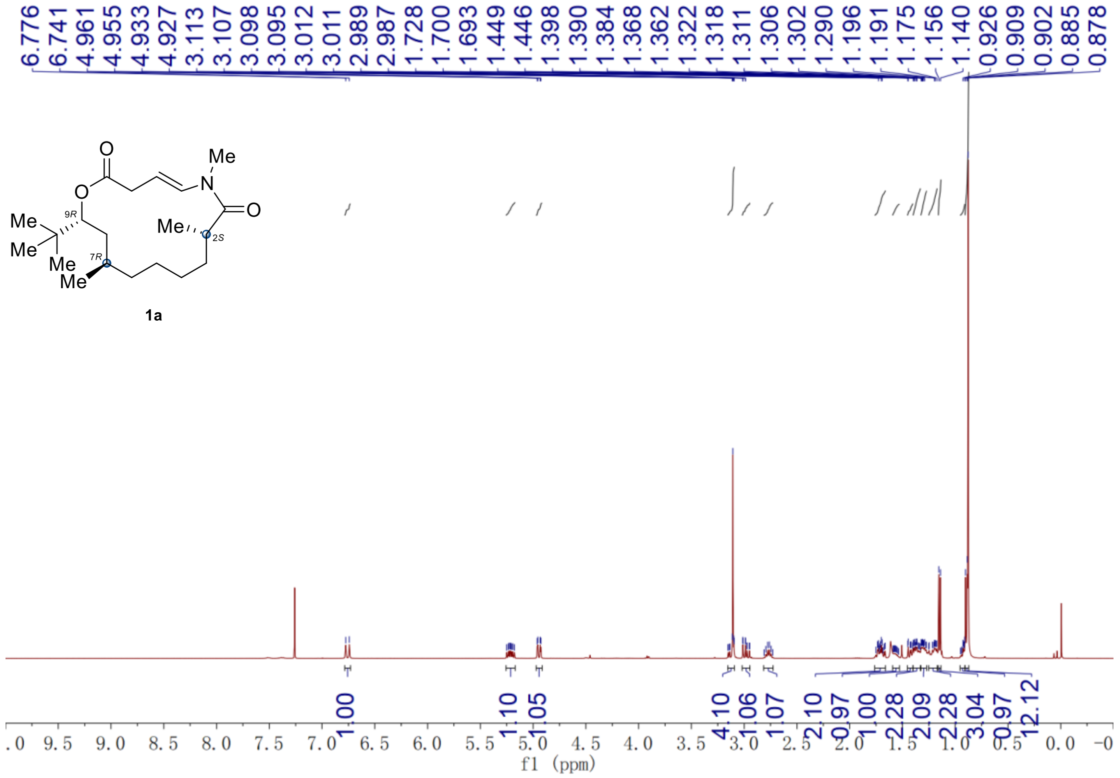

¹³C NMR Spectrum of (2*S*,7*R*,9*R*)-laingolide A **(1a)** (100 MHz, $CDCl_3$)

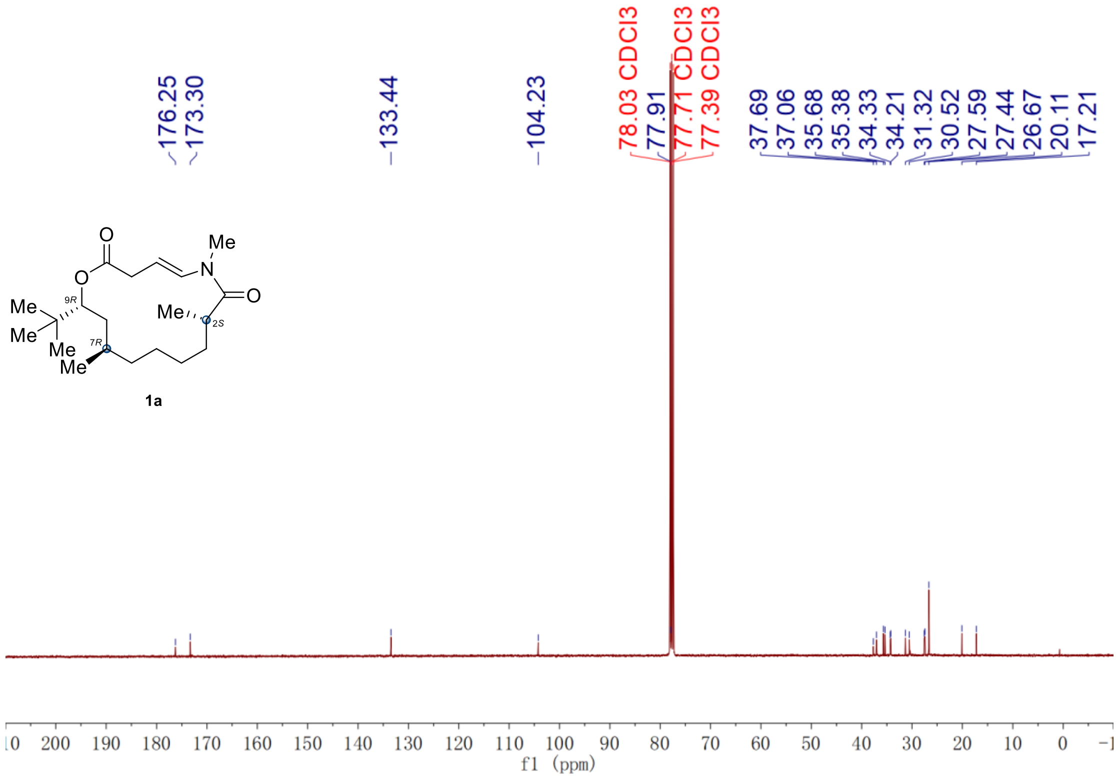

^{1}H NMR Spectrum of **20** (400 MHz, $CDCl_3$)

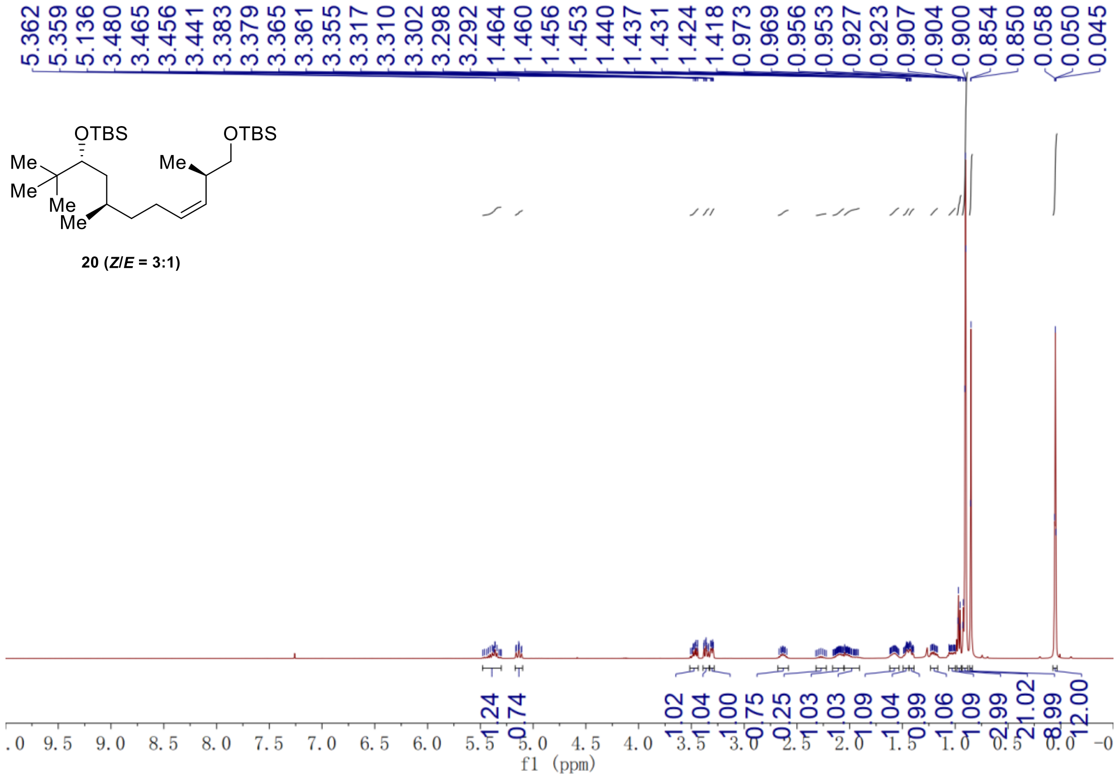

^{13}C NMR Spectrum of **20** (100 MHz, $CDCl_3$)

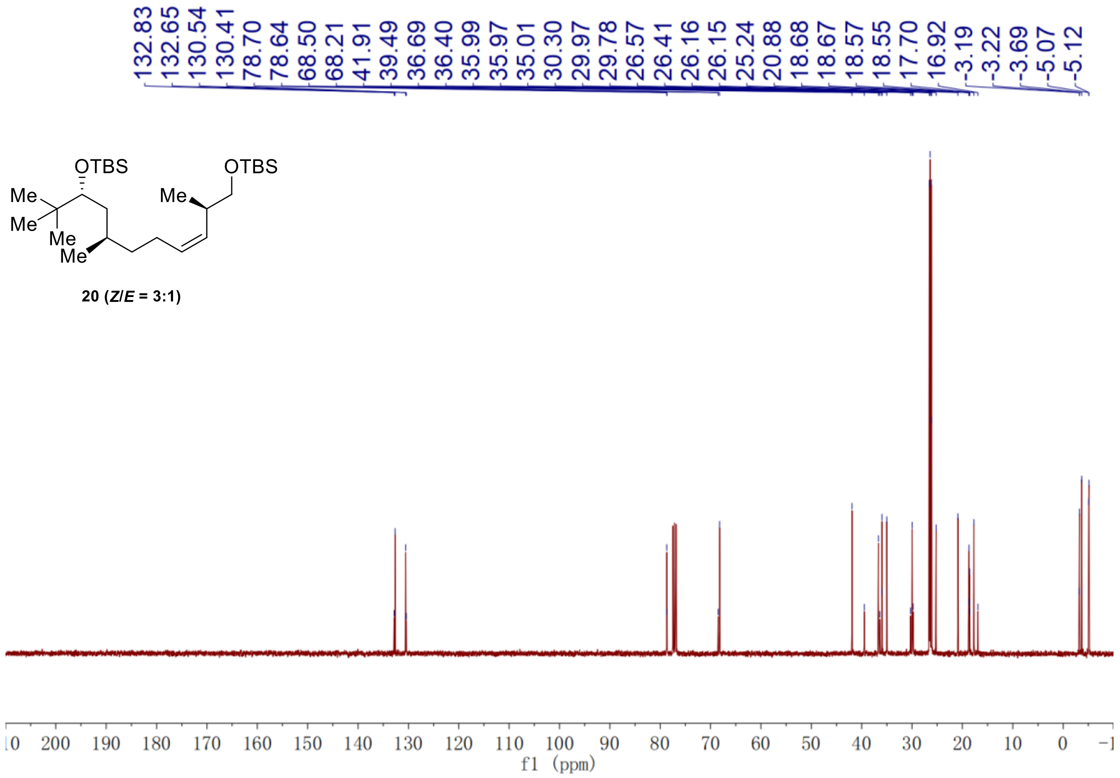

^{1}H NMR Spectrum of **21** (500 MHz, $CDCl_3$)

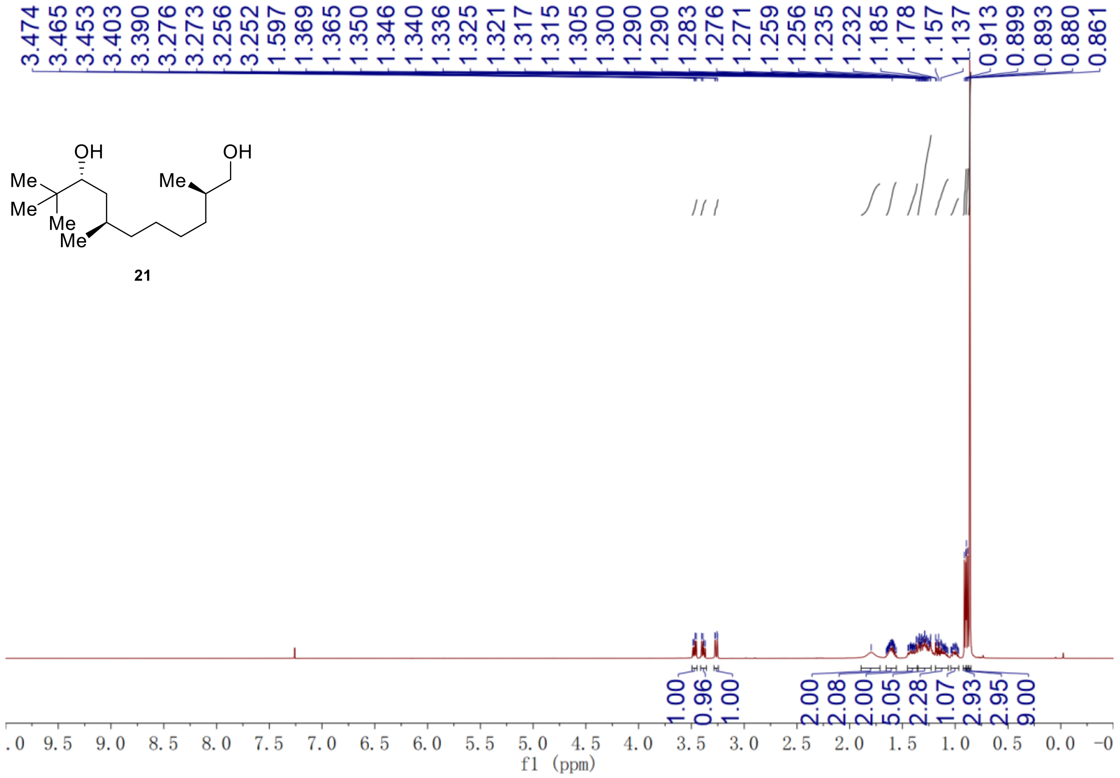

^{13}C NMR Spectrum of **21** (125 MHz, $CDCl_3$)

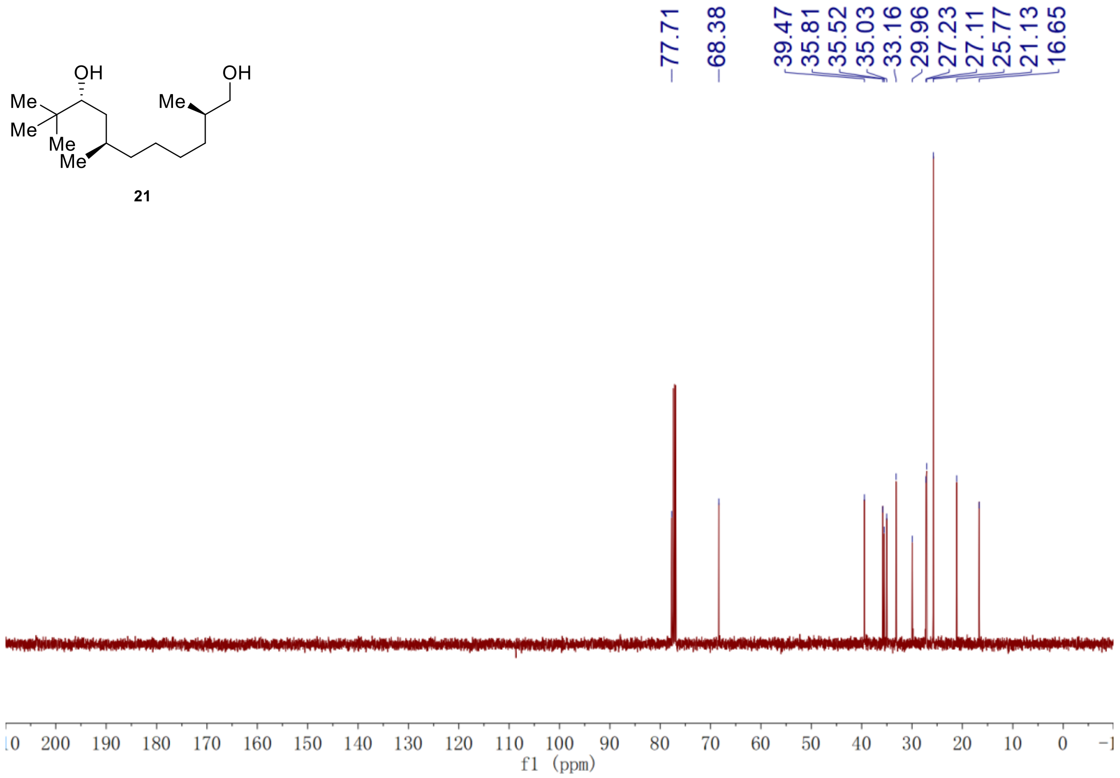

^1H NMR Spectrum of **S16** (400 MHz, $CDCl_3$)

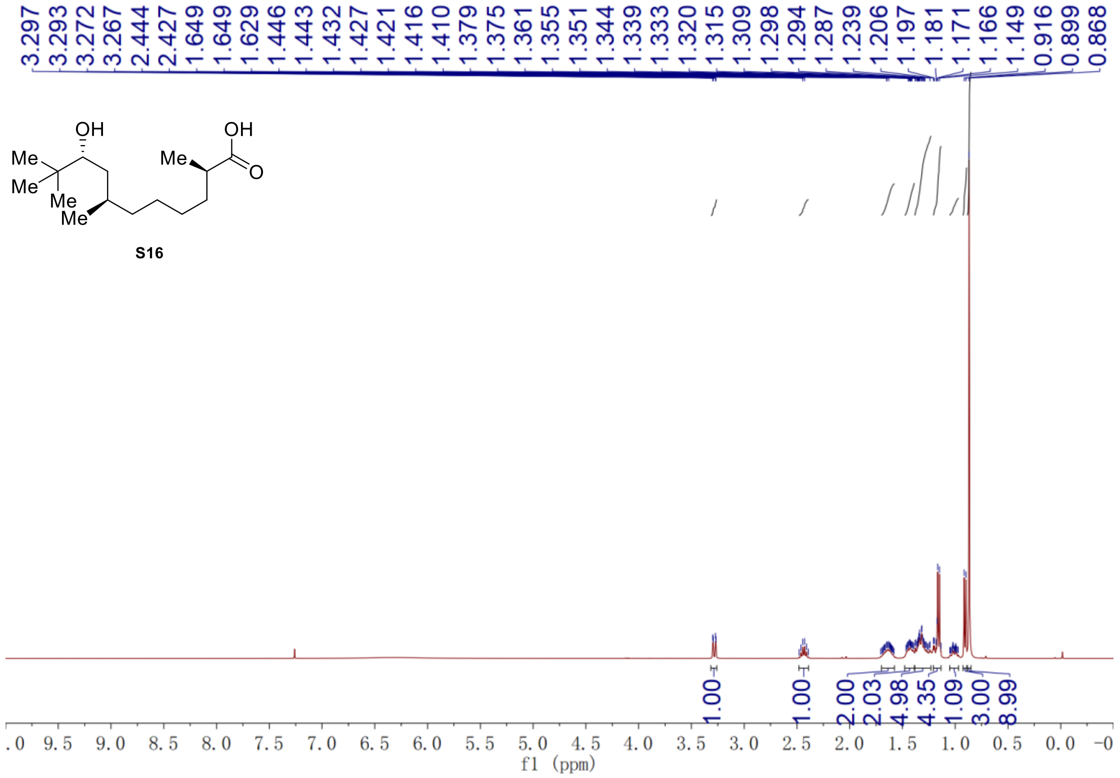

^{13}C NMR Spectrum of **S16** (100 MHz, $CDCl_3$)

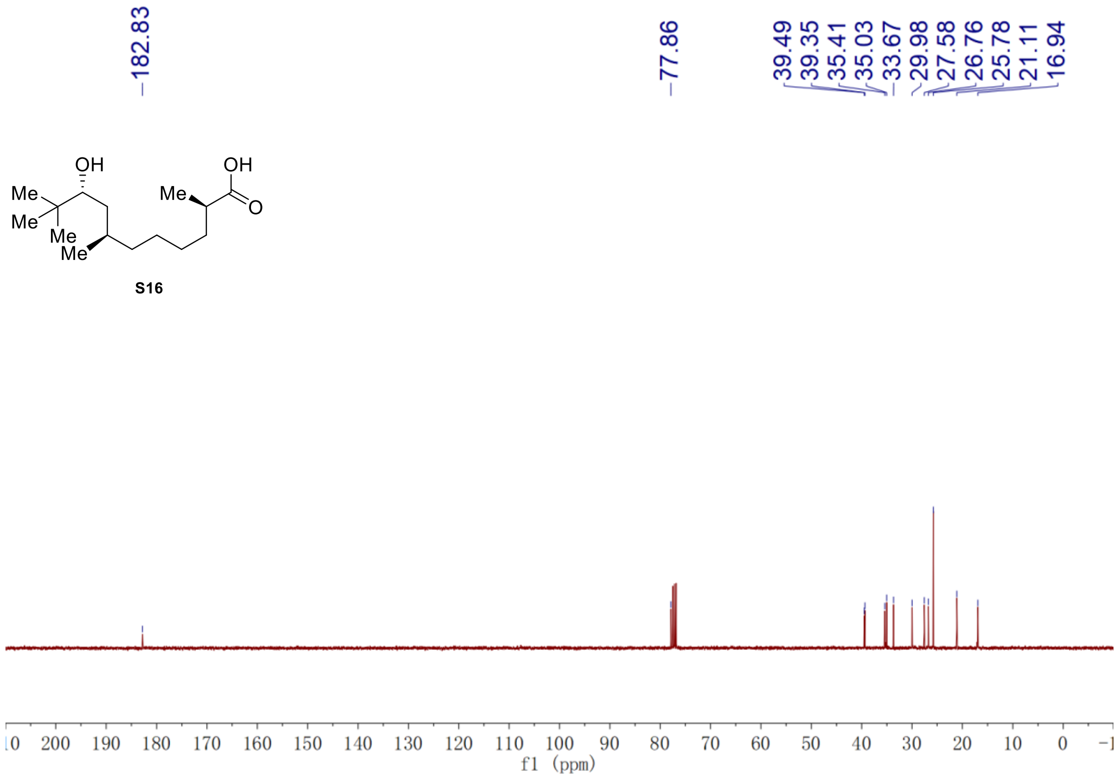

^{1}H NMR Spectrum of **22** (400 MHz, $CDCl_3$)

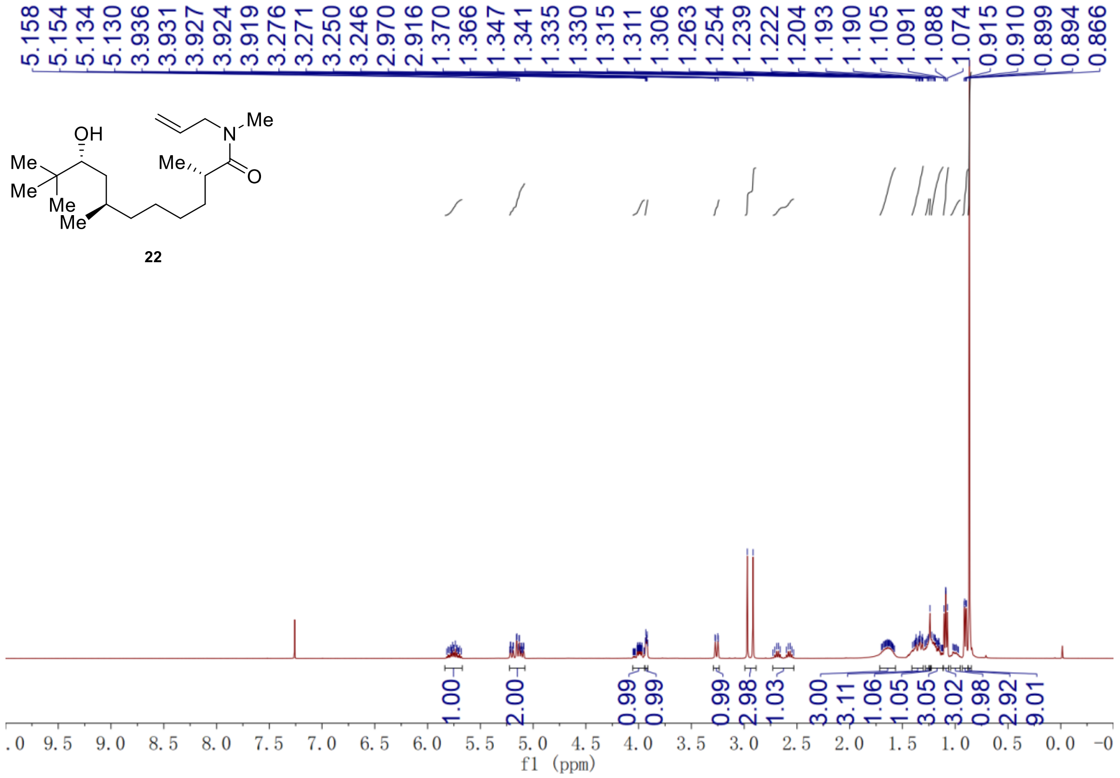

^{13}C NMR Spectrum of **22** (100 MHz, $CDCl_3$)

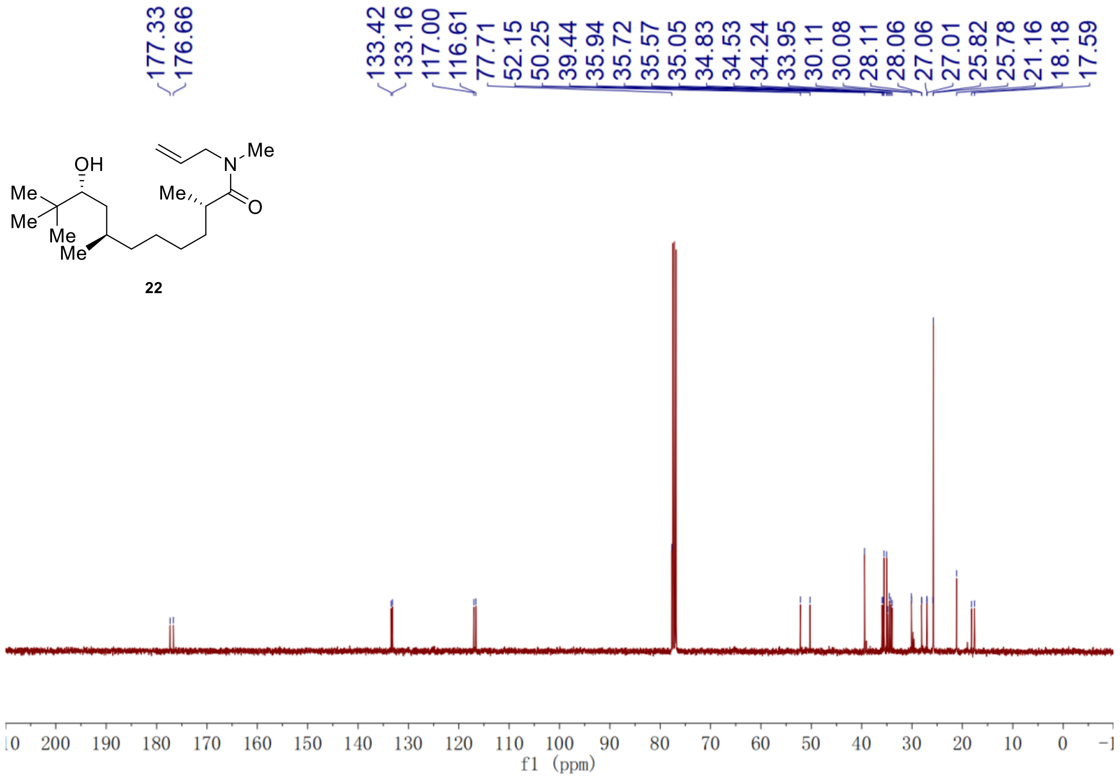

^{1}H NMR Spectrum of **23** (400 MHz, $CDCl_3$)

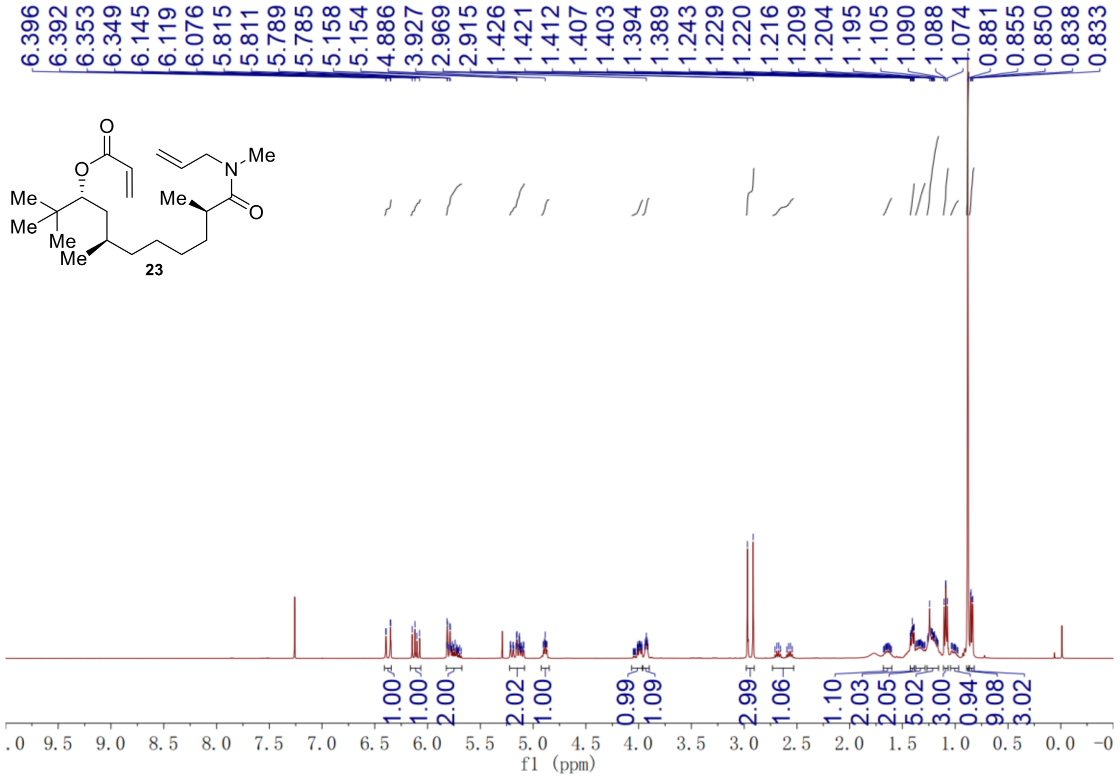

^{13}C NMR Spectrum of **23** (100 MHz, $CDCl_3$)

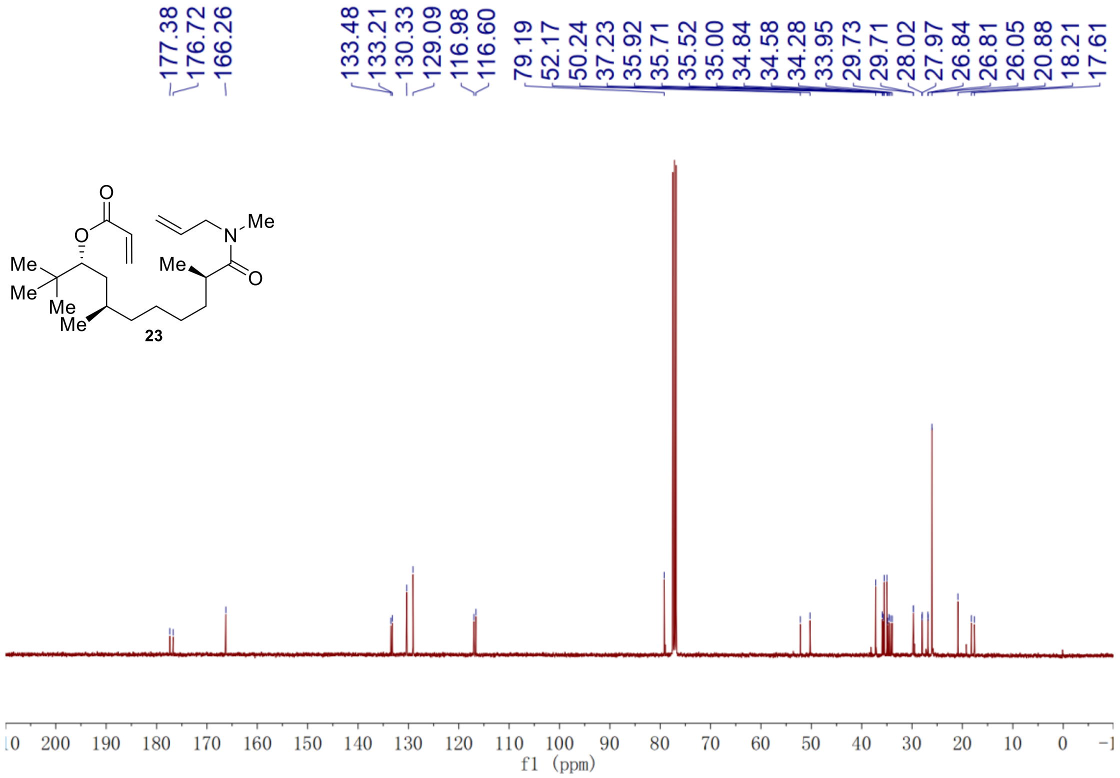

^{1}H NMR Spectrum of (2*R*,7*R*,9*R*)-laingolide A **(1b)** (500 MHz, $CDCl_3$)

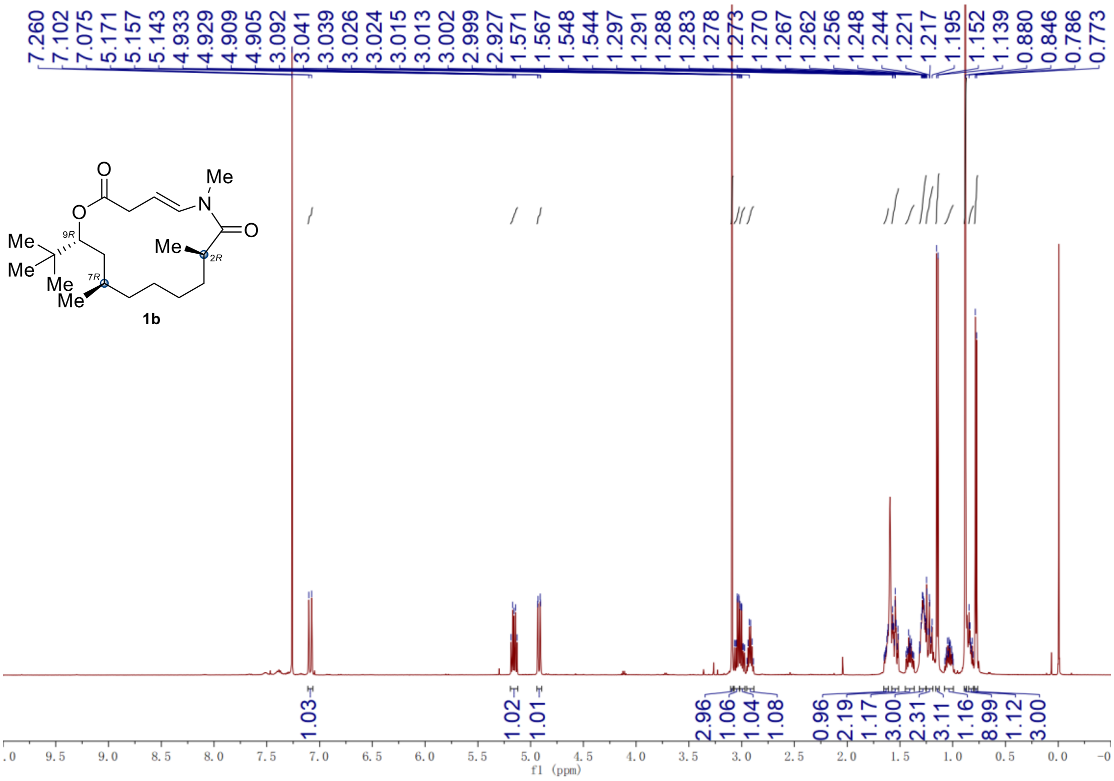

^{13}C NMR Spectrum of (2*R*,7*R*,9*R*)-laingolide A **(1b)** (125 MHz, $CDCl_3$)

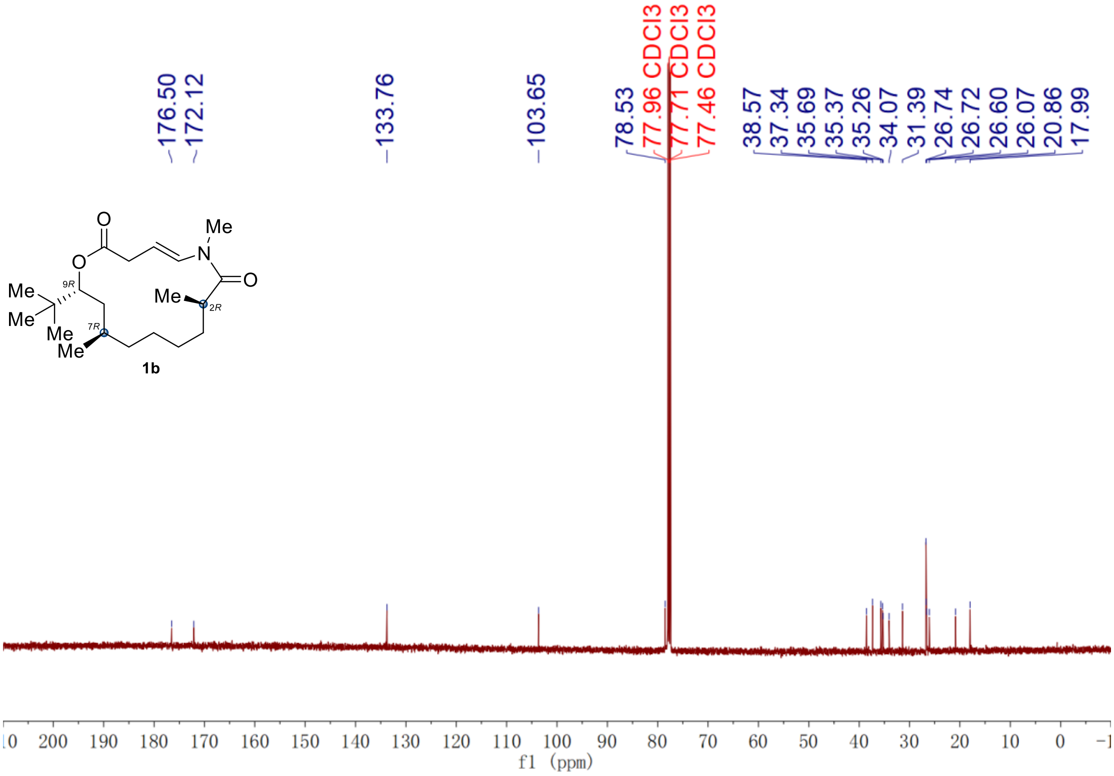

^{1}H NMR Spectrum of **24** (400 MHz, $CDCl_3$)

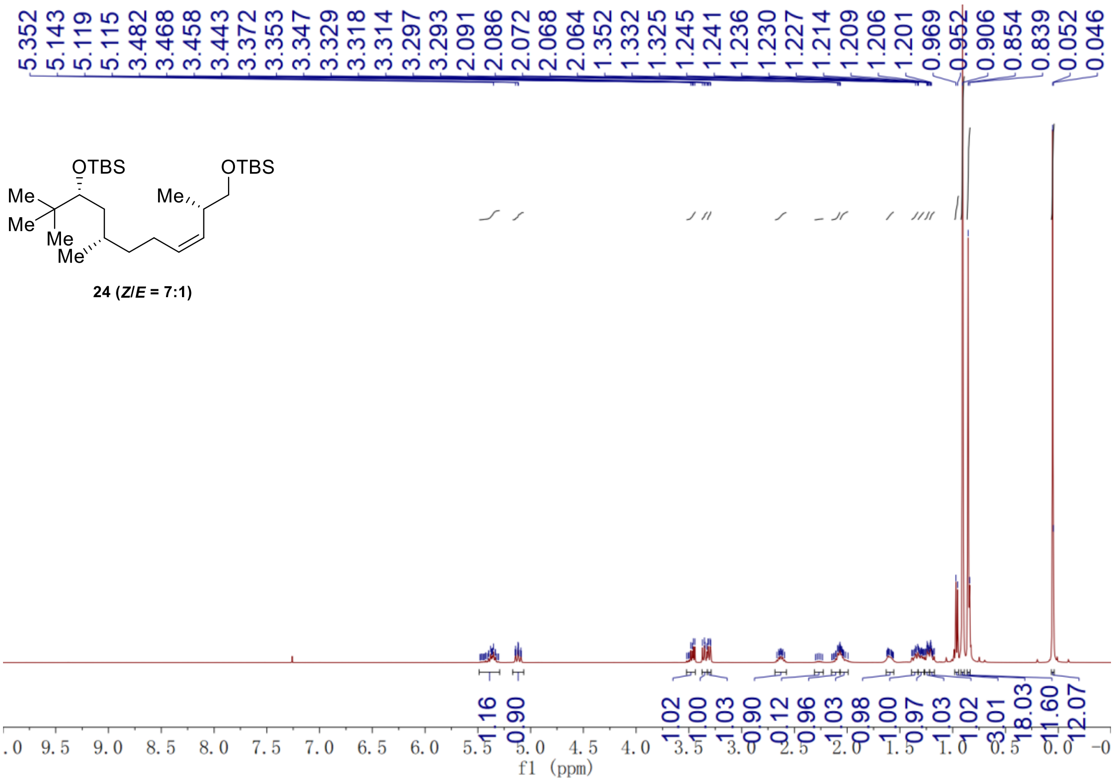

^{13}C NMR Spectrum of **24** (100 MHz, $CDCl_3$)

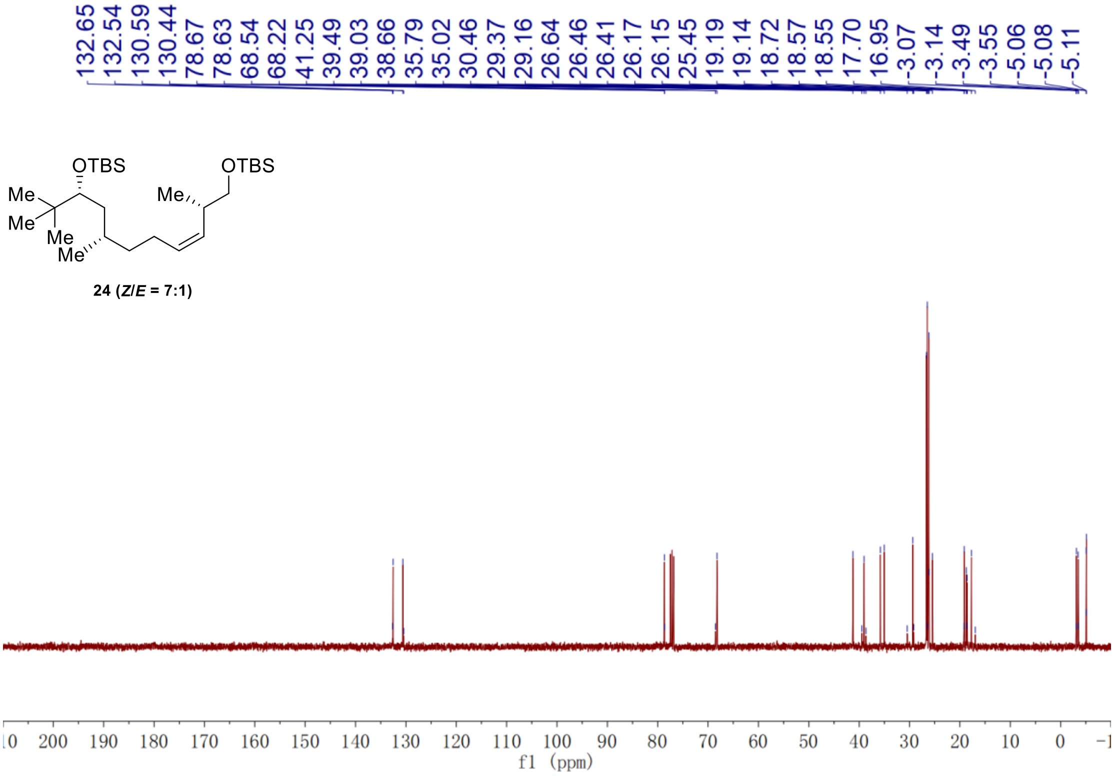

^{1}H NMR Spectrum of **25** (400 MHz, $CDCl_3$)

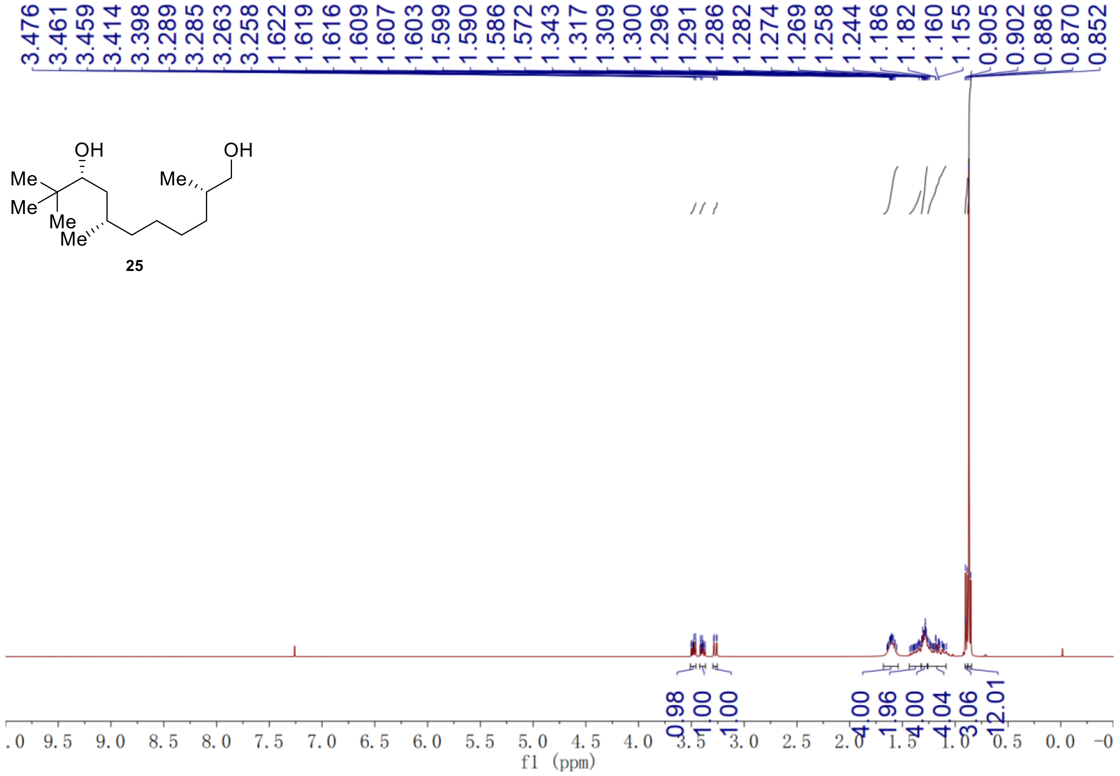

^{13}C NMR Spectrum of **25** (100 MHz, $CDCl_3$)

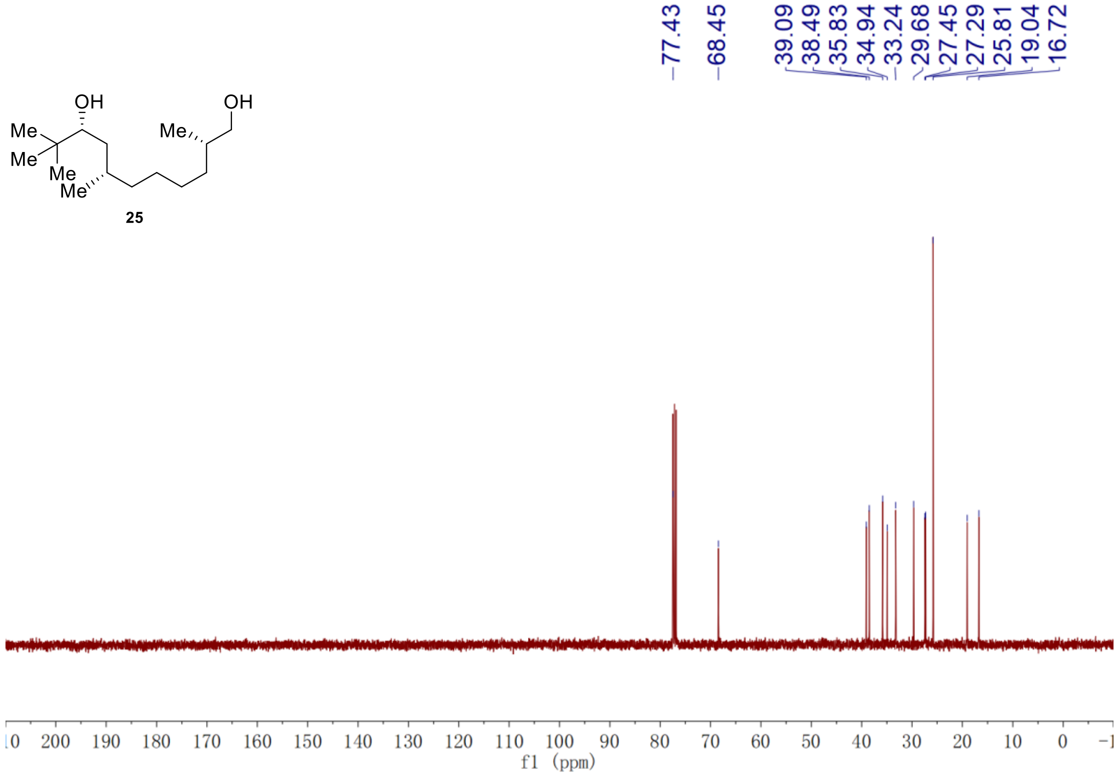

^{1}H NMR Spectrum of **S18** (500 MHz, $CDCl_3$)

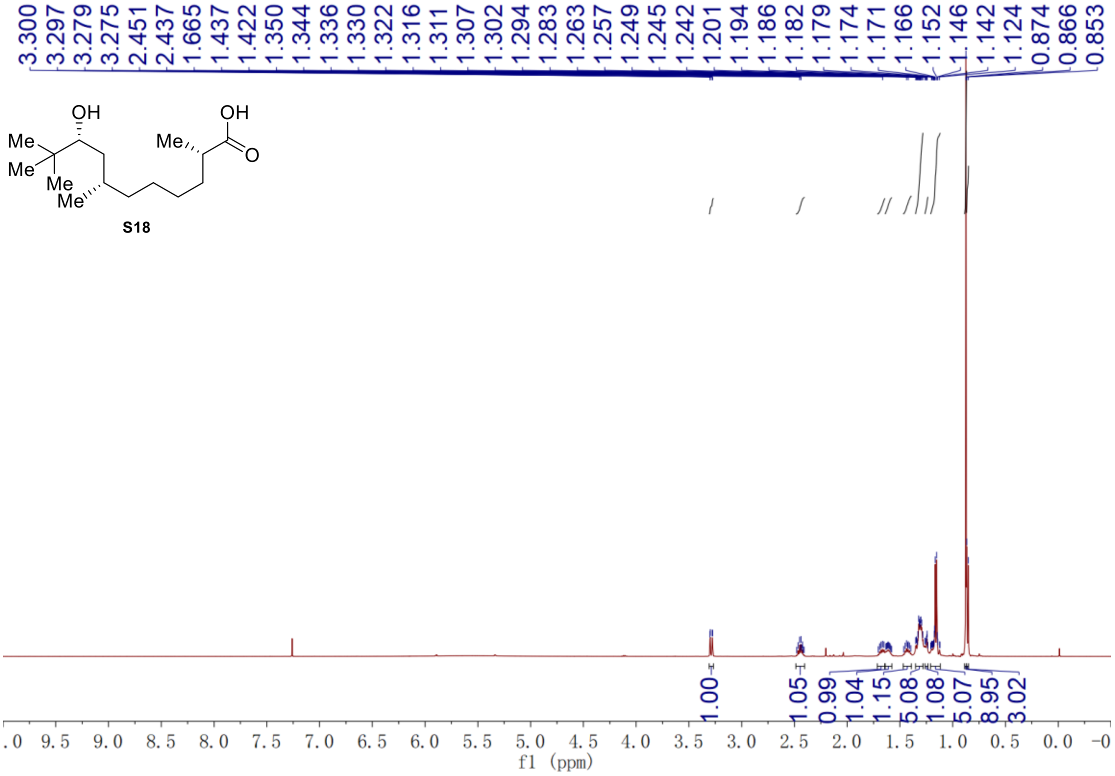

^{13}C NMR Spectrum of **S18** (125 MHz, $CDCl_3$)

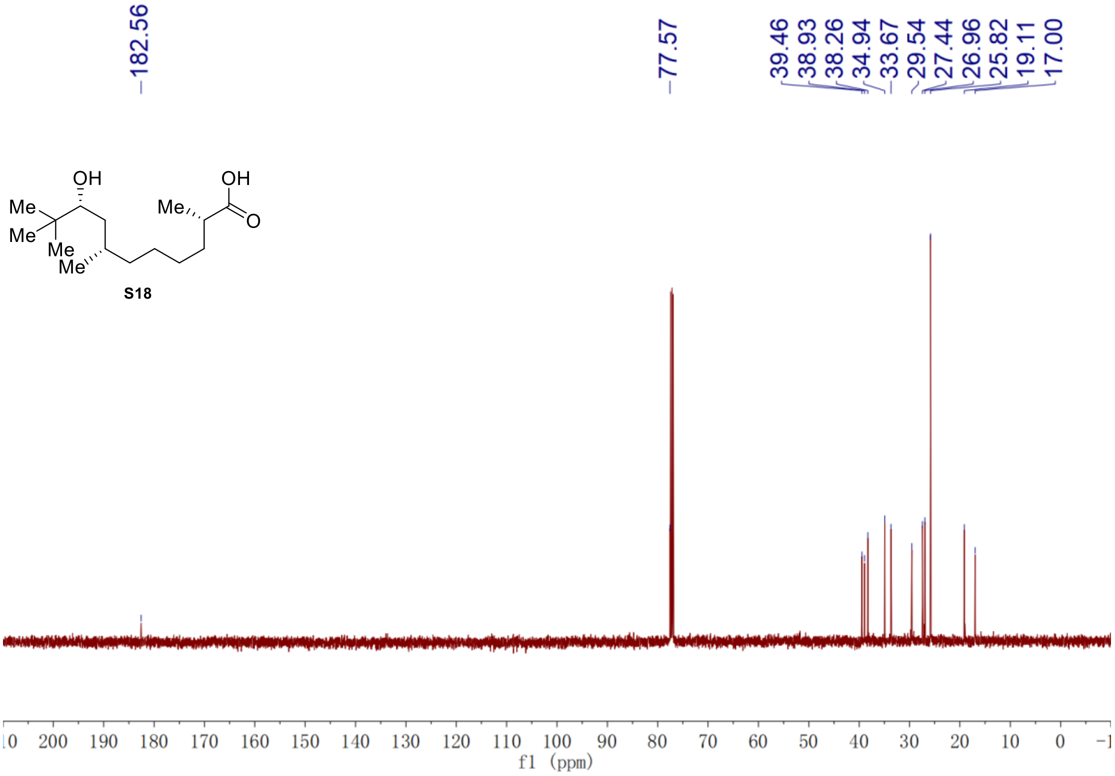

^{1}H NMR Spectrum of **26** (500 MHz, $CDCl_3$)

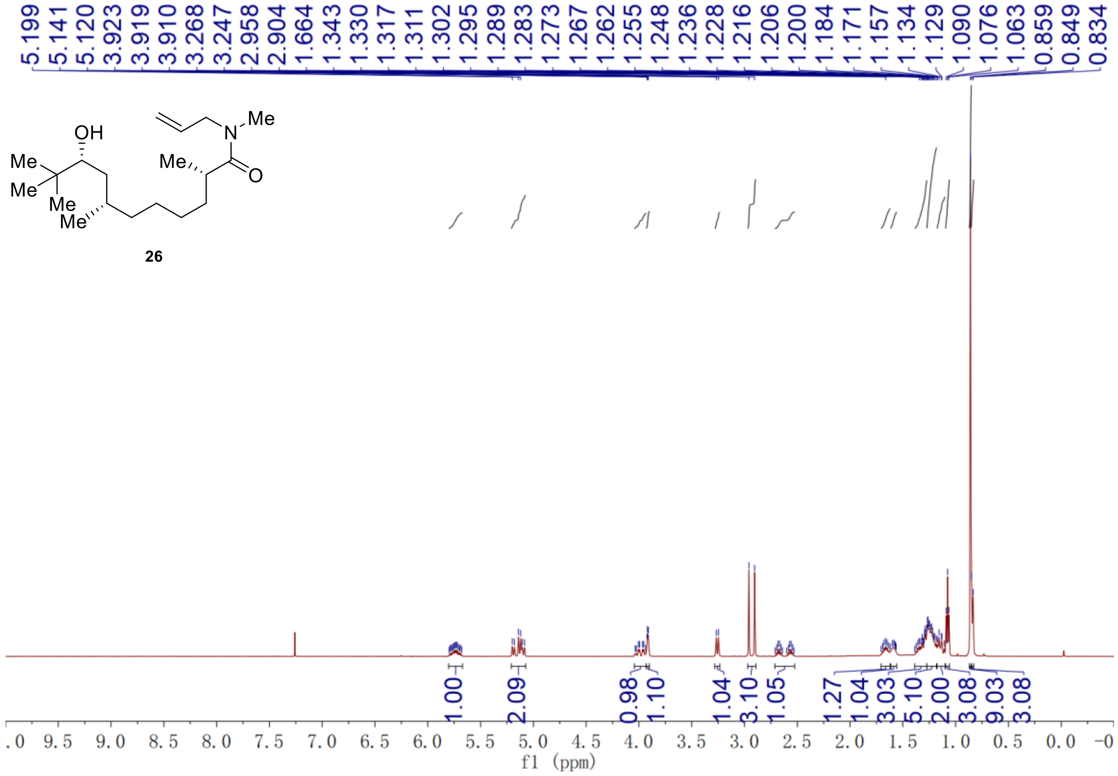

^{13}C NMR Spectrum of **26** (125 MHz, $CDCl_3$)

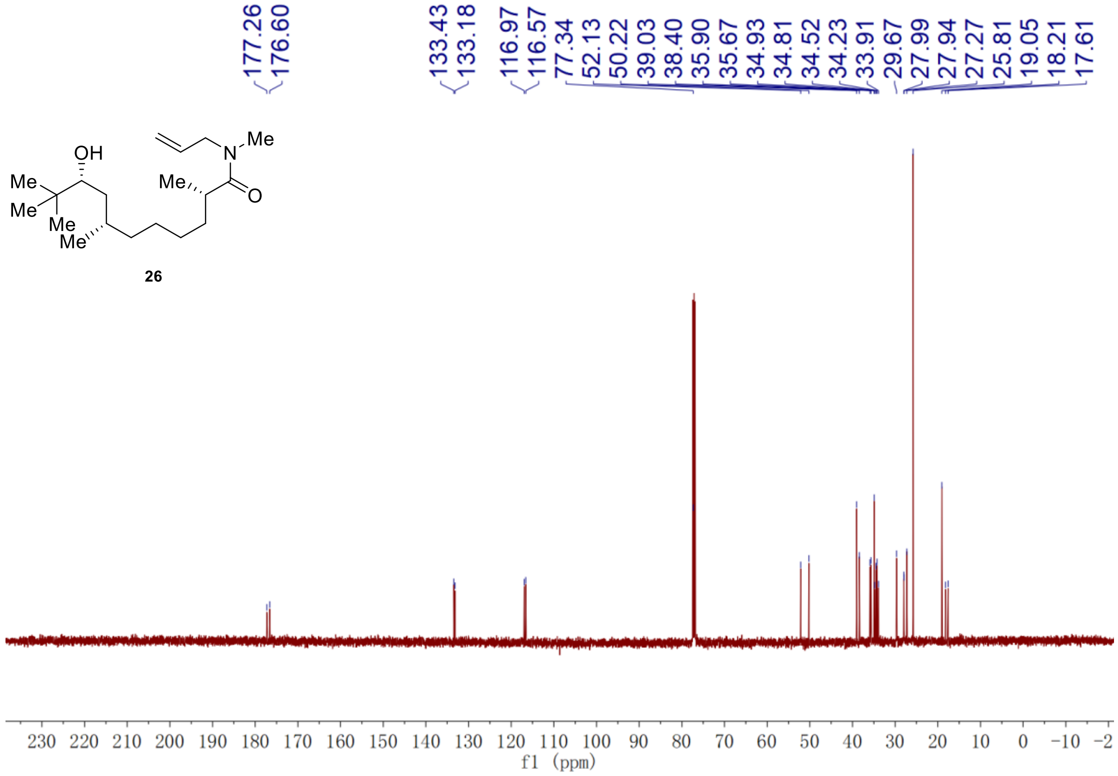

^{1}H NMR Spectrum of **27** (500 MHz, $CDCl_3$)

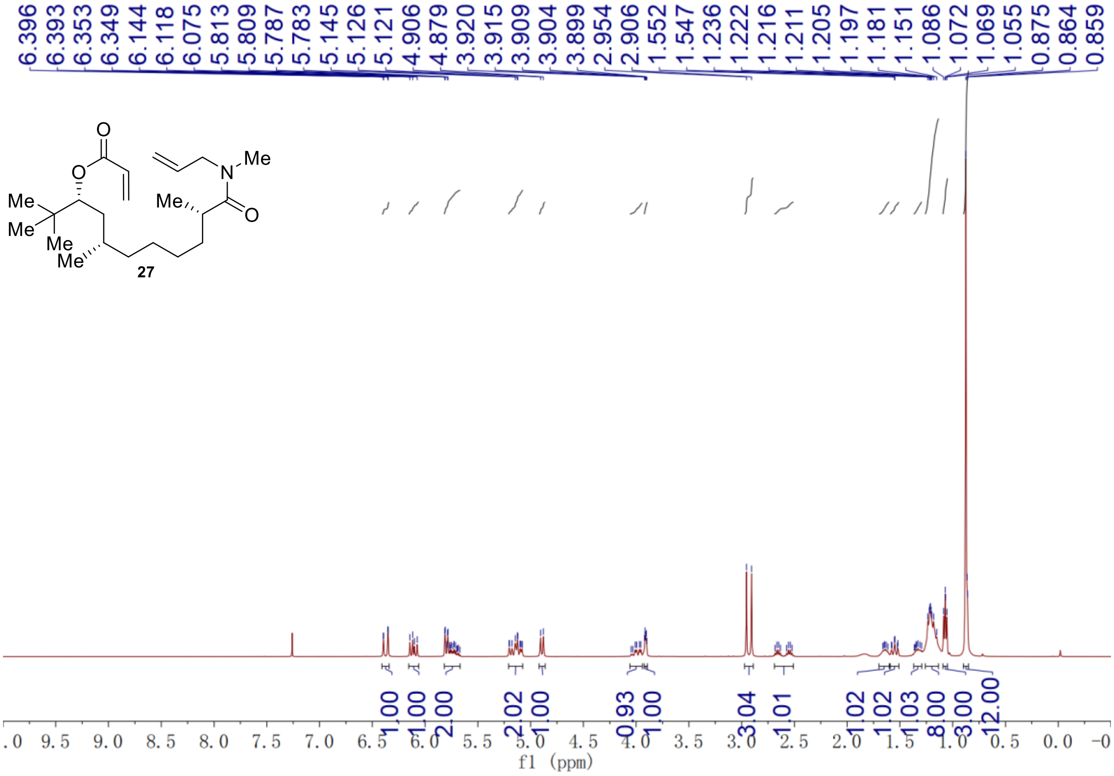

^{13}C NMR Spectrum of **27** (125 MHz, $CDCl_3$)

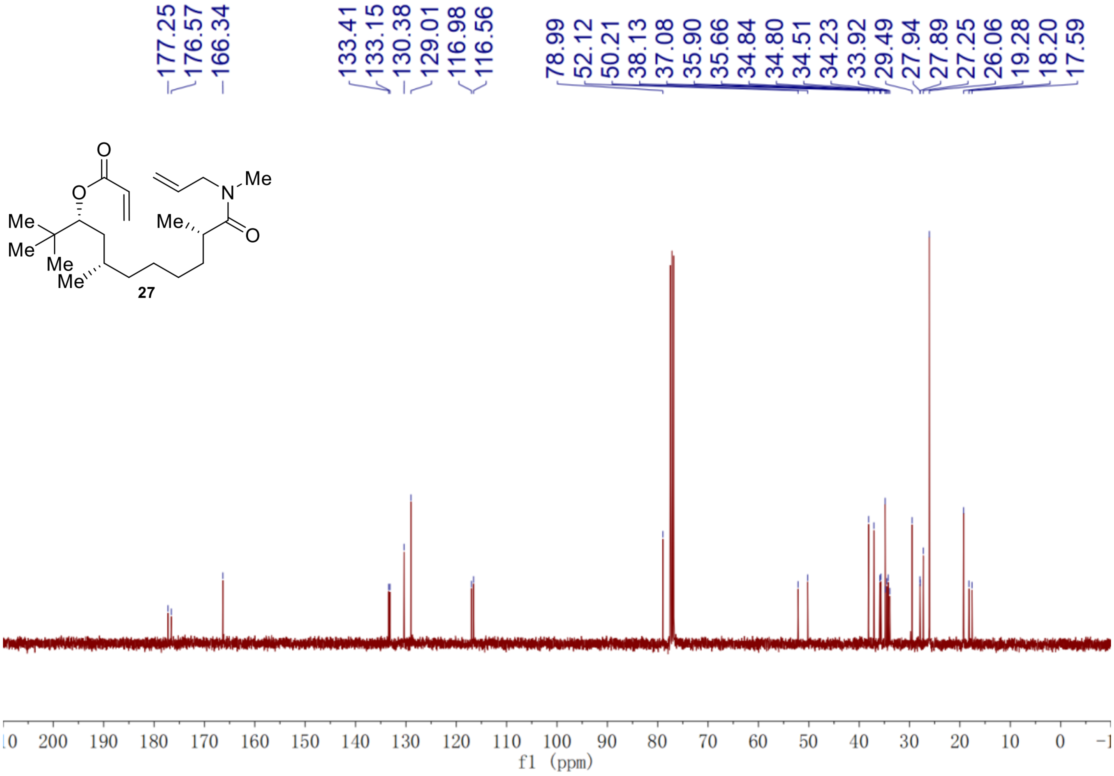

¹H NMR Spectrum of (2*S*,7*S*,9*R*)-laingolide A **(1c)** (500 MHz, $CDCl_3$)

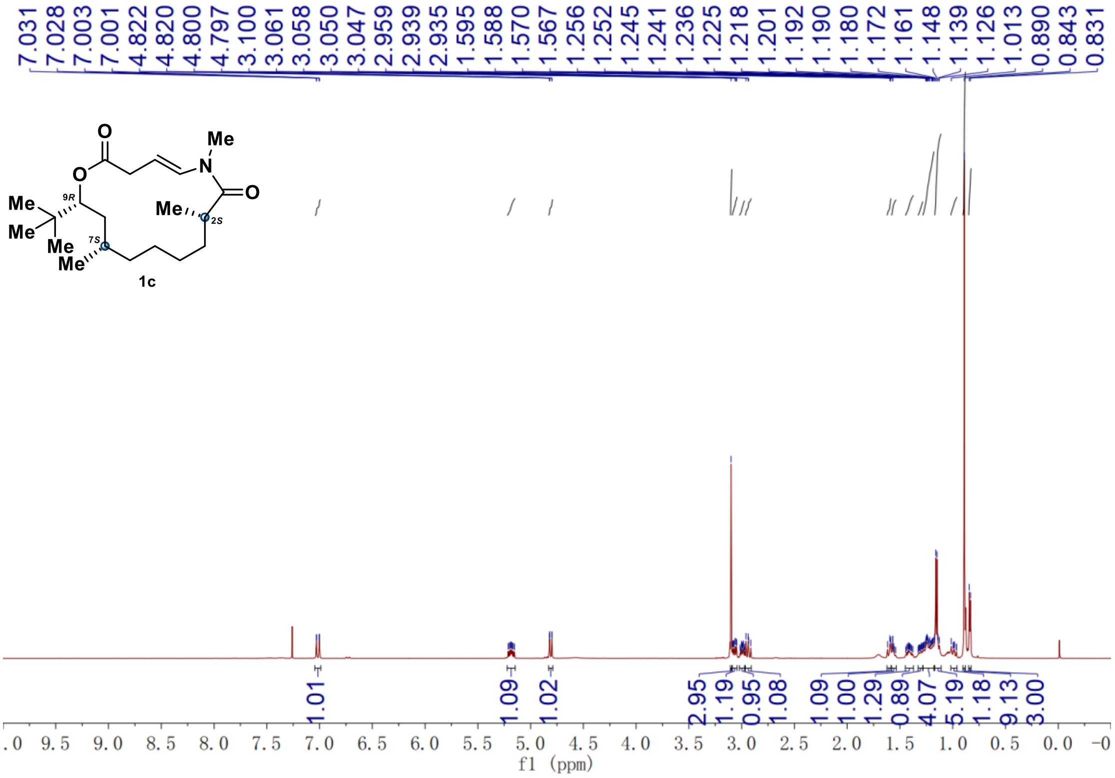

¹³C NMR Spectrum of (2*S*,7*S*,9*R*)-laingolide A **(1c)** (125 MHz, $CDCl_3$)

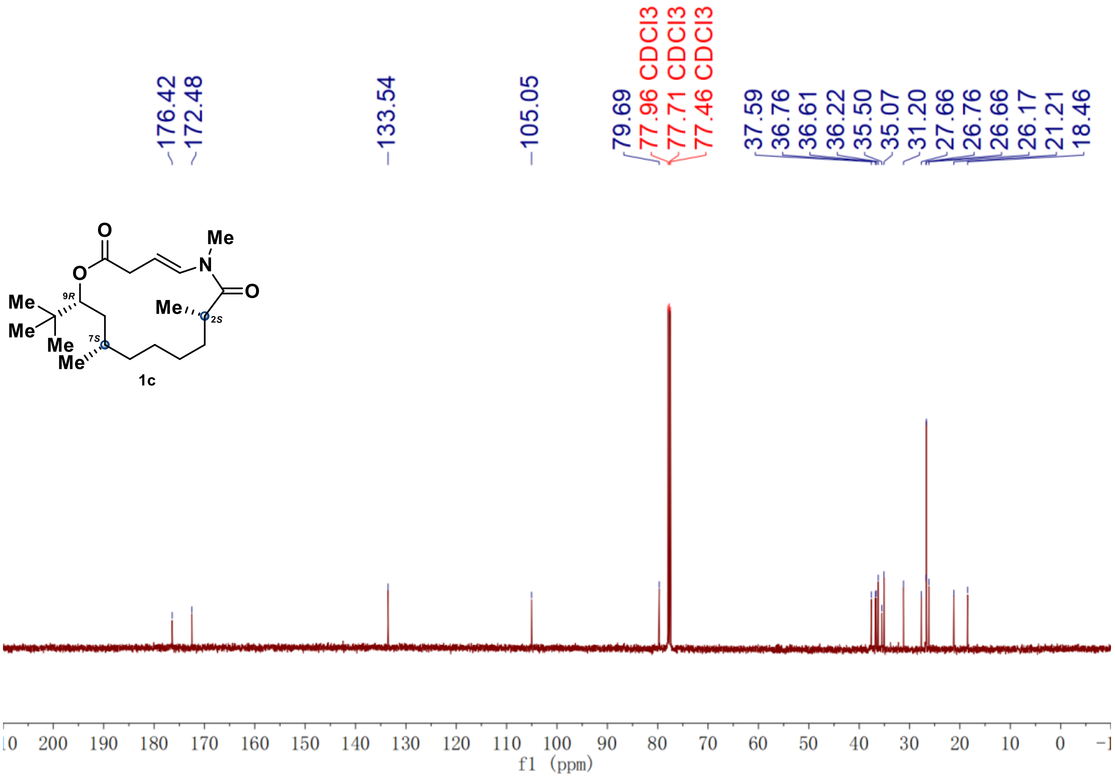

^{1}H NMR Spectrum of **28** (400 MHz, $CDCl_3$)

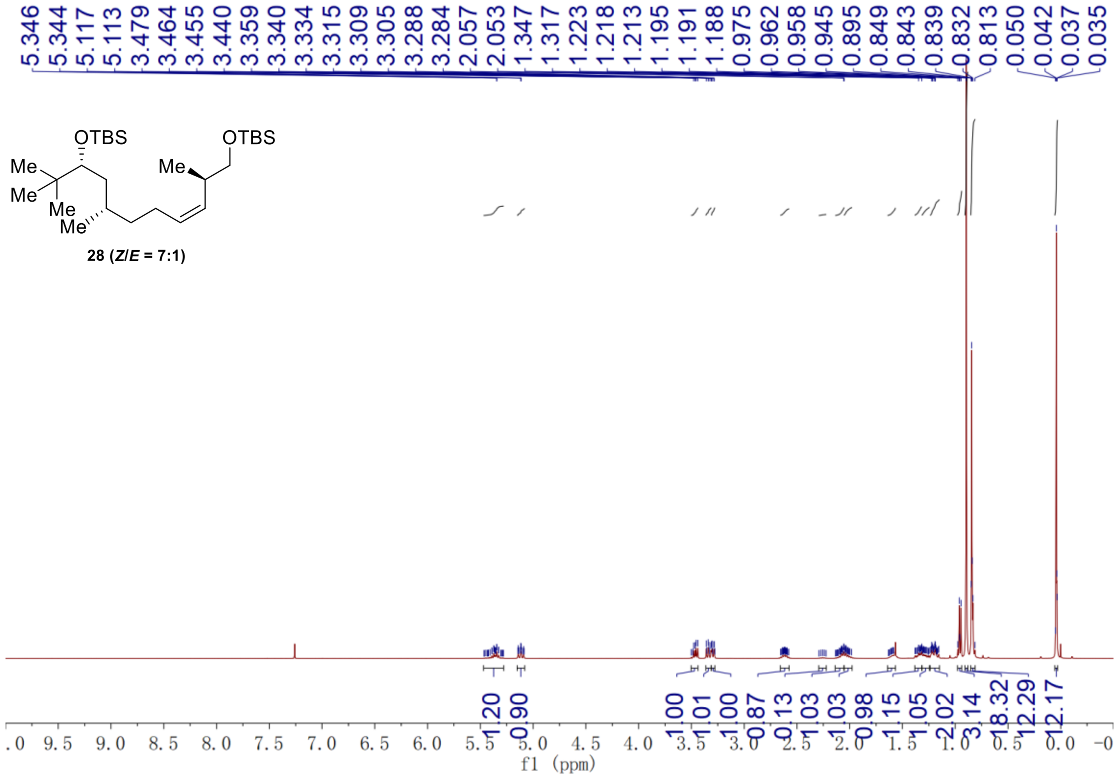

^{13}C NMR Spectrum of **28** (100 MHz, $CDCl_3$)

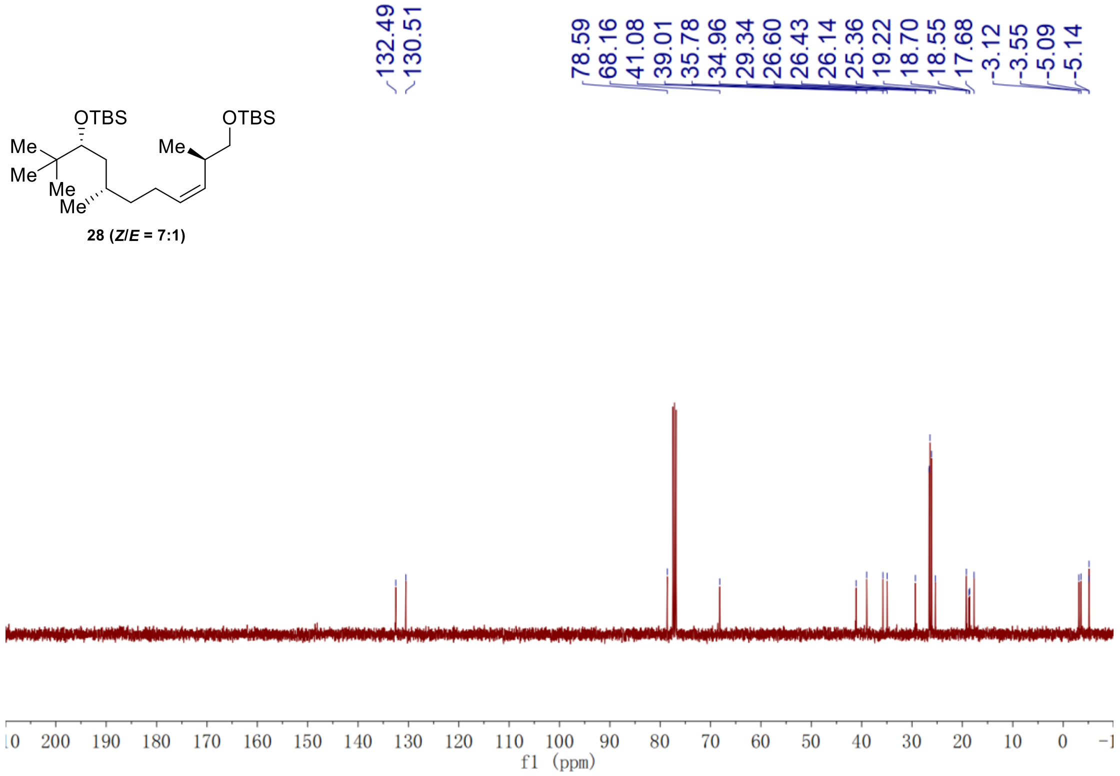

¹H NMR Spectrum of **29** (400 MHz, $CDCl_3$)

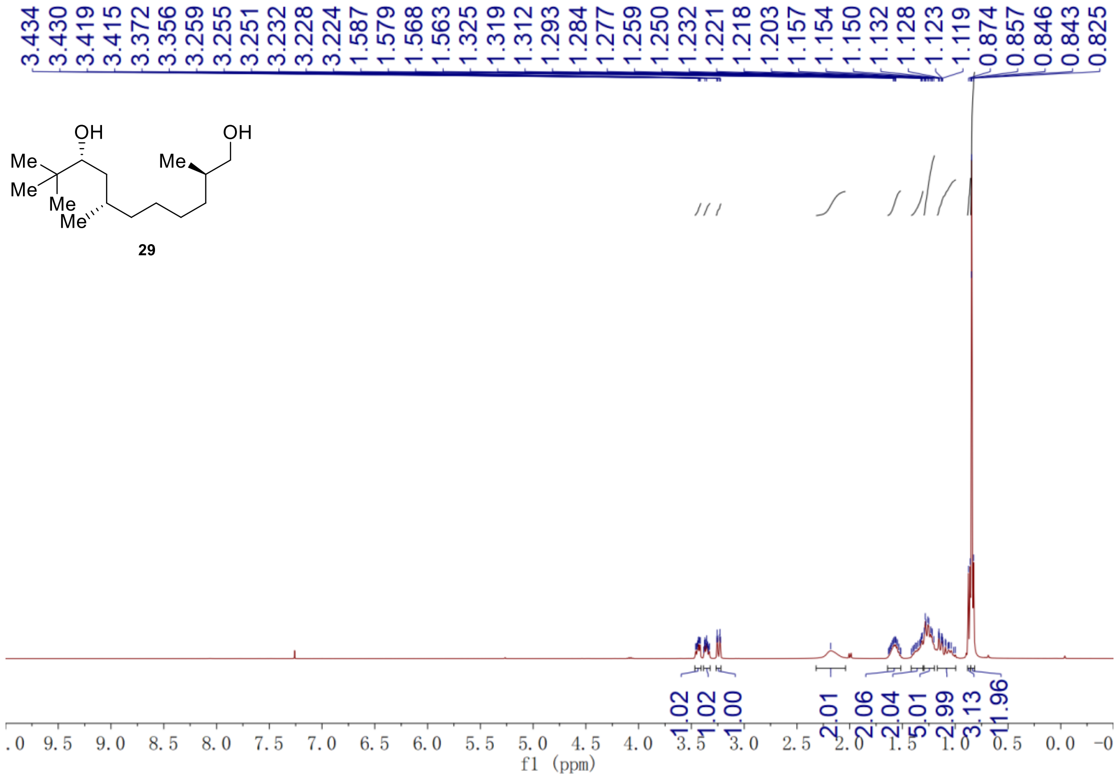

¹³C NMR Spectrum of **29** (100 MHz, $CDCl_3$)

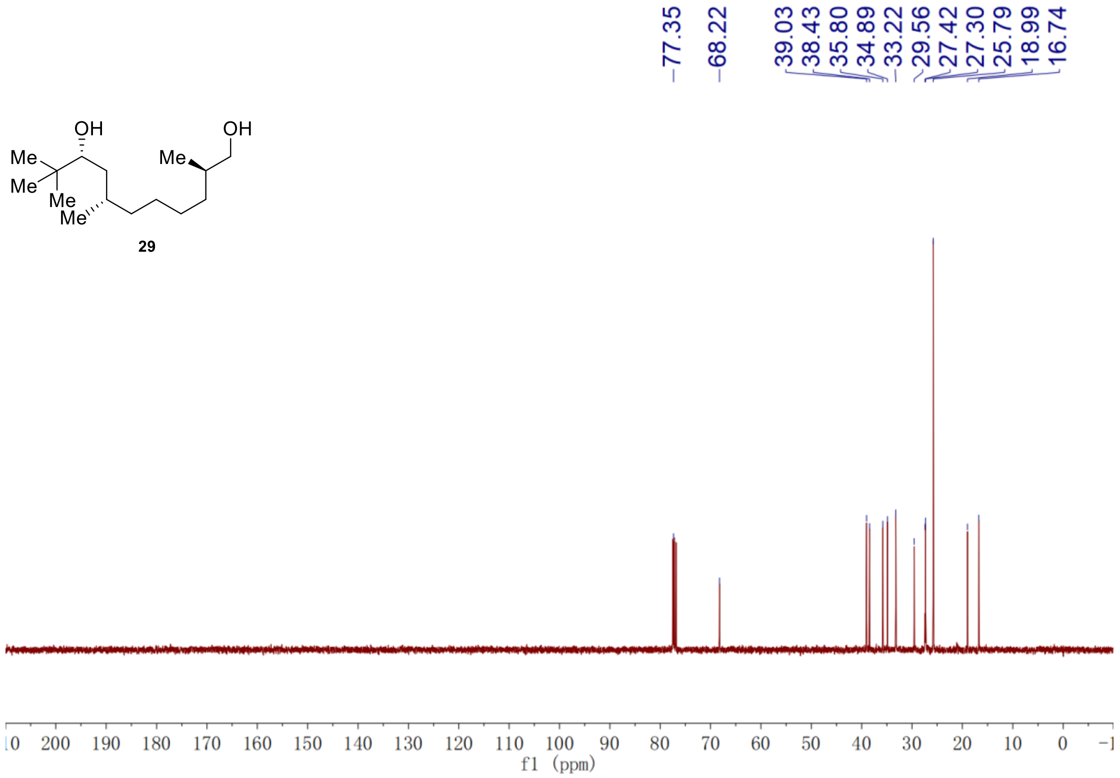

^{1}H NMR Spectrum of **S20** (500 MHz, $CDCl_3$)

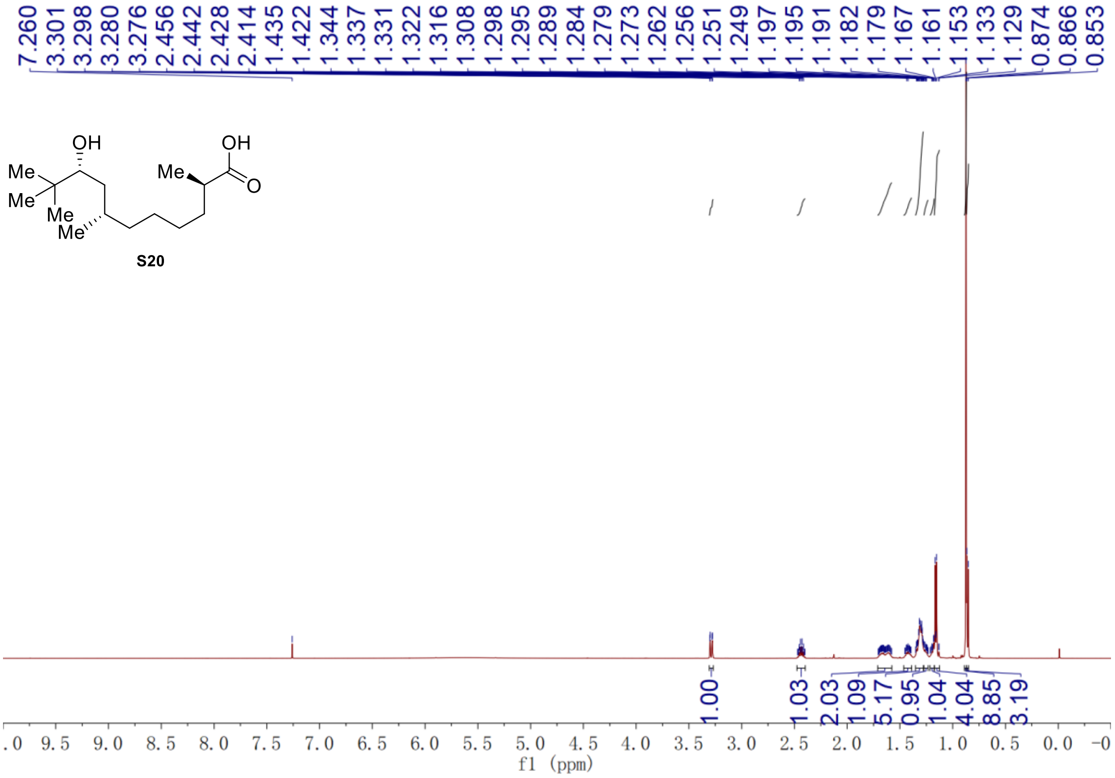

^{13}C NMR Spectrum of **S20** (125 MHz, $CDCl_3$)

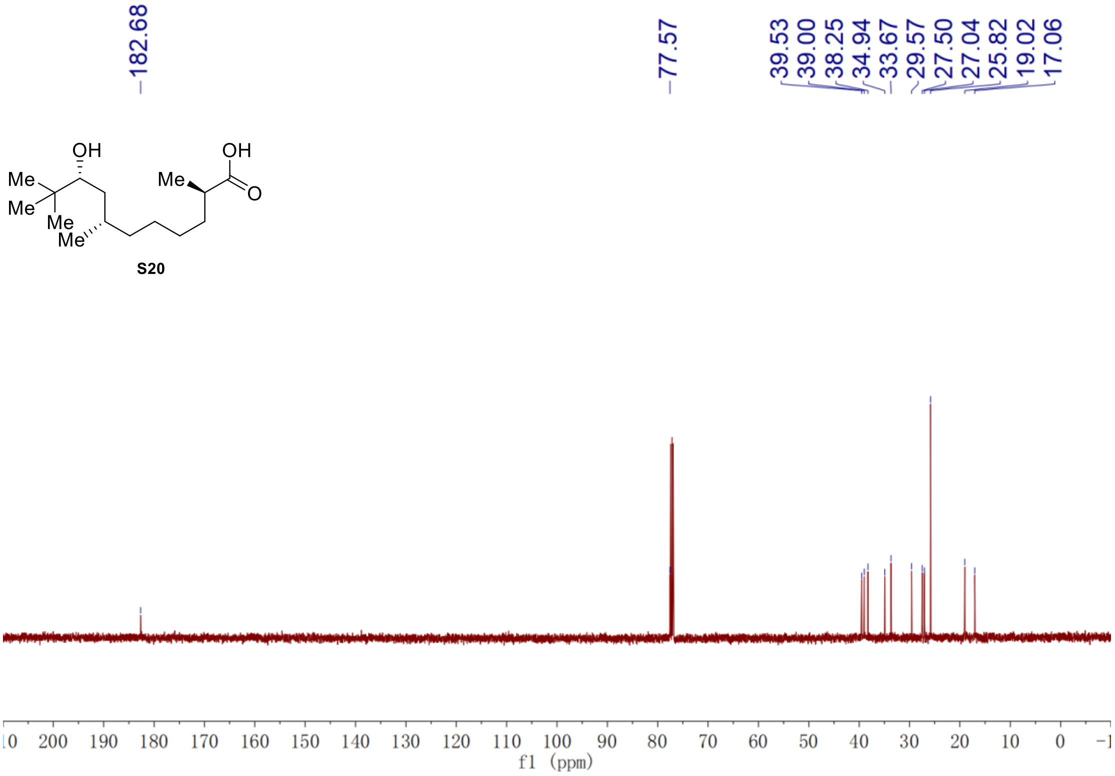

^{1}H NMR Spectrum of **30** (400 MHz, $CDCl_3$)

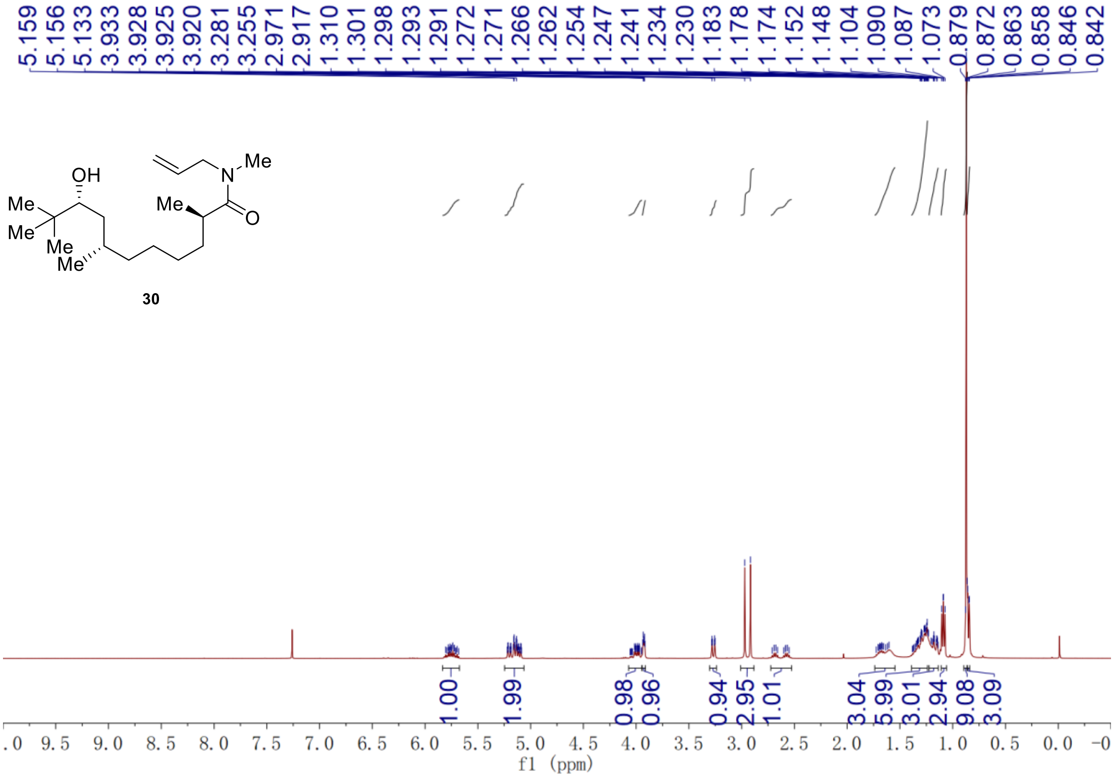

^{13}C NMR Spectrum of **30** (100 MHz, $CDCl_3$)

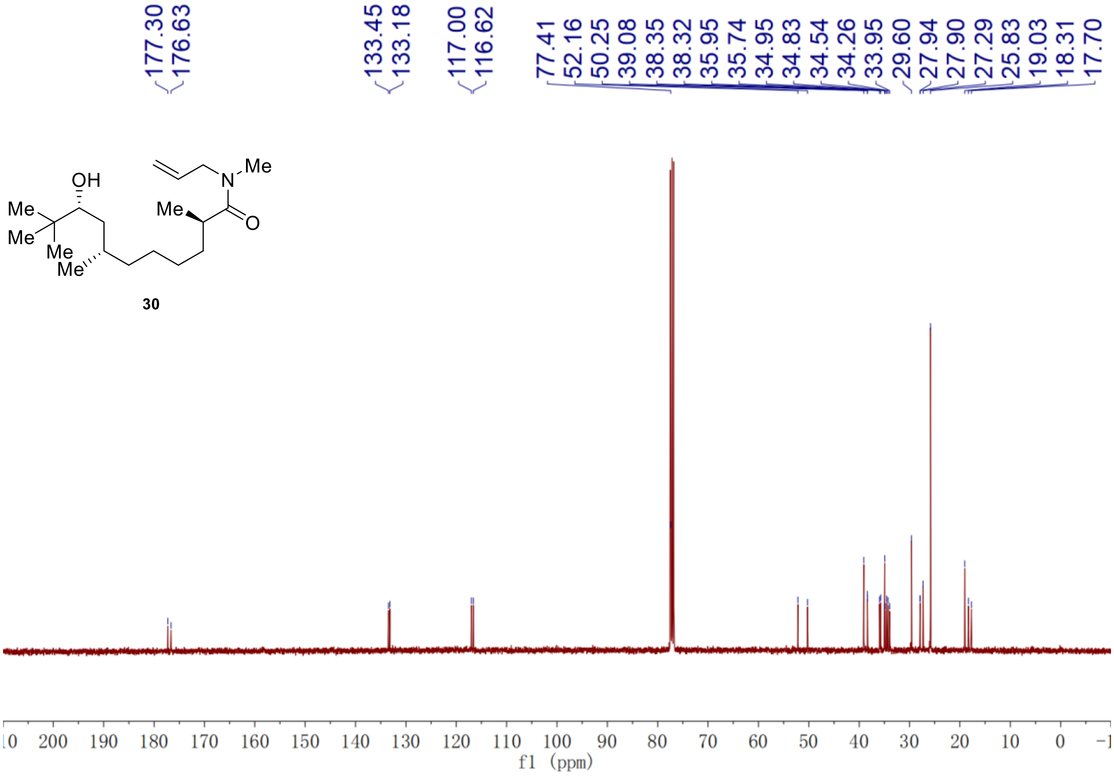

^{1}H NMR Spectrum of **31** (400 MHz, $CDCl_3$)

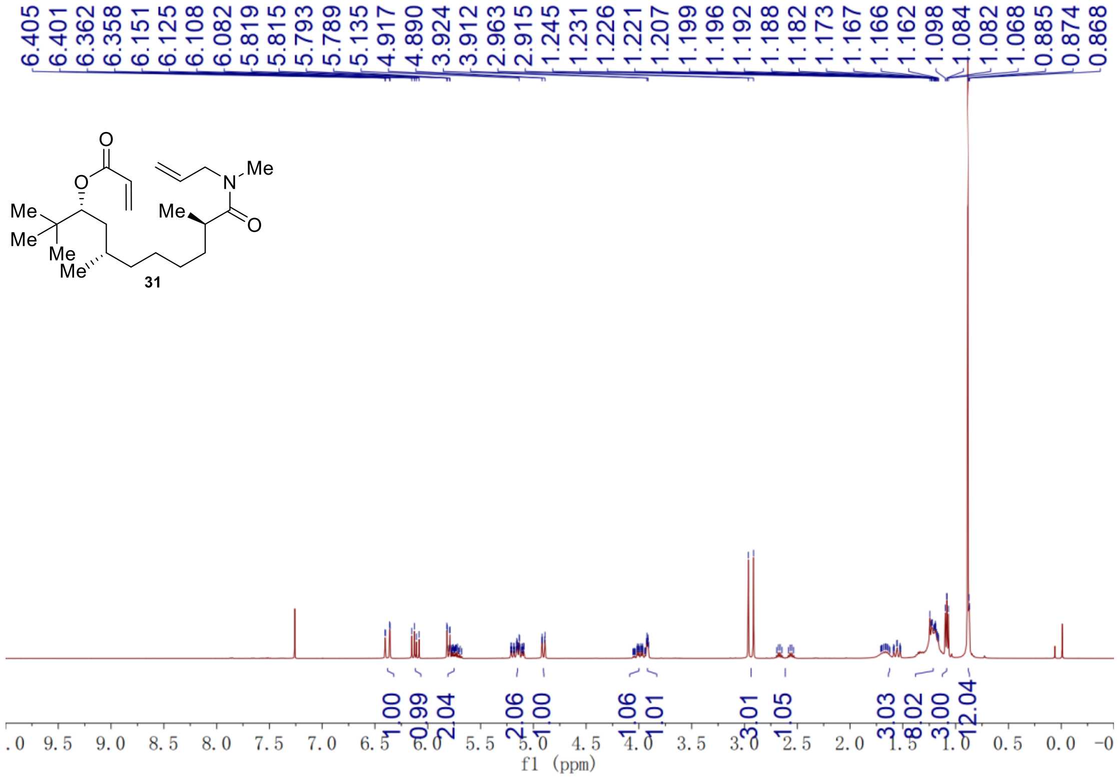

^{13}C NMR Spectrum of **31** (100 MHz, $CDCl_3$)

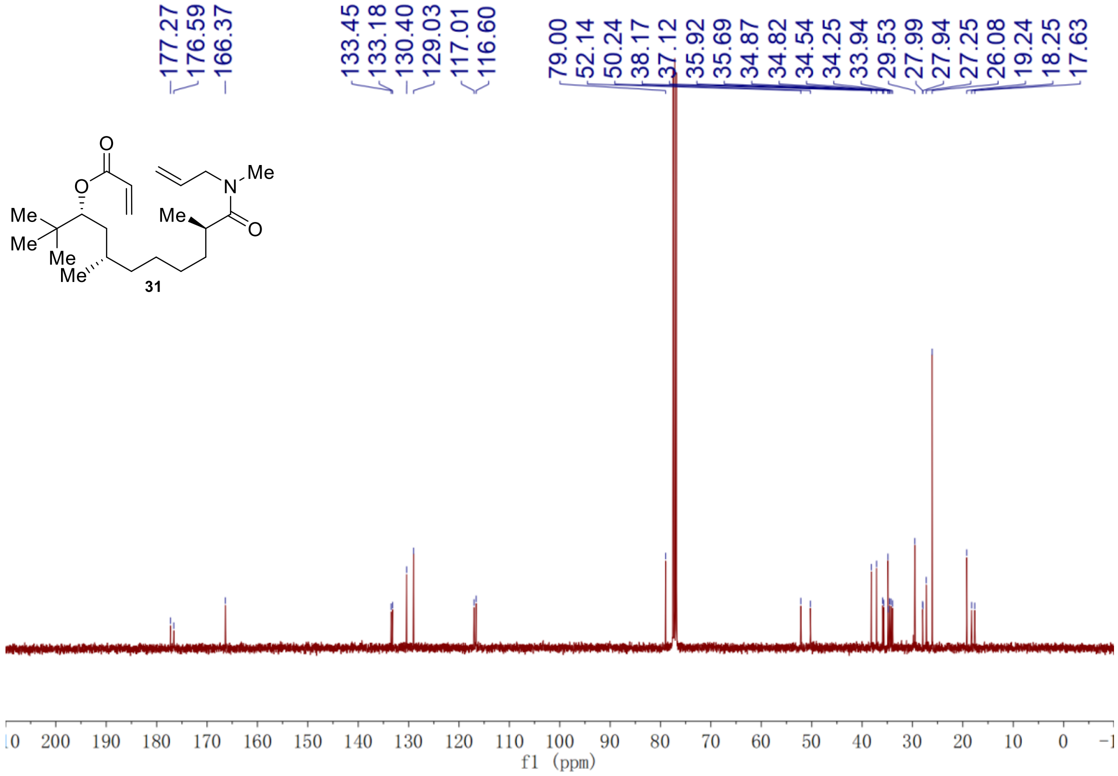

^{1}H NMR Spectrum of (2*R*,7*S*,9*R*)-laingolide A **(1d)** (400 MHz, $CDCl_3$)

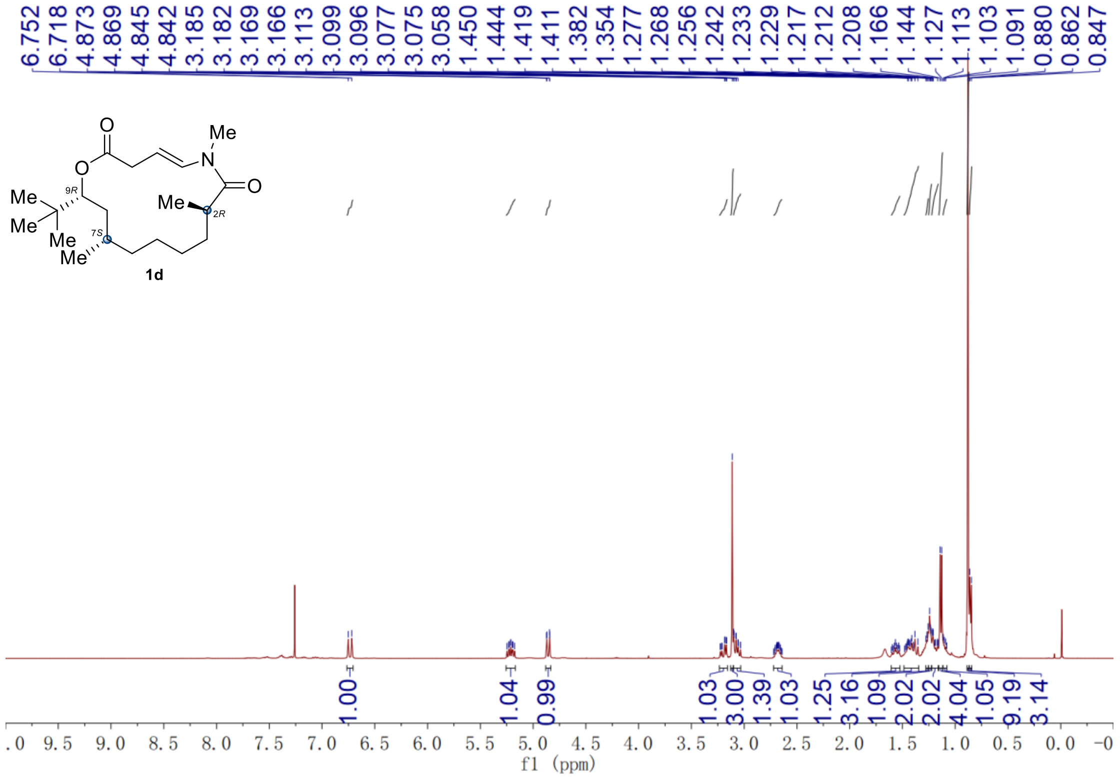

^{13}C NMR Spectrum of (2*R*,7*S*,9*R*)-laingolide A **(1d)** (100 MHz, $CDCl_3$)

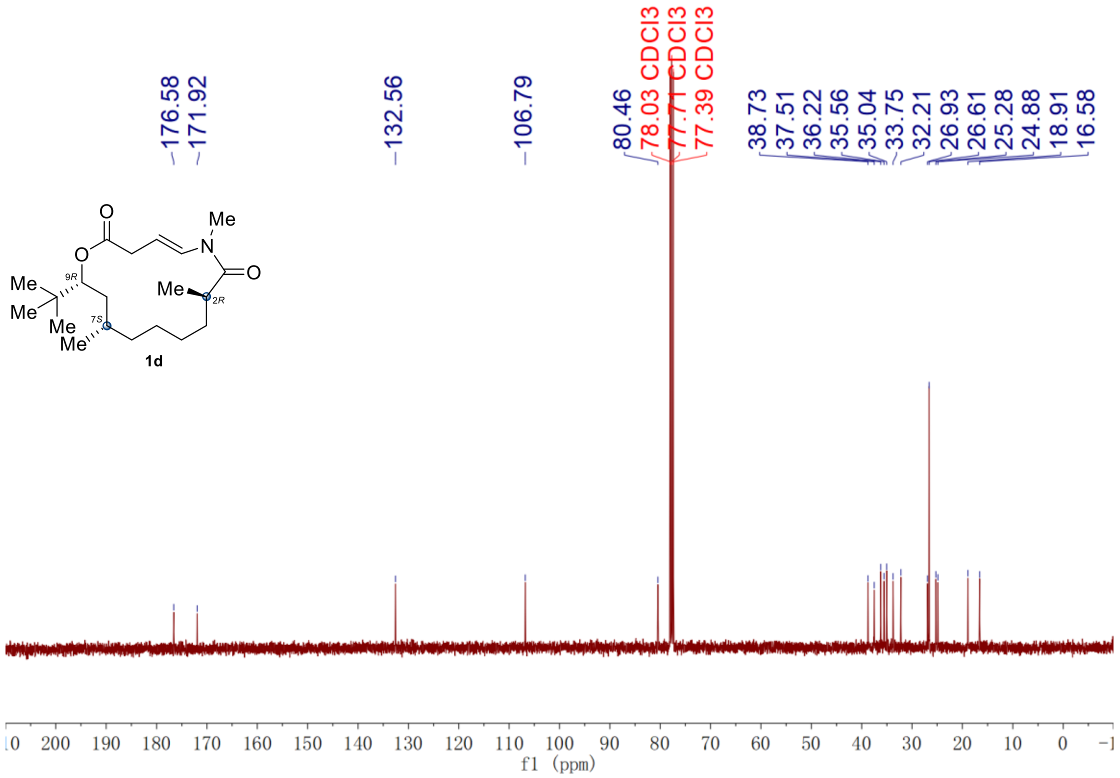

Supplement: Supplementary file 1 [file marinedrugs-19-00247-s001.zip › marinedrugs-1195079-supplementary.pdf]
